# Supplementary material for: Ancient Rapanui genomes reveal resilience and pre-European contact with the Americas
Source: Nature. 2024 Sep 11;633(8029):389–97. doi: 10.1038/s41586-024-07881-4 (PMC11390480; doi:10.1038/s41586-024-07881-4)
Supplement: Supplementary file 1 — Supplementary Notes 1–15 (with detailed descriptions of laboratory procedures, computational analyses and validation experiments), Figs. 1–51 and Tables 2 and 22–24. [file 41586_2024_7881_MOESM1_ESM.pdf]

nature portfolio

https://doi.org/10.1038/s41586-024-07881-4

Supplementary information

Ancient Rapanui genomes reveal resilience and pre-European contact with the Americas

In the format provided by the authors and unedited

Ancient Rapanui genomes reveal resilience and pre-European contact with the Americas

Supplementary Information

S1. Laboratory procedures 2
S2. Radiocarbon dating 5
S3. Ancient DNA sequencing data mapping, assessment and authentication 8
S4. Reference data 15
S5. Imputation accuracy of 'Ancient Rapanui' genomes 17
S6. Multidimensional scaling 27
S7. *f*-statistics 29
S8. Identity-by-descent segment sharing 48
S9. Relatedness analysis of 'Ancient Rapanui' individuals 52
S10. Runs of homozygosity (ROH) 56
S11. Effective population size and population collapse 62
S12. Model-based clustering analyses (*ADMIXTURE*) 80
S13. Local ancestry inference 85
S14. Admixture dating using genetic data 91
S15. Admixture dating using genetic and C14 data jointly 103

S1. Laboratory procedures

S1.1. Sampling

We sampled eleven petrous bones and four teeth originating from fifteen individuals stored at the Muséum National d'Histoire Naturelle, Musée de l'Homme in Paris after collection in 1872-1877 or 1935 (Ethics and inclusion). According to the museum archives, the individuals originate from Rapa Nui. Small amounts of petrous bone samples were drilled into powder (92-122 mg) at the clean laboratory facilities of the Musée de l'Homme (Paris, France). Part of the bone powder (47-67 mg) of the petrous samples was kept for C14 dating, performed at the ORAU (Oxford Radiocarbon Accelerator Unit) and BRAMS (Bristol Radiocarbon AMS) (Section S2). The teeth were sent to the Globe Institute clean lab facility at University of Copenhagen, Denmark, where the root caps were split from the crowns using a diamond-dust-coated cutting disc and pulverized with a mortar (670-985 mg/sample).

S1.2. DNA Extraction, library preparation and sequencing

All the samples were processed in dedicated ancient DNA laboratories at the Globe Institute, University of Copenhagen, using two different DNA extraction (that we label 'EP1' and 'EP2' below) and library preparation protocols ('LP1' and 'LP2' below, see also Table S1). Negative controls were included for every step described below.

S1.2.1. EP1: Teeth extraction

Teeth were extracted following [1]. Similar to the petrous samples, the pulverized teeth samples were pre-digested in 2 mL extraction buffer AL01 (0.5M EDTA, TE buffer 100x, Proteinase K, N-laurylsarcosine (10%), phenol red) for 15 minutes as in [2]. Following pre-digestion, the teeth were incubated overnight rotating at 37°C in 4 mL extraction buffer AL01. Next, samples were purified using silica beads (SiO2 particles, HCl) in binding buffer BL01 (phenol red, 5M NaCl, 5M Na acetate and PB (5M GuHCl, 30% 2-propanol), pH=4-5) and incubated for 1 hour at room temperature while rotating. Then, the samples were washed twice in 80% ethanol and dried for 10 minutes. Lastly, they were resuspended in TEB buffer (Qiagen EB supplemented with 0.05% Tween-20) and incubated at 37°C for 15 minutes. The supernatants were transferred to 1.5mL Eppendorf tubes and spun down to remove residual SiO2 beads.

S1.2.2. EP2: Petrous bone extraction

Petrous bones were extracted following [3] including a pre-digestion step as in [2]. All samples were pre-digested for 10 minutes in 1 mL digestion buffer DB1 (0.45 M EDTA, 0.25 mg/mL Proteinase K). Following the pre-digestion step, the samples underwent an overnight digestion step in 4 mL digestion buffer DB1. After digestion, the samples were purified using silica pellets in binding buffer BB1 (5 M Gu-HCL, 100 mM NaOAc, 20 mM NaCl, 30% isopropanol). Then, the samples were washed twice in ice-cold 80% ethanol and

resuspended in EB buffer (10 mM Tris-Cl). Finally, the beads were magnetically separated to obtain clean DNA extract.

S1.2.3. USER treatment

Prior to library preparation, a portion of all the extracts were USER-treated to reduce the effect of C-to-U damage, a characteristic of ancient DNA damage [4]. The USER protocol consists of two phases: an uracil excision part and a USER Enzyme deactivation and reaction clean-up part. A reaction mixture was prepared with the following amounts of reagents per sample: 6 µL of 10x Tango buffer, 0.24 µL dNTP mix (25 mM), 0.6 µL ATP (100 mM), 3.6 µL USER Enzyme (2 U µL−1). The reaction was mixed by finger-flicking, briefly spun down before adding 15-25 µL of DNA extract to the mixture and mixing it again. Depending on the amount of DNA extract used, molecular grade water was added to reach a final reaction volume of 47 µL. The reactions were then incubated on a thermo-cycler for 30 minutes at 37°C. After incubation, deactivation of the USER enzyme was performed by adding 3 µL of UGI (2 U µL−1) to each sample, mixing, centrifuging and incubating again at 37°C for 30 minutes. The clean-up of this reaction was performed by adding 80-90 µL (1.6-1.8x of reaction volume) of MagBio or SPRI bead, mixing and incubating the reaction for 5 minutes at room temperature and washing twice with 200 µL of ethanol. Lastly, DNA was eluted using 22 µL of EBT, incubating it first in a normal rack and then a magnetic rack and storing the supernatant. In order to determine the adapter concentration to use in the next step, DNA concentration of each sample was measured using Qubit.

S1.2.4. LP1: Double-stranded DNA (dsDNA) libraries

Teeth extracts that were not USER-treated (n=4) were built into double-stranded libraries following the Illumina library preparation protocol in [5]. The samples were end-repaired in 40 µL reactions with 32 µL of DNA extract. The reaction was incubated 30 minutes at 20°C, followed by 30 min at 65°C. Next, 2 µL of adapters were ligated to the end-repaired DNA using T4 DNA ligase (NEB 400U/µL) in 50 µL reactions. These were incubated for 30 minutes at 20°C and 10 minutes at 65°C. The fill-in reaction was performed by adding 10 µL of the fill-in master mix and incubating the reactions for 15 minutes at 65°C followed by 15 minutes at 80°C. Finally, the libraries were purified using SPRI magnetic beads [6] and eluted in 60 µL EB.

S1.2.5. LP2: Single-stranded DNA (ssDNA) libraries

All the USER-treated extracts and some non-USER-treated extracts (Table S1) were built into single-stranded libraries following [7]. Briefly, the sample mix was prepared by adding 2 µL of input-specific single strand binding proteins (SSB's) dilution to 20 µL of DNA extract and incubating the mixture for 3 minutes at 95°C in a thermocycler. After this, the samples were quickly shocked in an ice-bath so that they stayed single-stranded. Following the denaturation, 1 µL of input-specific library adapters P5 and P7 and 26 µL of Santa Cruz Reaction master mix (SCR master mix) was added to each sample. The libraries then underwent a cleaning process with the use of MinElute columns or MagBio beads. Both

purification methods were used on these libraries, on different batches. The purification process using MinElute columns used 500 μL of binding buffer (BB1) to bind the DNA with the filter placed in the bottom of the column. After this step, 53 μL of elution buffer (EB) was added and the library was transferred into a fresh 1.5 mL Eppendorf tube which was stored at -20℃. Purification using MagBio beads was the same as described above in the USER-treatment clean-up. Negative library controls accompanied each library preparation session.

S1.2.6. qPCR and sequencing

Quantifying PCR reactions were conducted with 1 μL of each sample to determine the optimal amount of PCR cycles using Taq Gold Polymerase and KAPA HiFi HotStart Uracil+ Readymix. The libraries were then amplified (7-17 PCR cycles) and indexed using a dual-indexing approach [8] using KAPA and following the standard thermal cycling conditions for this enzyme.

All sequencing was carried out at the GeoGenetics Sequencing Core facility, Globe Institute, University of Copenhagen. For screening, the libraries were sequenced on one lane of Illumina HiSeq 4000. Additionally, four runs of Illumina NovaSeq S4 were performed: one full flowcell of all SCR libraries, one lane of the UDG-treated libraries and two full flowcell runs of selected libraries (Table S1).

S2. Radiocarbon dating

Radiocarbon measurements were undertaken at the ORAU (Oxford Radiocarbon Accelerator Unit) and BRAMS (Bristol Radiocarbon AMS) facilities. The results are shown in Table S2.

Results are reported in radiocarbon years Before Present (BP) and corrected for isotopic fractionation. Stable isotope measurements ($\delta^{13}C$ and $\delta^{15}N$) from bone collagen were obtained from all individuals dated in Oxford for palaeodietary estimates using an EA-IRMS. In Bristol the $\delta^{13}C$ values were generated from the AMS only and therefore may be subject to additional uncertainty in terms of palaeodiet estimates (see below). Samples of bone were pretreated according to [9,10].

Humans living on Rapanui often reveal evidence for the consumption of marine resources, which have a reservoir age affecting radiocarbon dates. This is because the age between contemporaneous living samples in the sea and on land is, on average, 400 years different, with the marine material being older. To correct for this, we used a `Mix_Curve` approach in OxCal [11]with the Marine20 calibration dataset, which corrects for this average offset, and to account for further local variations we calculated a ΔR value of -214 ± 36 years, derived from the data in [12,13]. To estimate the proportion of marine protein in the diet of the dated humans we used the method of [14] and linearly interpolated bone collagen $\delta^{13}C$ values between an inferred 100% terrestrial diet (-21.6‰) and a 100% marine diet (-12.4‰), with the end-point values obtained following [15]. With the `Mix_Curve` method, we applied to the mixing estimates an uncertainty on each value of 10% (after [14]). In cases where we had no stable carbon isotope measurements (the 5 BRAMS measurements in Table S2), we estimated the marine contribution by making some assumptions based on previously published data and the samples in our study for which we have measurements. Even though this is challenging, we followed [16] who estimated the marine contribution on Rapa Nui to be 35% when bulk collagen is analysed. When we calculate the average of our own measured stable isotope data we find the value to be 35.2%, which is encouraging, so for samples which have no measured $\delta^{13}C$ values we therefore used an estimated value of 35% ± 10% to account for the offset in marine carbon. The calibrated age ranges, corrected for the marine contribution using the `Mix_Curve` method are shown in Figure S1.

In Section S15 below, we describe our Bayesian modelling of this dataset.

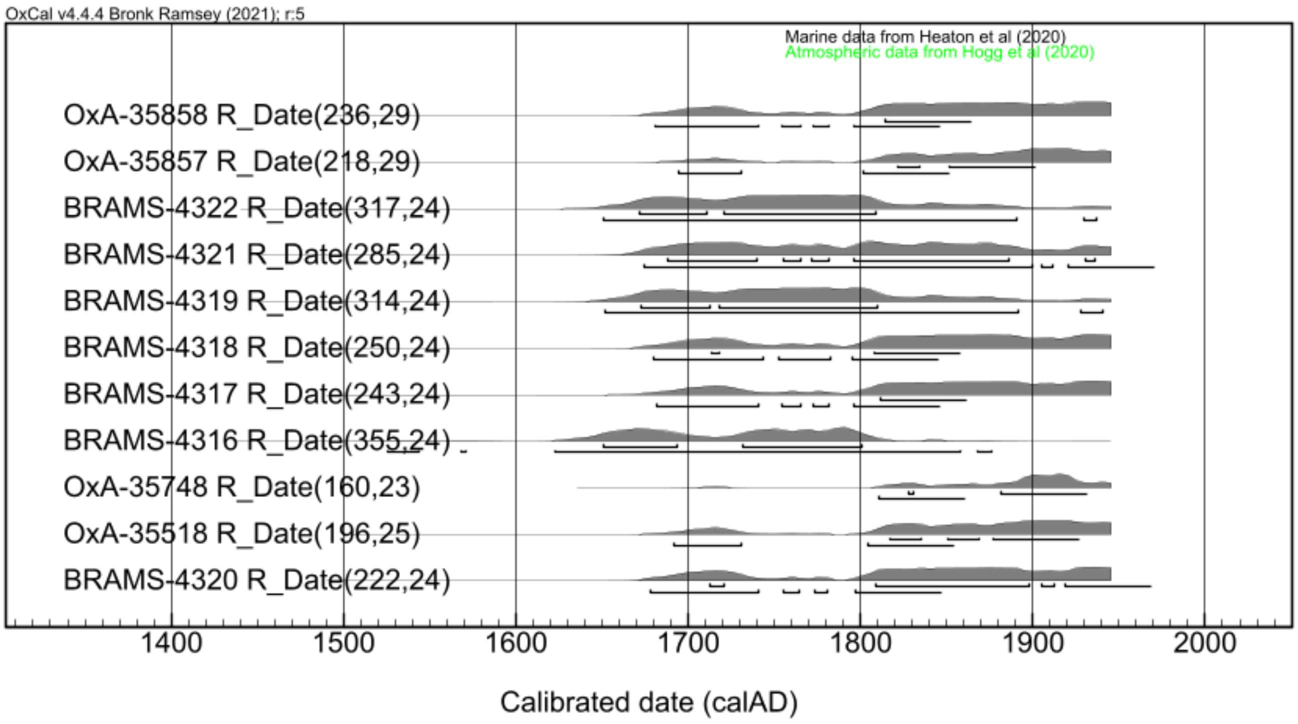

Figure S1. Calibrated AMS determinations obtained using the Mix_Curves method in OxCal 4.4.

Lab codes	Location	ID	Tissue	Collection	Date collect.	Museum ID	C14 Age	± value	$\delta^{13}C$ (per mille)**	$\delta^{15}N$ (per mille)
BRAMS-4316	Vaihou	RN06	petrous	Pinart	1877	6765	355	24		
BRAMS-4317	Vaihou	RN08	petrous	Pinart	1877	6769	243	24		
BRAMS-4318	Vaihou	RN10	petrous	Pinart	1877	6780	250	24		
BRAMS-4319	Vaihou	RN11	petrous	Pinart	1877	6790	314	24		
BRAMS-4320	Baie de la pérouse	RN12	petrous	Pinart	1877	6797	222	24	-19.26	
BRAMS-4321	Baie de la pérouse	RN14	petrous	Métraux	1935	19487	285	24	-18.22	
BRAMS-4322	Baie de la pérouse	RN15	petrous	Métraux	1935	19496	317	24		
OxA-35518	Baie de la pérouse	RN02	tooth	Pinart	1877	6128	196	25	-18.98	14.2
OxA-35748	Vaihou	RN01	tooth	Pinart	1877	6771	160	23	-17.55	13.7
OxA-35857	Baie de la pérouse	RN03	tooth	Métraux	1935	19489	218	29	-17.64	14.7
OxA-35858	Baie de la pérouse	RN04	tooth	Métraux	1935	19492-1	236	29	-18.51	13.9

Table S2. Radiocarbon determinations and stable isotope data obtained from in this study.

S3. Ancient DNA sequencing data mapping, assessment and authentication

S3.1. Basecalling and adapter removal

CASAVA 1.8.2. (Illumina) was used to produce base calls at the GeoGenetics Sequencing Core facility and sequencing reads were demultiplexed by requiring perfect matches to the dual indexes corresponding to each library. We trimmed Illumina adapter sequences, leading Ns (-- trimns) and trailing quality 2 runs (--trimqualities --minquality 2) using *AdapterRemoval* v1.5.3 [17]. Overlapping pairs were collapsed (--collapse) requiring a minimum of 11 overlapping bases and only collapsed reads ≥30bp were retained for downstream analyses following [18,19].

S3.2. Mapping

We followed the read mapping strategy in [18]. In brief, we mapped filtered reads to the human reference genome (build 37) using *bwa aln* v0.6.2-r126 [20] with disabled seeding (-l parameter) [21]. We excluded reads with mapping quality <30 and we removed PCR duplicates using *picard-tools MarkDuplicates* (http://picard.sourceforge.net). We conducted local realignment using GATK [22] and computed the MD tag and extended BAQ for each read using the *samtools calmd* command [23]. To minimise potential error introduced by *post-mortem* damage [24], for population genetics analyses we excluded five bases from both ends of reads derived from non-UDG-treated extracts and two bases from both ends of reads derived from UDG-treated extracts [4].

S3.3. Chromosomal sex determination

To determine the chromosomal sex of the ancient individuals, we followed the rationale in [25]. Assuming that the X chromosome can be mapped as easily as the autosomes, we expect that the depth of coverage for the X chromosome is either equal to the average genome coverage (XX individuals) or half the genome coverage (XY individuals).

Following [25], we classified individuals as XY if Rx+1.96SE (Rx: ratio of the number of reads mapping to the X chromosome and to the autosomes; SE: standard error computed using the 22 autosomes) was below 0.6, and as XX if Rx-1.96SE was above 0.80. Eight of the individuals were classified as XY and seven were classified as XX (Figure S2).

S3.4. Ancient DNA authentication

S3.4.1. *Post-mortem* damage patterns

We used *bamdamage* [26] to examine the fragment length distribution and nucleotide misincorporation patterns in each library to assess whether sequencing reads derive from authentic ancient DNA fragments [24]. For all libraries we recorded short mean insert sizes (52-78bp) and an excess of C-to-T substitutions towards both ends of the reads. For single-stranded libraries [27] in particular, which constitute most of the sequenced libraries, we observe predominant C-to-T deamination at both ends of the reads for all libraries. In Figure

S3, we show ancient DNA damage patterns for three representative libraries and present mean fragment lengths and 5' deamination rates in Table S1.

S3.4.2. Error rates

We estimated overall and type-specific error rates for the 15 'Ancient Rapanui', as well as for a high-coverage present-day Rapanui genome (P2077 [28]), used here as a reference. The error rates were estimated with *ANGSD* v0.930 [29] (-doAncError 1) using the chimp as an outgroup and a high-quality present-day 38x Dinka genome (SS6004480) from the SGDP dataset [30]. This approach estimates the error rates from the excess of derived alleles present in the target genomes when compared to the high-quality genome, where the outgroup is assumed to carry the ancestral allele [31].

Figures S4 and S5 show the estimated error rates estimates for all individual samples and per library, respectively. Consistent with the deamination patterns, the error rates for the ancient individuals are highest for C-to-T (and G-to-A reflecting the reverse strand) substitutions. This trend is absent from the present-day Rapanui genome (Figure S4). The error rates estimated for the ancient genomes are one order of magnitude higher than the error rate value obtained for P2077 (0.08%) for most ancient genomes. RN05 and RN06 have particulary high error rates (1.05% and 1.13%, respectively) and RN13 has a similar error rate to P2077. As expected, UDG-treated libraries had lower error rates than non-UDG-treated ones (0.10%-0.27% vs. 0.44%-1.23%).

S3.4.3. Contamination

S3.4.3.1. Mitochondrial DNA contamination estimates

We inferred mitochondrial DNA contamination in the 'Ancient Rapanui' using *contamMix* [32], which models sequencing reads as deriving from a mixture of the endogenous consensus mtDNA and a set of 311 possible contaminants from worldwide populations. In each case, we called a majority rule consensus for sites with depth of coverage ≥5X, using reads with mapping quality ≥30 and bases with quality ≥20. We ran *contamMix* with 100,000 iterations, where the first 10,000 were removed. Convergence can be inspected visually from the variation of the proportion of authentic DNA over time and/or from the potential scale reduction factors. We considered that we reached convergence in the estimation of the proportion of authentic DNA for upper limits of the potential scale reduction factors below 1.10. We report mtDNA contamination estimates for each library and for the full dataset for each individual in Table S1.

S3.4.3.2. 'Ancient Rapanui' mitochondrial DNA

Using the mtDNA consensus sequences that we called for estimating mitochondrial contamination (Section S3.4.3.1), we assigned a mitochondrial haplogroup for each individual using *HaploGrep2* [33]. Haplogroup assignments are reported in Table S1. Out of the 15 'Ancient Rapanui', 14 carry haplogroups derived from B4a1a and one individual carry haplogroup Q1f1. Both haplogroups have been previously reported in Eastern Polynesian

peoples [34–36]. In particular, these assignments confirm the Polynesian origin of the 15 ancient individuals and are in line with the only two previous genetic studies of ancient Rapanui individuals [37,38].

S3.4.3.3. X-chromosome contamination inference

We used *contaminationX* [39] to estimate nuclear contamination for XY individuals (Section S3.3). Briefly, this method relies on the fact that for XY individuals, where the X chromosome is haploid, observing more than one allele at a given site can be attributed to error or contamination. For all libraries and individuals with depth of coverage ≥0.5X, we used reads with a mapping quality ≥30, bases with quality ≥ 20, sites with depth between 3 and 20, the HapMap CEU allele frequencies [40] and the 'two-consensus model' to estimate X-chromosome contamination. Contamination estimates range between 0.63% and 2.00% and are reported in Table S1.

S3.4.4. 'Ancient Rapanui' Y-chromosome haplogroups

We used *pathPhynder* [41] and its associated ISOGG Y-chromosome reference data (last curated 13 May, 2021) to place the XY 'Ancient Rapanui 'individuals into a phylogeny including a worldwide set of Y-chromosomes. Since a number of individuals are represented by low-depth data, this strategy allowed us to prevent potential miscalls (caused by missing diagnostic SNPs) by callers designed for low-missingness present-day data. We ran *pathPhynder* restricting to transversion polymorphisms and set the remaining parameters to their default values. *pathPhynder* haplogroup assignments are reported in Table S1. The eight XY individuals were placed together with individuals carrying haplogroup C1b2a1c. Haplogroup C1b is most widely present in East Asia and Oceania [36,42,43]. Thus, these assignments, together with the mitochondrial DNA haplogroups and the genomic ancestry analyses (see below) support the Polynesian origin of the 15 ancient individuals. Although we only detect Polynesian (and no Native American) mtDNA and Y-chromosome haplogroups in the 'Ancient Rapanui', we consider these results are within statistical noise based on the low Native American admixture proportion ~10% (Section S13,14). We anticipate that as more ancient genomes from the region are sequenced, a fuller picture of the Polynesian uniparental genetic pool will become available.

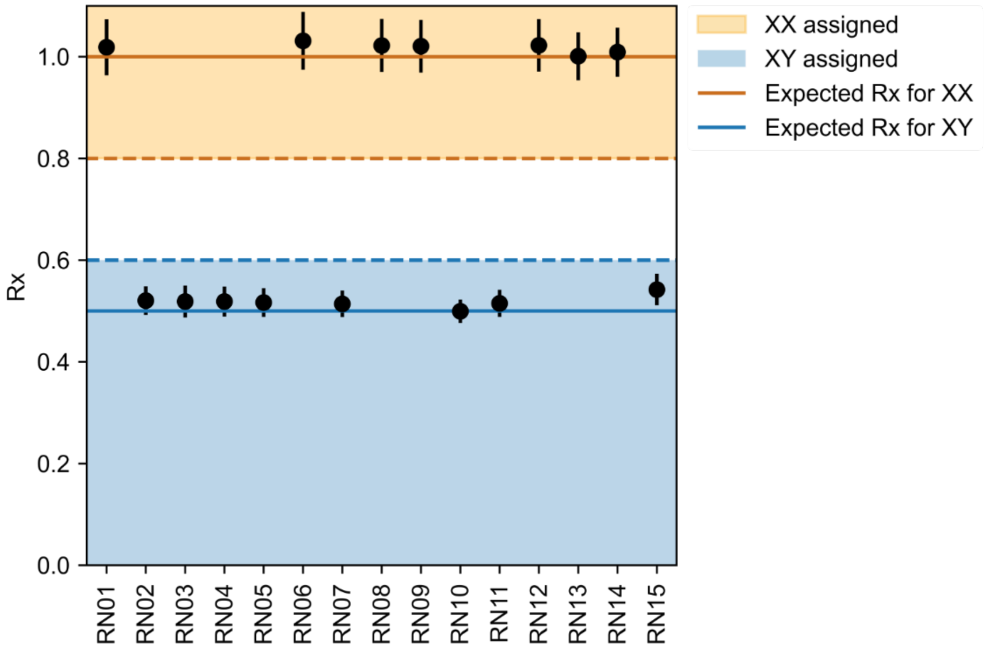

Figure S2. Chromosomal sex determination for the 15 'Ancient Rapanui'. We estimated the ratio of the average depth of coverage for X chromosome and the autosomal depth of coverage, Rx. The error bars correspond to the 1.96 times the standard error of Rx (computed using the estimates for the 22 autosomes). Individuals were assigned as XX if Rx>0.80 and as XY if Rx<0.60.

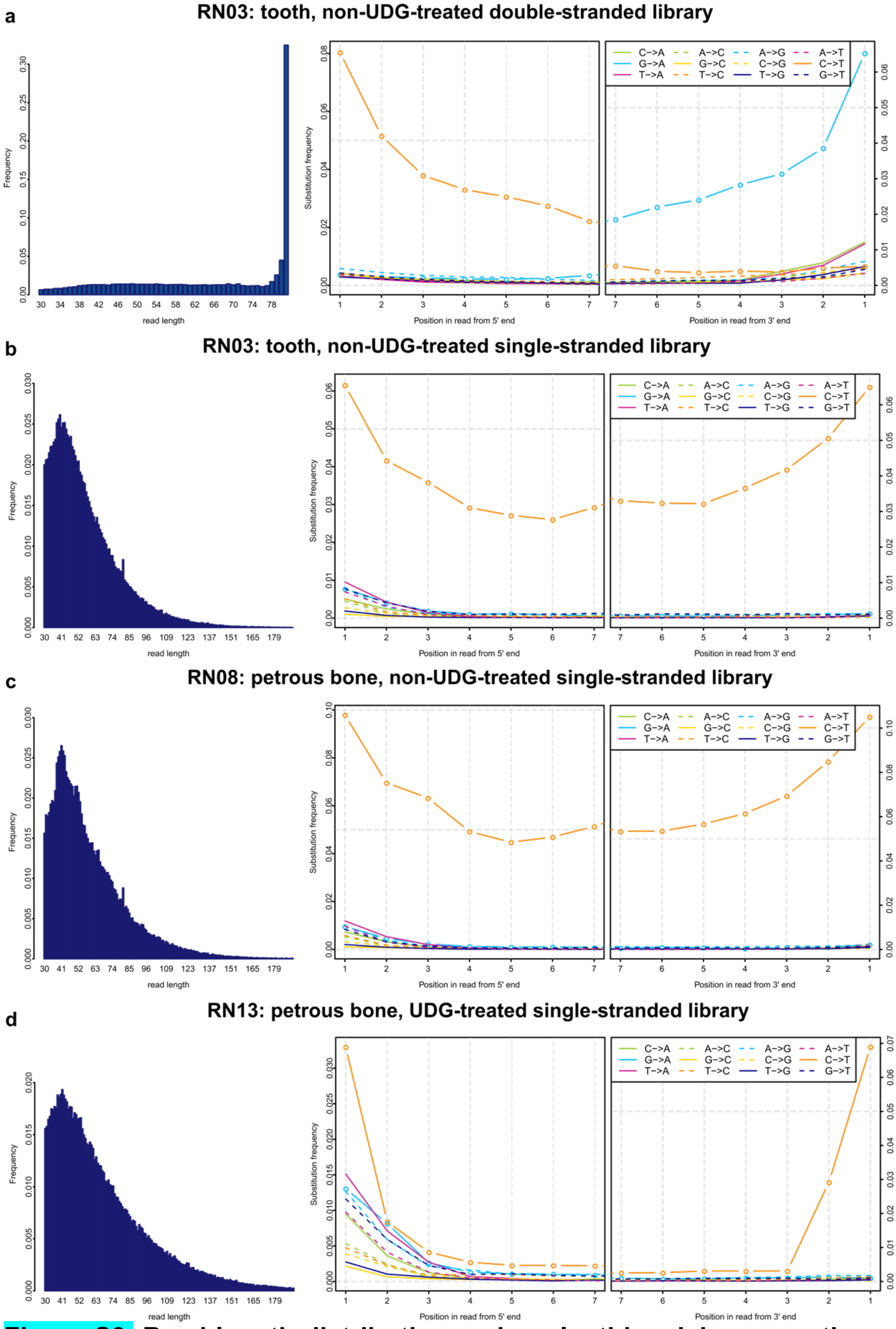

Figure S3. Read length distribution and nucleotide misincorporation patterns at the ends of the reads for three representative libraries. a. Double-stranded library built from bone powder extracted from tooth (individual sample RN03). **b.** Single-stranded library built from bone powder extracted from tooth (individual sample RN03). **c.** Single-stranded library built from bone powder extracted from petrous bone (individual sample RN08). **d.** Single-stranded library built from a USER-treated extract from individual RN13.

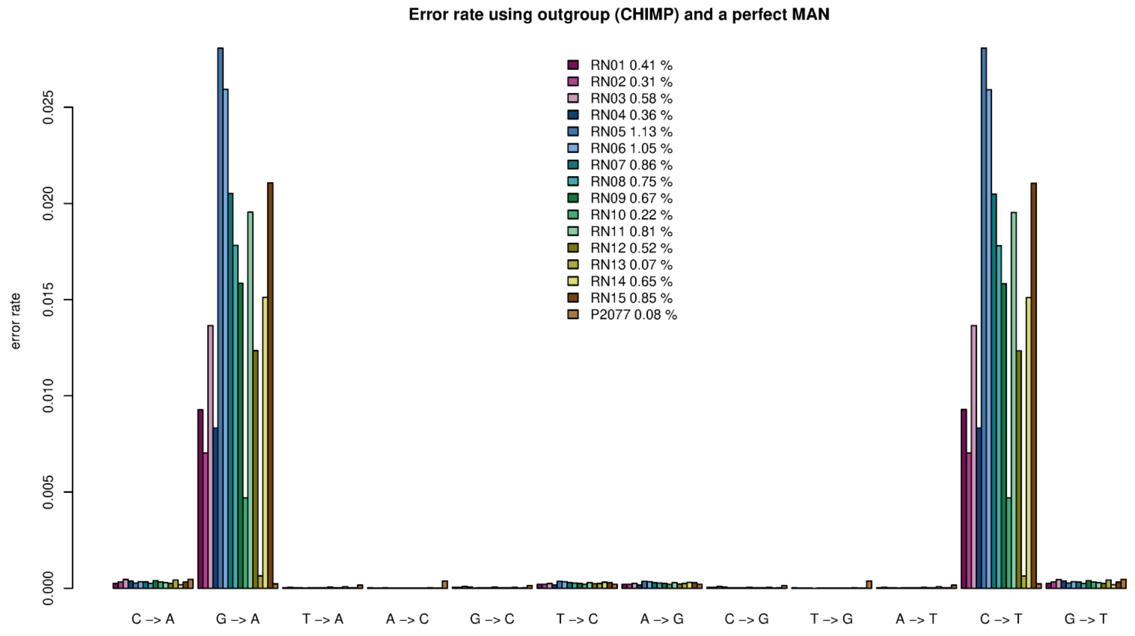

Figure S4. Error rates estimates of ancient genomes and P2077, a high-coverage present-day Rapanui genome (59x). The *x*-axis specifies mismatch types with corresponding error rates on the *y*-axis. For each individual, the overall error rates are specified at the plot's label. Reads from ancient individuals were previously trimmed (5 bp and 2 bp for non-USER and USER-treated DNA, respectively).

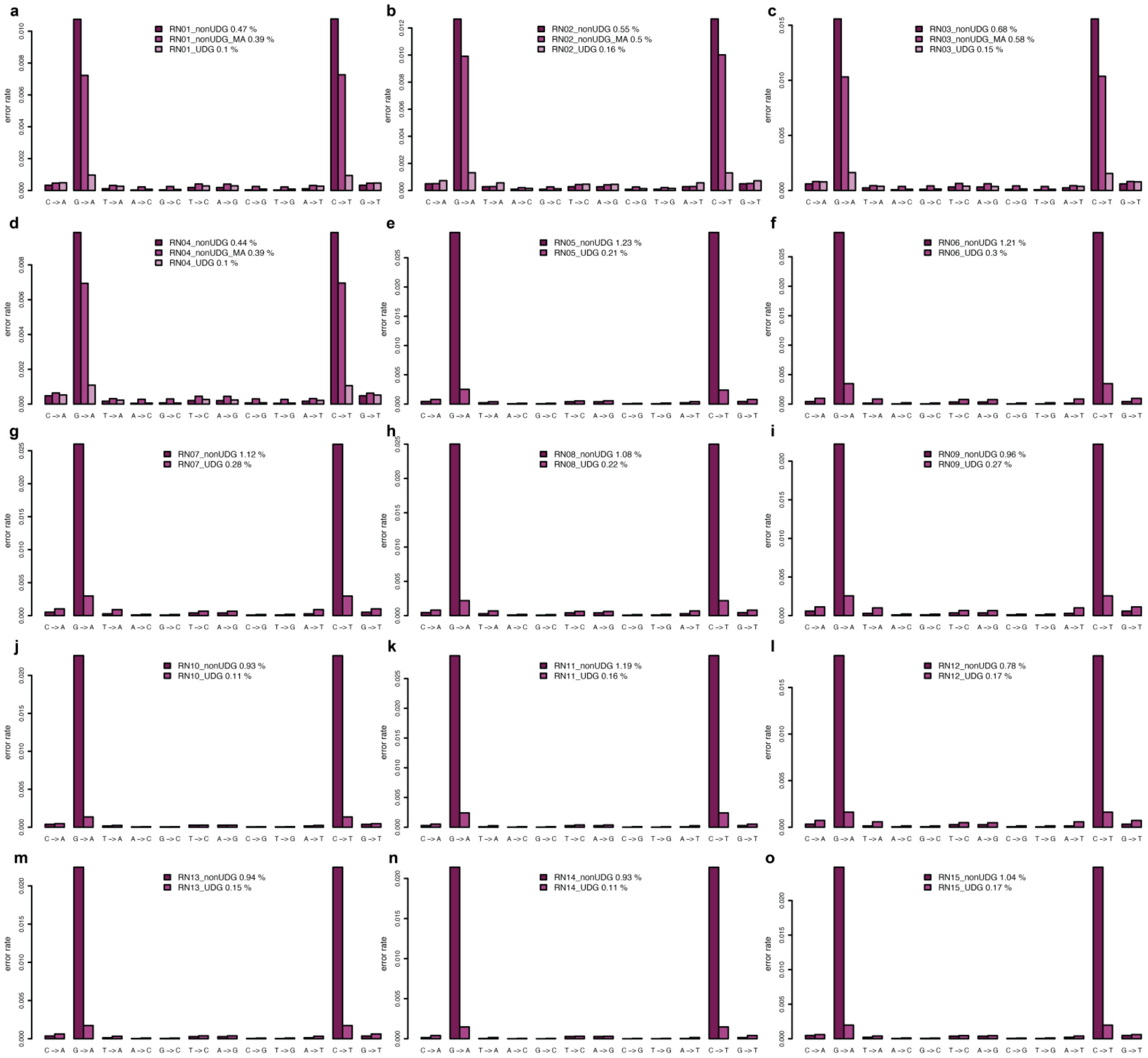

Figure S5. Error rates per sequenced library. a-o. Error rates estimated per library per individual sample (RN01-RN15).

S4. Reference data

S4.1. SNP array reference dataset

For MDS (Section S6), *f*-statistics (Section S7), *ADMIXTURE* (Section S12), local ancestry inference (Section S13) and admixture dating (Section S14), we considered SNP array data from [44–46]. These include 574 individuals from 30 worldwide populations genotyped using the Affymetrix Human SNP Array 6.0 across 755,094 autosomal SNP sites. In particular, this dataset includes individuals from the Polynesian islands of Tuvalu, Futuna, Tokelau, Tonga, Samoa, Niue, and the Cook Islands. Furthermore, this dataset includes Native American individuals [45] (Totonac and Bolivian) that do not carry European or African admixture [46].

S4.2. 'Ancient Polynesians' from an unknown sampling location

In all analyses, we included whole-genome sequencing data from two individuals with Polynesian genetic ancestry whose exact origin is however unknown [47]. We refer to these individuals as 'Ancient Polynesian' individuals from an unknown location. We use these data as a 'control' for ancient Polynesian genomes without Native American or European admixture. Similar to the 'Ancient Rapanui' individuals, we conduct all analyses with two call types for these two ancient individuals: pseudo-haploid calls and imputed diploid calls (Section S5).

S4.3. SGDP

For *f*-statistics (Section S7) and local ancestry inference (Section S13) analyses, we confirm our SNP array-based results using the whole-genome data from the Simons Genome Diversity Project (SGDP) [30]. We downloaded the publicly available, filtered SGDP v4 callset from https://sharehost.hms.harvard.edu/genetics/reich_lab/sgdp/variant_set/. For all analyses involving this dataset, we excluded non-autosomal markers and filtered out SNP sites with a minor allele frequency <0.05. After filtering, the dataset contains 345 individuals from 164 populations, genotyped over 5,494,521 autosomal SNP sites. In addition, we made use of bam files containing the aligned DNA sequences for a subset of the SGDP individual samples in the imputation experiments (Section S5) and ROH analysis (Section S10).

S4.4. Native American reference dataset

To investigate the source of the Native American ancestry in the 'Ancient Rapanui', we compiled a reference dataset including ancient and present-day individuals carrying 'Native American ancestry' exclusively. To maximise the number of individuals and populations, we included whole-genome sequencing data and SNP capture data, and restricted the analyses to sites included in the '1240k' SNP capture set [48]. The reference panel contains all ancient and present-day Native Americans compiled in [49] ([18,19,30,50–56]), supplemented with data from [57–60]. For a few high-depth individuals, we considered the diploid genotypes called in [49], and for the rest, we called pseudo-haploid genotypes.

S4.5. High-coverage 1000G reference panel

To perform imputation (Section S5), we used the same reference panel as in [61]: re-sequenced 1000 Genomes v5 phase 3 (2504 genomes) to high coverage [62], phased with TOPMed [63]. These data can be accessed in European Nucleotide Archive, under project PRJEB31736 and secondary study accession ERP114329. The reference panel was lifted over from hg38 to hg19 reference genome assembly with Picard liftoverVCF v1.18.11 (https://gatk.broadinstitute.org/hc/en-us/articles/360037060932-LiftoverVcf-Picard), with the hg38ToHg19 chain file from the University of California, Santa Cruz liftOver tool (http://hgdownload.cse.ucsc.edu/goldenpath/hg38/liftOver/).

S4.6. Previously published low-coverage ancient Rapanui genomes

We ran *D*-statistics (Section S7) and genetic clustering analyses (*ADMIXTURE*, Section S12) on five previously published ancient Rapanui genomes [37]. These genomes have very low depth of coverage (0.0004x-0.004x), and three of these (RN035, RN036 and RN041) were dated to before European contact in 1722 CE, while the remaining postdate European contact (RN037 and RN039). The original publication reported that none of these individuals had Native American genetic ancestry, unlike present-day Rapanui. Moreover, one of the most recent genomes (RN039) was found to bear 20% of European ancestry.

S5. Imputation accuracy of 'Ancient Rapanui' genomes

Most standard reference panels used in imputation do not have genomes of Polynesian or Papuan origin. That is also the case of the 1000 Genomes data set. Therefore, it was not clear whether we could accurately impute the 'Ancient Rapanui' genomes we study here, using the 1000 Genomes data set as a reference panel. To address this question, we proceeded as in [61]. We imputed downsampled versions of high-coverage ancient genomes from two 'Ancient Rapanui' individual sequenced in this study, RN13 (17x) and RN14 (26x), and compared the imputed diploid genotypes to called diploid genotypes from the full dataset, which we used as the ground truth. Furthermore, we conducted a similar experiment on present-day genomes whose ancestry is related to Polynesian ancestry. That is, we imputed five 1X downsampled present-day genomes from Oceania and China to better assess imputation accuracy for genomes with Polynesian and Papuan ancestry.

5.1. Methods

S5.1.1. Present-day genomes data set

We assessed imputation accuracy for Polynesian genomes using a high-coverage present-day Rapanui genome (P2077, 59X) [28] and four genomes from the SGDP data [30] : two Polynesians (LP6005592-DNA_H03, 46X (Hawaii) and LP6005592-DNA_B02, 37X (Maori)), a Papuan (LP6005441-DNA_B10, 45X) and a Han Chinese (SS6004469, 38X).

S5.1.2. Validation data set

To generate the validation data set from the high-coverage genomes, we called genotypes and filtered the data in the same way as in [49]: (i) we called genotypes using *bcftools* v1.12 [23] and parameter *–C 50* from reads with mapping quality of at least 30 and bases with quality of at least 20; (ii) we only kept sites present in the 1000 Genomes accessible strict mask [62]; (iii) we removed sites present in the UCSC repeat regions (http://genome.ucsc.edu/ [64]); (iv) we also removed sites with extreme depths when compared to the average depth of coverage (DoC), that is, we kept sites satisfying max(8,DoC/3) ≤ DoC ≤ 2DoC; (v) we kept sites whose vcf QUAL field was at least 30.

S5.1.3. Imputation

We downsampled the genomes using *samtools* v1.12 [23] to coverages of 1.0x in the case of the present-day genomes, and in the range 0.1x-10.0x in the case of RN14, thus encompassing the depths of coverage (DoC) of all the low and medium-coverage 'Ancient Rapanui' genomes (DoC≤10x). Then, we generated genotype likelihoods with *bcftools* v1.12 [23], the input data for imputation. We used *GLIMPSE* v1.1.1 [65] to impute the data. We started by breaking the chromosomes with *GLIMPSE_chunk* in chunks with sizes between 1 and 2 Mb. These chunks contained a 200-kb buffer region on each end to diminish border effects. We then imputed these chunks with *GLIMPSE_phase* (*- - burn 10*, *- - main 15*, *- - pbwt-depth 2*). Finally, we ligated the imputed chunks using *GLIMPSE_ligate*.

S5.1.4. Assessing imputation accuracy and genotype concordance

We ran *GLIMPSE_concordance* to measure the level of agreement between imputed and high-coverage genomes. *GLIMPSE_concordance* outputs genotype discordance across sites with different copy numbers of the reference allele, as well as the non-reference discordance (NRD) values, and imputation accuracy, that is, the Pearson correlation, r^2, between imputed genotype dosages and validation genotypes as a function of minor allele frequency (MAF) as defined by the 1000 Genomes reference panel. Only sites in the17x RN13 and 26X RN14 genomes covered by at least eight reads and with a posterior probability equal or greater than 0.9999 were used to validate imputation.

S5.1.4. D-statistics for testing imputation bias towards the imputation reference panel

We used *D*-statistics to assess whether imputation introduced a bias towards the imputation reference panel, *i.e.*, 1000 Genomes, in the 'Ancient Rapanui' imputed genotypes. As above, we relied on high-coverage genomes that we downsampled to 1x and imputed. We computed *D*-statistics following the methods detailed in Section S7.1 for P2077, RN13 and RN14. The imputed genotypes were filtered for minor allele frequency (MAF>1%) and genotype probability (GP>0.99) and only biallelic SNPs with MAF>0.5% in the 1000 Genomes panel were kept. For all tests involving an ancient genome, we removed excluded transition SNPs.

S5.2. Results

S5.2.1. Full 1000G panel

S5.2.1.1. Exploring the effect of depth of coverage on the imputation of an 'Ancient Rapanui' genome

When downsampling RN14 to various coverages, imputed genotypes were closer to the validation calls as the genome coverage increased, which is shown by both imputation accuracy as a function of MAF and genotype discordance (Figure S6). Common variants (MAF≥5%) were more accurately imputed, for which imputation accuracy was at least 0.54 (0.1X) and was above 0.90 for depth ≥0.75X. Genotyping errors were below 5% for sites with at least one copy of the alternative allele starting at a depth of coverage of 1.0X. These errors reached 26% in the case of the imputed 0.1x genome. NRD was 6.4% for 1X data and below 5% for the genomes with higher coverages.

S5.2.1.2. Exploring the effect of genomic ancestry on the imputation accuracy

Fixing depth at 1X, we compared imputation accuracy between the present-day genomes, RN13 and RN14 (Figure S6). Imputation of the Han and the Maori genomes resulted in the most accurate genotypes, starting at 74% for the lowest MAF bin (0.1-1%) and reaching around 96% at common variants (MAF≥5%). The Papuan genome revealed to be the most difficult to impute with NRD values of about 10%. These differences in imputation accuracy can be explained by reference panel representation. The Han population is represented in 1000 Genomes data set, whereas Papuan ancestry harbouring unique genetic variation is not [28,30,66]. The r^2 curves for the two other modern Polynesians, LP6005592-DNA_H03 (Hawaii) and P2077 (Rapa Nui), fall in between these two extremes, but closer to the most

accurately imputed one. However, we must be cautious about making conclusions regarding imputation of Polynesian genomes from the imputed Maori genome alone, since this individual seems to be admixed [30]. Imputation of the 1X downsampled RN14 genome yielded the second least accurate results. However, imputation of 1x RN13 produced genotypes that were as accurate as those for the 1x present-day Rapanui genome.

S5.2.1.3. Evaluating *post-mortem* damage impact on imputation experiments

Differences in imputation accuracy between ancient and present-day genomes from the same population may be attributed to ancient genomes containing more errors coming from sequencing, post-mortem damage and contamination that can have some impact on imputation and on the validation data. We investigate the impact of post-mortem damage on our results using two downsampled (1x) high-depth ancient Rapanui genomes, RN13 and RN14. RN14 contains both non-UDG and UDG-treated libraries, which leads to a substantial increase of transition SNPs being affected by post-mortem damage, whereas the RN13 genome is comprised of UDG-treated libraries only. This difference is also reflected in their respective error rates (RN14: 0.65%; RN13: 0.07%, Figure S4). As a control, we conduct the same experiments on the imputed 1x P2077, a present-day Rapanui genome, that has a similar imputation accuracy as RN13.

When assessing imputation performance at SNPs affected by deamination (C/T and T/C), we found no considerable decrease in accuracy compared to non-C-T sites (FigureS7a,b,c), and similar differences were found in the present-day genome. Moreover, such decrease was smaller than the difference between the imputation accuracies of RN13 and RN14. We further estimated ROH using all SNPs in the high-coverage 1000G dataset (Section S4.5) (MAF>1%) and restricting to transversion SNPs. We found that, for the same SNP subsets, the total ROH lengths in the imputed and high-depth genomes were similar (Figure S7d). Furthermore, when we focused on different ROH size categories, we found similar total lengths, except for RN14 when using all sites. For RN14, ROH≥12 cM is absent from the validation dataset (called diploid genotypes) when considering transitions in contrast to the ROH estimated using imputed genotypes. However, this difference between the validation and imputed datasets disappears when we restrict the analysis to transversions.

To exemplify our observations, we use chromosome 10 (Figure S7e), where we observed ROH that were either absent or were shorter in the high-depth RN14 (all sites) while there was a complete overlap between the two imputed (transversions only and all sites) and the high-depth transversion datasets. We interpret that the larger amount of deaminated SNPs in RN14 may have led to an excess of heterozygous sites that break ROH in the validation dataset. While this analysis is inconclusive regarding the impact of post-mortem damage on imputation, it shows, on the one hand, how challenging it is to obtain a true validation dataset for damaged ancient genomes even when high-depth data is available and, on the other hand, that imputation can mitigate the effect of deamination, as shown in [61].

S5.2.1.4. Investigating whether imputation introduces a bias towards the imputation reference panel

To investigate the bias introduced by imputation (see also [61] for extensive work on the topic), we determined whether the affinity of the imputed 'Ancient Rapanui' genomes to the populations included in the imputation reference panel is increased. To do so, we estimated *D*-statistics of the form *D(high-coverage genome X, imputed 1x genome X; reference population, Yoruba)*, where genome *X* represents either RN13 or RN14 (see method details in Section S7). To produce a more accurate ground truth using the non-imputed high-coverage genomes (called diploid genotypes), which are affected by C-to-T damage, we restricted this analysis to transversion sites. We observed that the reference populations that yielded the most negative *D* values, *i.e.*, being closer to the imputed genomes, tended to be of East Asian or American origin (*e.g.*, Han Chinese in Beijing (CHB), Southern Han Chinese (CHS), Japanese in Tokyo (JPT) and Peruvian in Lima (PEL)), while we found European populations on the other extreme. This ordering might reflect the ancestry of the reference haplotypes which the imputation algorithm copied from, as the 'Ancient Rapanui' are expected to be more closely related to East Asian individuals and they carry ~10% of Native American ancestry. However, for the two 'Ancient Rapanui' genomes and all reference populations, the *D*-statistics 99%-confidence interval included 0, which supports the idea that none of the imputed genomes had significantly higher (or lower) affinity to any of the reference populations when compared to their high-coverage counterpart (Figure S8, Table S3). Importantly, the magnitude of the *D*-statistics is extremely small, *i.e.*, in the order of 10^{-4}, 100 times smaller than the largest *D*-statistics absolute values obtained when we test for Native American admixture, e.g., Figure S13. This difference suggests that there is only a small number of SNPs that can be used for *D*-statistics tests due to the high genotype concordance between the imputed and called diploid genotypes, even when the same populations that were used for imputation (1000 Genomes) are included as a test population. Based on these results, we conclude that imputation did not produce a significant bias towards the imputation reference panel.

S5.2.1.5. Post-imputation filtering

For downstream analyses using the imputed data, we excluded sites whose genotype probability (GP) is below a certain threshold. This allows to exclude sites that are not confidently imputed. We explore here several values for this threshold to find a balance between higher accuracy without removing too many correct heterozygous calls.

To do so, we quantified imputation accuracy for imputed downsampled genomes with 0.5X, 1.0X and 2.0X depth of coverage after filtering for GP using different thresholds and determined the proportion of remaining correct heterozygous sites. For the lowest MAF bin (0.1-1%), there is an increase in imputation accuracy of about 0.2 for all three depths, but, as the coverage increased, there was a smaller gap in imputation accuracy of more common variants (MAF>2%). For the three depths, imputation accuracy was very close to 1 when MAF≥10%. As expected, the imputed 0.5X genome lost the highest proportion of correctly imputed heterozygous sites as we applied stricter GP filtering, losing between 3.8% (GP≥0.70) and 23% (GP≥>0.99). In contrast, the imputed 1.0X and 2.0X genomes lost at most 13% and 7% of correctly imputed heterozygous sites, respectively (Figure S9).

Based on these results, for downstream population genetics analyses, we required the imputed diploid genotypes for ancient individuals to have a GP>0.99 and excluded sites with a MAF<1% in the reference panel. With this filtering approach, the 1.0x 'Ancient Rapanui' genome was imputed with r^2>0.90 (Figure S9), *i.e.*, yielding a higher imputation accuracy than the unfiltered imputed 1.0x present-day genomes. Therefore, we consider the imputed diploid genotypes are reliable for the analyses we conduct below. Furthermore, we note results based on imputed diploid genotypes recapitulate results based on pseudo-haploid calls (see, for example, MDS (Section S6), *f*-statistics (Section S7) and ALD-based dating (Section S14)).

S5.2.2. Benchmark imputation without Peruvians

To rule out that the identification of the Native American source population (Section S7) was not biased by imputation, we imputed downsampled (0.1x-8.0x) versions of the RN14 genome using a subset of the reference panel without the Peruvian genetic data (labelled as PEL). We then assessed imputation accuracy using the modified reference panel and compared with the previous results. We found that removing the PEL genetic data did not reduce imputation accuracy (Figure S10). Moreover, we obtained consistent results when we repeated *f*-statistics analyses (Section S7) and local ancestry inference (Section S13) using the modified imputation reference panel.

Our comparisons between imputed and called diploid genotypes suggest that the filtered imputed diploid genotypes are accurate.

However, as imputation could in principle bias some of the population genetic results, for MDS (Section S6), *f*-statistics (Section S7) and relatedness (Section S9) analyses, we consider two different call types for the ancient individuals, *i.e.*, pseudo-haploid calls and imputed diploid genotypes. Furthermore, for admixture characterisation analyses that rely on diploid genotypes (*f*-statistics (Section S7.5) and local ancestry inference (Section S13)) we confirm our results using a modified imputation reference panel (Section S5.2.2.). In addition to our accuracy survey in this section, that we obtain comparable results across call types and imputation runs supports that the imputed diploid genotypes for the 'Ancient Rapanui' individuals are suitable for downstream population genetics analyses.

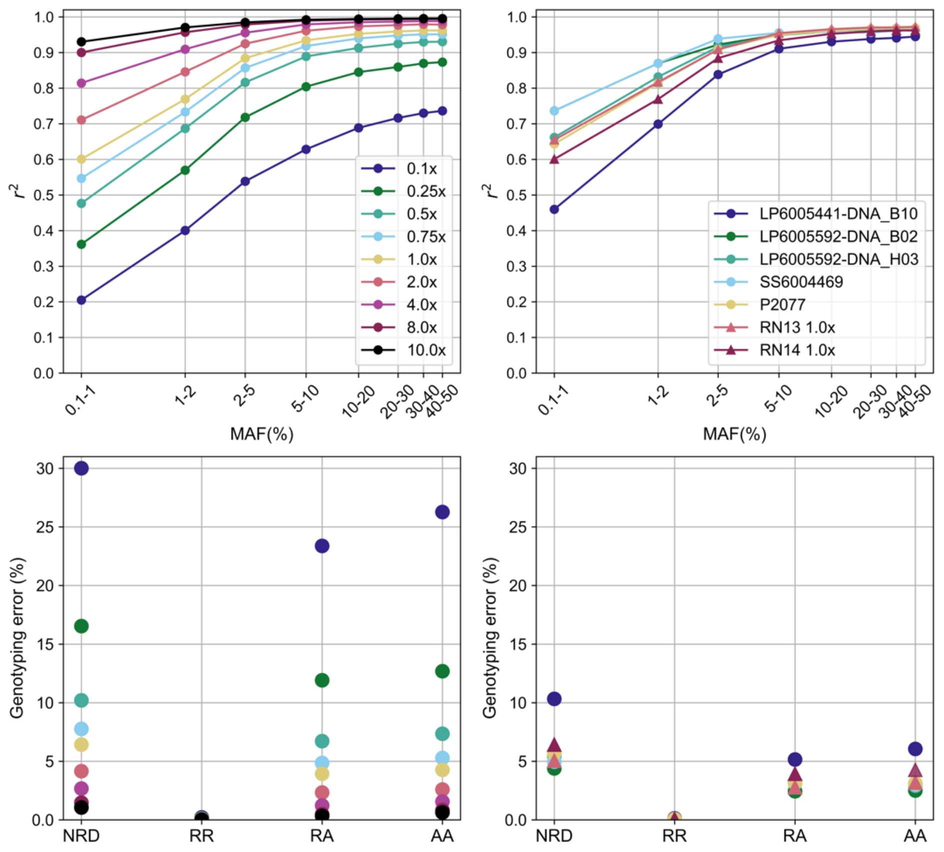

Figure S6. Imputation performance assessment of an 'Ancient Rapanui' genome (RN14) and five present-day Oceanian and Han genomes. Top: imputation accuracy, r^2, as a function of minor allele frequency (MAF); bottom: genotyping errors produced by imputation at homozygous reference allele (RR), heterozygous (RA) and homozygous alternative allele (AA) sites, and the resulting non-reference discordance value (NRD). Left: imputation performance for RN14 across different depths of coverages; right: imputation performance for the five present-day genomes and RN14 downsampled at 1x. Individual IDs: Papuan (LP6005441-DNA_B10, 45X), Maori (LP6005592-DNA_B02, 37X), Hawaii (LP6005592-DNA_H03, 46X) (Hawaii), present-day Rapanui (P2077 59X), Han Chinese (SS6004469, 38X), 'Ancient Rapanui' (RN14, 26X).

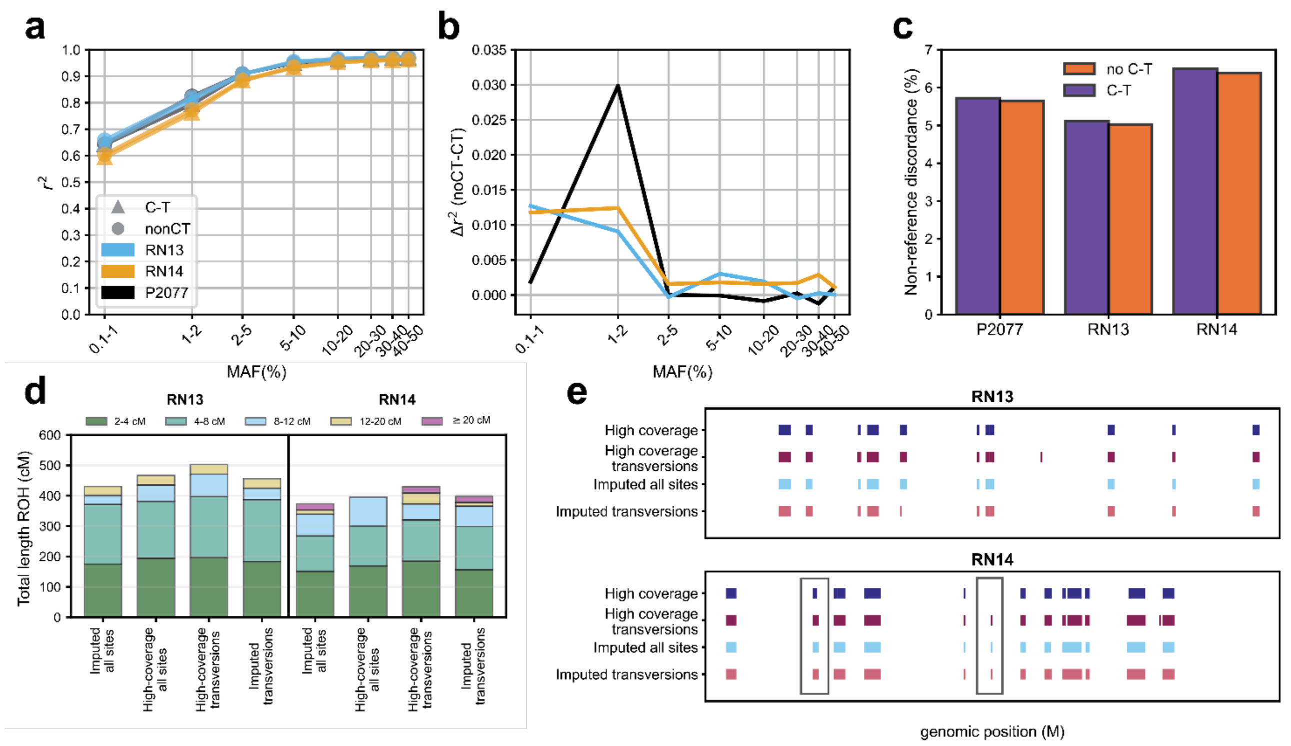

Figure S7. Imputation accuracy at potentially deaminated sites. We compared imputation performance at C/T and T/C (C<->T) polymorphisms with the remaining SNPs when imputing downsampled to 1x Rapanui genomes: P2077, present-day genome, and RN13 and RN14, two high-depth 'Ancient Rapanui' genomes sequenced in this study (**a**, **b** and **c**). RN13's sequences are the result of UDG-treated libraries, whereas RN14 is the result of a mixture of UDG with non-UDG-treated libraries (Table S1). We also evaluated the impact of deamination in runs of homozygosity (ROH) detection for RN13 and RN14, using either imputed (no downsampling) or the high-depth data at all sites or at transversions only (**d** and **e**). **a.** Squared Pearson correlation, r^2, between imputed dosages and high-depth genotypes as a function of minor allele frequency (MAF) when restricting to C<->T sites (triangles) and to the remaining sites (filled circles) when imputing 1x RN13, 1x RN14 and 1x P2077. **b.** Difference in r^2 between non-C<->T as a function of MAF for the curves in **a**. **c.** Non-reference discordance (NRD) per sample and for C<->T (purple) and non-C<->T (orange) SNPs. **d.** Total length of inferred ROH in RN13 and RN14 discriminated by ROH length for imputed and high-depth data at all SNPs vs. transversion polymorphisms. **e**. ROH segments detected in chromosome 10 comparing the abovementioned combinations of SNPs and data. We highlighted two regions in RN14 where either the ROH was absent or was shorter in the high-depth genome while being consistent across the remaining three combinations.

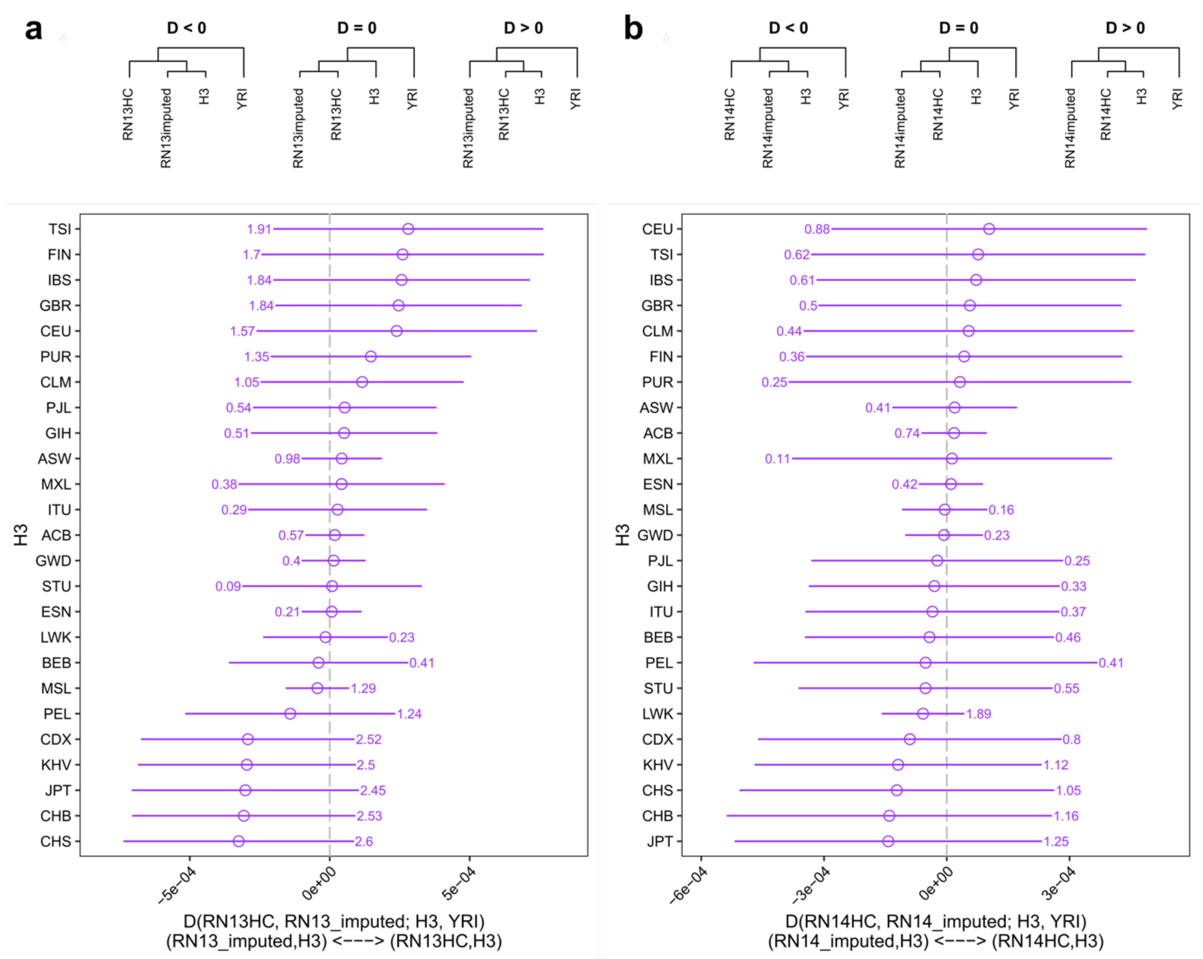

Figure S8. *D*-statistics testing for potential imputation bias towards reference panel populations. We computed *D*-statistics of the form *D(high-coverage genome, imputed 1x genome; 1000G population, Yoruba)* for two 'Ancient Rapanui' genomes: **a.** RN13 (ancient Rapanui genome at 17x) and **b.** RN14 (ancient Rapanui genome at 26x). We kept only transversion polymorphisms with a minor allele frequency (MAF) above 0.5%, and the imputed genotypes had been filtered for MAF>1% and GP>0.99. Reference populations in 1000 Genomes panel: ACB: African Caribbean in Barbados, ASW: African ancestry in Southwest USA, BEB: Bengali from Bangladesh, CDX: Chinese Dai in Xishuangbanna, China, CEU: Utah residents with Northern and Western European ancestry, CHB: Han Chinese in Beijing, China, CHS: Southern Han Chinese, CLM: Colombian in Medellin, Colombia, ESN: Esan in Nigeria, FIN: Finnish in Finland, GBR: British in England and Scotland, GIH: Gujarati Indian from Houston, Texas, GWD: Gambian in Western Divisions in the Gambia, IBS: Iberian populations in Spain, ITU: Indian Telugu from the UK, JPT: Japanese in Tokyo, Japan, KHV: Kinh in Ho Chi Minh City, Vietnam, LWK: Luhya in Webuye, Kenya, MSL: Mende in Sierra Leone, MXL: Mexican ancestry in Los Angeles, California, PEL: Peruvian in Lima, Peru, PJL: Punjabi from Lahore, Pakistan, PUR: Puerto Rican in Puerto Rico, STU: Sri Lankan Tamil from the UK, TSI: Toscani in Italy, YRI: Yoruba in Ibadan, Nigeria. Points represent *D*-statistics, and error bars represent ~3.3 SEs (*p*-value of ~0.001 in a *Z* test). Numbers next to each point indicate the *Z*-score for each test. Raw results are reported in Table S3.

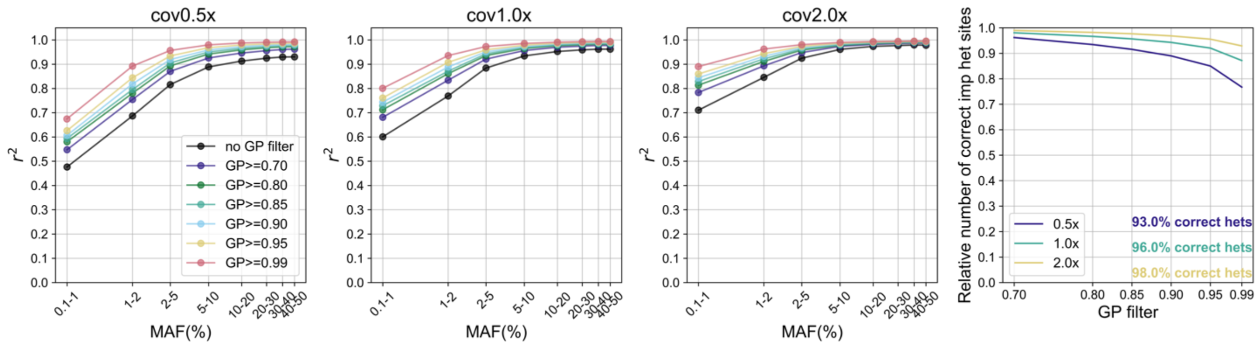

Figure S9. Effect of filtering imputed data for genotype probability (GP) on imputation accuracy and number of correctly imputed heterozygous sites. From left to right: the first three plots compare imputation accuracy as a function of minor allele frequency (MAF) when different GP thresholds are used for filtering for three different depths of coverage (0.5x, 1.0x and 2.0x); the last plot shows the relative number of retained correctly imputed heterozygous sites when progressively higher GP filters were applied (no GP filter corresponds to a relative number of correctly imputed heterozygous sites equal to 1.0). The numbers on the right lower corner of the rightmost plot correspond to the percentage of correctly imputed heterozygous sites before filtering for GP for the three different downsampling genome coverages.

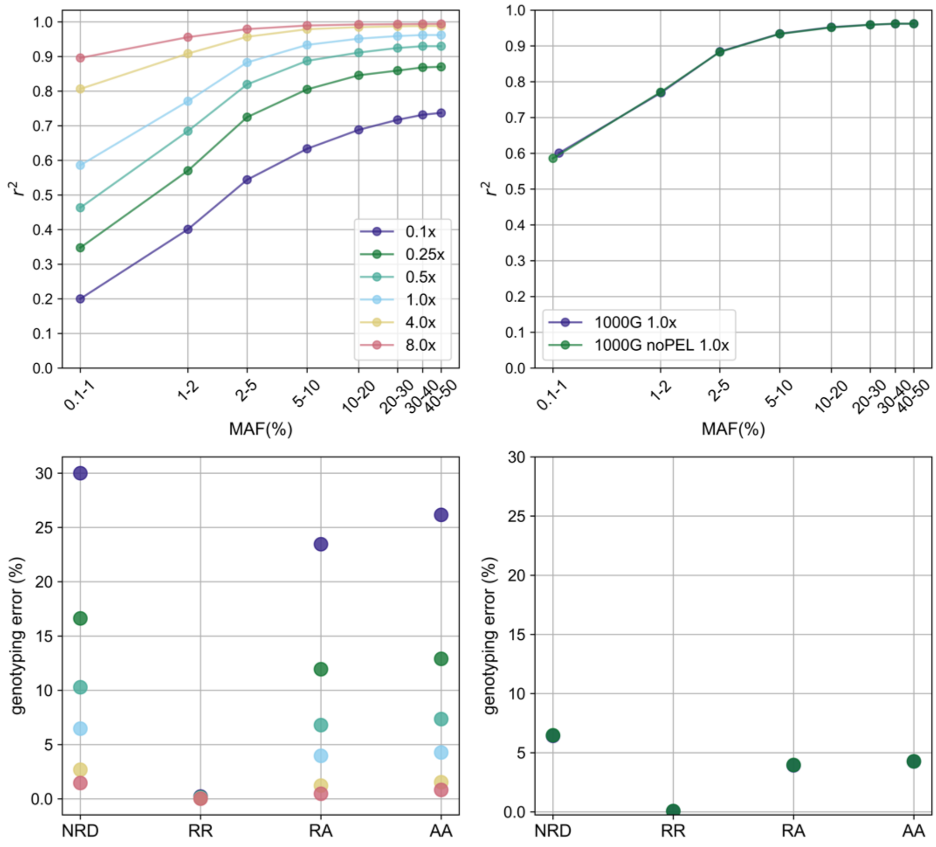

Figure S10. Imputation performance assessment of an 'Ancient Rapanui' genome (RN14) when removing Peruvian (labelled as PEL) genotypes from the imputation reference panel. Top: imputation accuracy, r^2, as a function of minor allele frequency (MAF); bottom: genotyping errors produced by imputation at homozygous reference allele (RR), heterozygous (RA) and homozygous alternative allele (AA) sites, and the resulting non-reference discordance value (NRD). Left: comparing imputation performance when removing Peruvian genotypes (blue) with using the full reference panel (red) for RN14 downsampled to 1x.

S6. Multidimensional scaling

S6.1. Methods

We used multidimensional scaling (MDS) [26] to explore the broad genetic affinities between the ancient individuals sequenced in this study and a worldwide SNP array reference panel enriched in Oceanian populations (Section S4.1). We computed the pairwise identity-by-state distance matrix between all individuals using *plink* [67] and used the R *cmdscale* function to obtain MDS transformations for different subsets of the data, across different MDS dimensions. We carried out this analysis using two versions of the dataset to explore potential differences between imputed diploid genotypes and pseudo-haploid calls. First, we considered a dataset where all ancient individuals were included as pseudo-haploid calls and all diploid genotype calls for present-day individuals were turned into random pseudo-haploids. Second, we considered a dataset including imputed diploid genotypes for the ancient individuals (Section S5) and the original diploid calls for present-day individuals.

S6.2. Ancient individuals are most closely related to present-day Rapanui

When we considered the full dataset including individuals from all broad continental ancestries, the ancient individuals were placed within Polynesian genomic diversity across the first two MDS dimensions (Figure S11a,d). To explore whether the ancient individuals sequenced in this study were more closely related to a specific group, we plotted higher MDS dimensions. Along dimension 5, which separated Native American individuals, present-day Rapanui were shifted towards Native Americans, in agreement with admixture reported in [46,68]. Notably, the 15 ancient individuals were placed together with the present-day Rapanui individuals without European admixture (Figure S11b,e).

We computed a second MDS transformation on a subset of the data including only Polynesian individuals. In this case, the 15 ancient individuals sequenced in this study and other Polynesian individuals were placed at opposite ends of dimension 1 (Figure S11c,f). Meanwhile a Cook Islander and the two 'Ancient Polynesian' individuals were placed in an intermediate position, likely representing an east-west cline. Present-day Rapanui were placed together with the 15 ancient individuals along dimension 1, with the exception of two individuals that bear >40% European admixture [46].

These results support that the 15 ancient individuals sequenced in this study are Polynesian in origin and they are most closely related to present-day Rapanui individuals. Furthermore, they suggest that 'Ancient Rapanui', like present-day individuals from the island bear Native American admixture. Yet, the 'Ancient Rapanui' were not shifted towards individuals with western Eurasian ancestry, suggesting they do not carry European admixture (see also Section S7), which has been estimated to enter the population more recently [46,68]. We note these results were consistent for both call types for the ancient individuals, *i.e.*, pseudo-haploid calls (Figure S11a,b,c) and imputed diploid genotypes (Figure S11d,e,f).

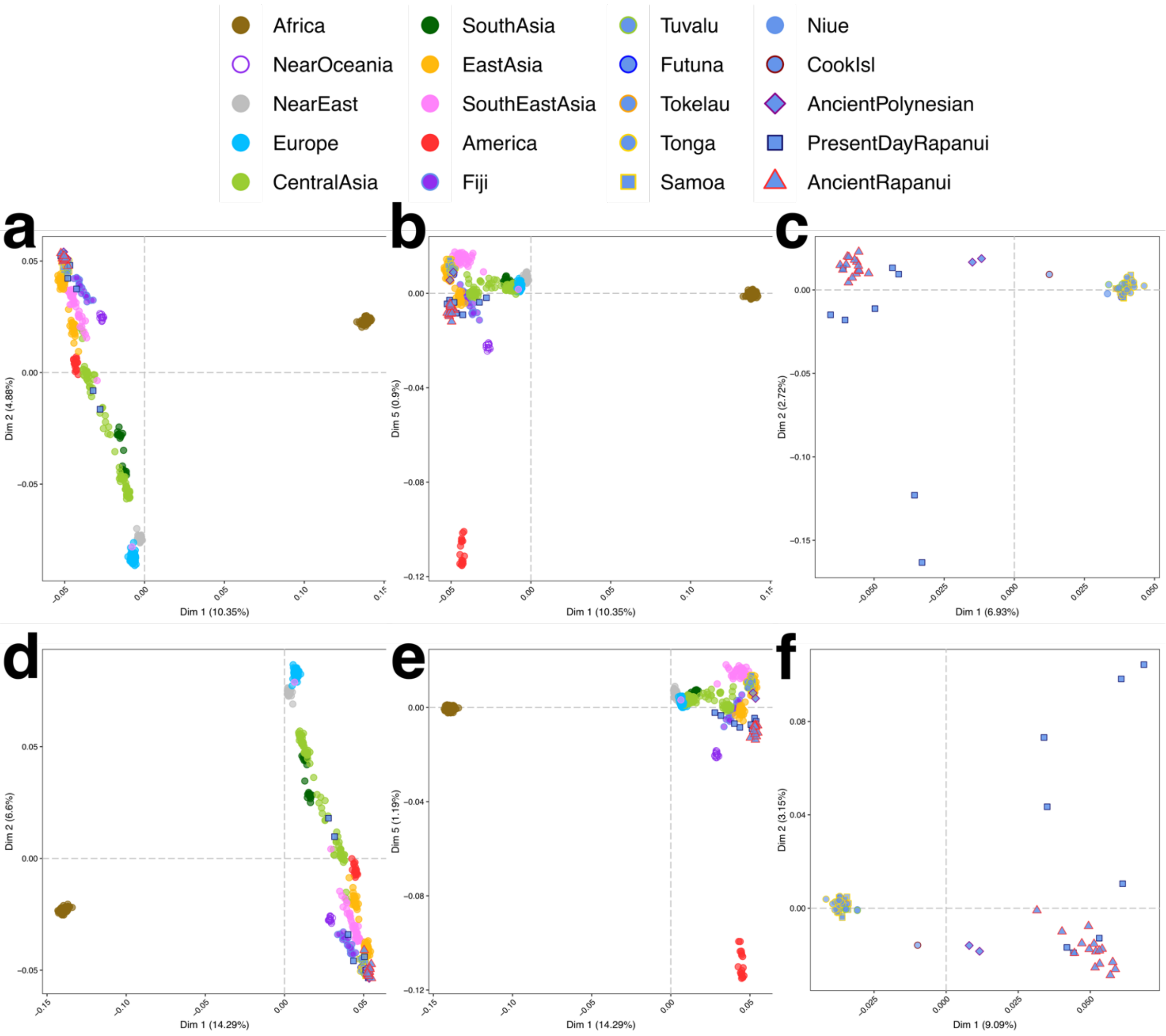

Figure S11. Multidimensional scaling (MDS) of the identity-by-state distance matrix for 'Ancient Rapanui' and a worldwide SNP array reference dataset. a,d. Dimensions 1 and 2 for the full dataset (Section S4.1) including individuals from all broad continental ancestries. **b,e.** Dimensions 1 and 5 for the full dataset. Dimension 5 separates individuals carrying Native American ancestry. **c,f.** Dimensions 1 and 2 for a subset of the data restricted to Polynesian individuals. Top panels (a,b,c) correspond to data based on pseudo-haploid calls. Bottom panels (d,e,f) correspond to data based on diploid genotypes for present-day individuals and imputed diploid genotype calls for ancient individuals. Non-Polynesian individuals are coloured according to their broad continental ancestry and Polynesian individuals are coloured according to their island of origin.

S7. *f*-statistics

S7.1. Methods

We computed f_3-statistics, *D*-statistics and f_4-ratios [69] to investigate the genomic history of ancient and present-day Rapanui. We used *FrAnTK* [70] to compute these *f*-statistics on the SNP array (Section S4.1) and SGDP datasets (Section S4.3) considering pseudo-haploid calls and imputed diploid genotypes for ancient individuals. For each statistic, we estimated its standard error using a weighted block jackknife procedure over 5Mb blocks [69,71]. For *D*-statistics, we used the estimated standard errors to compute *Z*-scores. Based on these *Z*-scores, we consider that a *D*-statistic deviates significantly from D=0 if $|Z|$>3.3, which corresponds to a *p*-value of ~0.001. Furthermore, to explore the effect of potential additional error derived from ancient DNA *post-mortem* damage on *D*-statistics, we reran each test after excluding transition polymorphisms [72].

S7.2. Ancient individuals are most closely related to present-day Rapanui

We computed outgroup f_3-statistics of the form f_3*(Yoruba; X, Ancient Individuals)* to explore which is the ancient or present-day population in the SNP array reference dataset (Section S4.1) that shares the most drift with the 15 'Ancient Rapanui' individuals. Since the MDS (Section S6), *ADMIXTURE* (Section S12), and *D*-statistics (Section S7.3) results suggest the genomic ancestry of the 15 'Ancient Rapanui' individuals is homogeneous, we pooled all individuals to obtain allele frequencies. We observed that the 'Ancient Rapanui' share the most drift with present-day Rapanui, followed by the 'Ancient Polynesian' individuals and other present-day Polynesians in an East-West order (Extended Data Figure 1, Table S4). Importantly, this pattern is consistent across the different call types for the ancient individuals, *i.e.,* pseudo-haploid calls and imputed diploid genotypes.

To substantiate these results, we compared the 'Ancient Rapanui' to a different set of Oceanian individuals genotyped using the Affymetrix Axiom LAT-1 SNP array [68,73]. Since a number of these individuals carry European admixture, for this analysis we only pooled the 'Ancient Rapanui' and 'Ancient Polynesians' (pseudohaploid calls) and we considered each reference individual separately. For each individual, we computed *f*3*(Yoruba; AncientRapanui, X)* and repeated the analysis using all the sites in the reference panel and transversion polymorphisms only. We observed that the 'Ancient Rapanui' share the most drift with present-day individuals from Rapa Nui (Figure S12, Table S5).

These results support that the 15 ancient individuals sequenced in this study are Rapanui in origin. Furthermore, they are in line with a scenario of population continuity in the island despite the strong population decline and population movements that resulted from European contact and the Peruvian slave raids during the 1860s [74,75].

S7.3. 'Ancient Rapanui' bear Native American, but no European admixture

Native American and European admixture has been documented in present-day Rapanui individuals [46,68]. To test whether the 'Ancient Rapanui' individuals carry admixture from any

of these sources we computed *D*-statistics of the form *D(Tonga, AncientRapanui; Totonac, Yoruba)* and *D(Tonga, AncientRapanui; CEU, Yoruba)*, respectively. We use individuals from Tonga [44] as a proxy for Polynesian individuals with no Native American or European admixture (Section S6,12) [44,46], Totonac individuals [45] as a proxy for Native American ancestry and the CEU population (Utah residents) [40] as a proxy for European ancestry. Since this test is aimed at detecting Native American ancestry in the Polynesian individuals,

To account for potential heterogeneity in Native American or European admixture in the 'Ancient Rapanui', we initially did not pool individuals and computed D-statistics separately for each individual. To contextualise these tests, we computed the same *D*-statistics for present-day Rapanui [46] and the two 'Ancient Polynesian' individuals [47].

For the 15 'Ancient Rapanui' and four present-day Rapanui, we found that they share significantly more alleles with Native Americans than individuals from Tonga do ($|Z|>3.3$, Figures 3, S13, Table S6). By contrast, all 15 'Ancient Rapanui' and Tongans are symmetrically related to Europeans, whereas four present-day Rapanui share significantly more alleles with Europeans (Figures 3, S13, Table S6), in agreement with [46]. Furthermore, in agreement with the expectation that they do not carry Native American or European ancestry, we do not reject that the 'Ancient Polynesians' and Tongans are symmetrically related to Native Americans and Europeans ($|Z|<3.3$), Figures 3, S13, Table S6).

We repeated the test for Native American admixture in the 'Ancient Rapanui', the present-day Rapanui and the 'Ancient Polynesians' using the Bolivian individuals in the SNP array panel (Section 4.1) as an alternative proxy. For all tests, we observed consistent results for the Totonac and the Bolivians as expected (Table S6). Since these tests are aimed solely at detecting Native American admixture in the Polynesian individuals, we expect them to be sufficiently powered regardless of the Native American population we use as a proxy. Assuming the 'Ancient Rapanui' bear Native American admixture, a *D*-statistic of the form *D(Tonga, AncientRapanui; Native American proxy, Yoruba)* will be proportional to the length of the branch between the common ancestor of the real 'admixing Native American population' and the 'Native American proxy' on the one hand, and the common ancestor of the Tongans, 'Ancient Rapanui' and the 'Native American proxy' on the other. Assuming that a) the divergence between the ancestors of Native Americans and East Asians dates to ~25 thousand years (ka) ago [18,76] and that b) the divergence between South Native American groups occurred at most 13 ka ago [49,51,53], this *D*-statistic would be proportional to the drift that leads to a small population throughout ~12 ka [69]. In the following test (using the SGDP dataset, Section S4.3) and in Section S4.5 we use a more comprehensive reference panel to explore which ancient or present-day Native American populations are most closely related to the Native American population that admixed with the ancestors of the 'Ancient Rapanui'.

To confirm these results on an independent reference panel, we conducted a generalised set of tests using the SGDP whole-genome dataset (Section S4.3). Since this panel does not include data from a population that could be used as a proxy for Polynesians not carrying Native American or European admixture [30], we used the two 'Ancient Polynesian' individuals as such and computed *D*-statistics of the form *D(AncientPolynesian, AncientRapanui;*

SGDP, Yoruba) for each population included in the SGDP panel. For this analysis, we computed *D*-statistics for each 'Ancient Rapanui' individual as well as for a pool containing all 15 individuals. In agreement with the results we obtained using the SNP array dataset, we found that all 'Ancient Rapanui' individuals and the two 'Ancient Polynesians' are symmetrically related to all non-Native American populations, regardless of their continental origin ($|Z|<3.3$, Figure S14, Table S7). However, 'Ancient Rapanui' individuals share significantly more alleles with Native American populations spanning from Mexico to South America, than the two 'Ancient Polynesian' individuals do ($|Z|>3.3$, Figure S14, Table S7). Altogether, these results show that the 'Ancient Rapanui', like present-day individuals from the island, carry Native American admixture. Nevertheless, they do not carry admixture from a European source or from any of the other potential sources included in the panel. That we obtain consistent results when we repeat each test using pseudo-haploid calls, imputed diploid genotypes and transversion sites only (on the SNP array and SGDP datasets) suggest that these results are unlikely to be driven by errors derived from ancient DNA *post-mortem* damage or the diploid genotype imputation procedure (Figures S13,S14, Tables S6,7).

S7.4. Reanalysis of low-depth ancient Rapanui genome data

A previous study of low-depth (0.0004-0.0041X) genome data from ancient Rapanui individuals did not find evidence of Native American admixture before and after European contact [37]. While the genomes in our study were sequenced at a higher depth, we conducted several experiments to reconcile our results with this previous observation. In particular, we ran a downsampling experiment to test the power of *D*-statistics coupled with the SGDP whole-genome panel (Section S4.3) to detect Native American admixture in the low-depth data. For three 'Ancient Rapanui' individuals sequenced in this study who carry Native American admixture, we sampled 25,000, 50,000, and 100,000 random reads (to match the sequencing depth in [37]), which we used to run the whole-genome *D*-statistics test detailed in Section S7.3. Across 3 independent downsampled replicates for each individual, we did not record any statistically significant deviation from *D(AncientPolynesian, AncientRapanui; SGDP, Yoruba)*=0 ($|Z|<3.3$), for any SGDP population regardless of its ancestry (Figure S15). Note that using the full sequencing data for each of the three individuals yields $|Z|>3.3$ for Native American populations when using all sites as well as transversion polymorphisms (Figures S13,S14). Interestingly, when we considered the largest downsampled dataset (100,000 random reads and all SGDP sites) the most negative *Z*-scores (indicative of admixture into the ancient individuals) roughly corresponded to Native American populations (Figure S15a, Table S8).

To investigate whether decreased statistical power could explain the absence of an admixture signal in the previously published ancient Rapanui genomes, we computed the same *D*-statistic tests for the five individuals from [37] (Section S4.6). In agreement with the original results, we did not find statistically significant deviations from *D*=0. However, for two of the three pre-contact individuals (RN041, RN036), some of the most negative *Z*-scores (indicative of admixture into the ancient individuals) corresponded to Native American populations in the SGDP dataset, similar to the observed for the downsampled datasets above (Figure S15, Table S9). Together with the *ADMIXTURE* results in Section S12, results

from the downsampling experiment (where the strong Native American signal is not detectable at low depths of coverage) suggest that low statistical power derived from the low depth of coverage of the original data is a likely cause for the absence of a Native American admixture signal reported previously.

S7.5. Native American ancestry in Rapanui is most closely related to Pacific Coast South Americans

We compared the Native American fraction of the 'Ancient Rapanui' genomes to ancient and present-day Native American populations. First, we focused on the inferred Native American ancestry tracts in the 'Ancient Rapanui' (Section S13) and used f_3-statistics to find the most closely related Native American population. To maximise the amount of available information, we followed a strategy for masking non-Native American ancestry tracts that differs from the standard 'diploid' masking strategy, *e.g.*, [51,77]. For each individual, instead of masking sites that are not 'homozygous' Native American, we only masked alleles that were inferred to be within a non-Native American tract, *i.e.*, at a single chromosome level, and used the remaining unmasked alleles to estimate allele frequencies across the sites included in the Native American reference dataset (Section S4.4). Using those estimated allele frequencies, we computed outgroup f_3-statistics of the form f_3*(Yoruba; Native American, Native American tracts in ancient Rapanui)*. With this approach we do not depend on a Polynesian 'baseline' population to investigate Native American admixture in the 'Ancient Rapanui', *e.g.*, *D*-statistics using 'Ancient Polynesians'. We found that the Native American genome fraction of the 'Ancient Rapanui' shares the most drift with ancient and present-day South American populations. In particular, Andean populations yielded the largest f_3 values followed by populations from the Southern Cone (Figures 3, S16, Table S10). Importantly, f_3-statistics were consistently lower for ancient and present-day populations east of the Andes.

As Peruvian individuals in the 1000 Genomes are related to Andean populations in the Native American reference dataset [62], we carried out two complementary analyses to test whether the differential allele sharing with Andean populations could be biased by the genomic ancestry composition of the imputation reference panel (Section S4.5). First, we re-imputed the 'Ancient Rapanui' genomes using a modified imputation reference panel from which we excluded Peruvian individuals (Section S5), and repeated the masking and f_3-statistics analyses above. The two f_3-statistics vectors resulting from the two imputation runs were strongly correlated (Figure S16a, Table S10). However, we obtained systematically larger f_3 values for the dataset imputed with the full reference panel, compared to the dataset imputed without the Peruvian individuals (Figure S16b, Table S10). We interpret that the reduced reference imputation panel resulted in less accurate imputed diploid genotypes, ultimately leading to lower f_3-values regardless of the test population.

As a second approach to rule out that imputed genotypes result in spurious excess allele sharing between 'Ancient Rapanui' and Andean populations, we computed *D*-statistics using pseudo-haploid calls for the ancient individuals. We computed *D(AncientPolynesian, AncientRapanui; Native American, Yoruba)* for the populations in the Native American reference panel (Section S4.4). Following the results in Section S7.3, 'Ancient Rapanui'

share significantly more alleles with ancient and present-day South Native American populations than 'Ancient Polynesians' do. More importantly, in agreement with the f_3-statistics based on the masked data, the largest D-statistics correspond to Andean populations (Figure S16c, Table S11). These results show that the Native American ancestry we detect in 'Ancient Rapanui' is most closely related to Pacific Coast populations. Furthermore, this pattern is unlikely to be driven by the diploid genotype imputation and local ancestry inference procedures or the Polynesian population that we use as a 'baseline' to detect Native American admixture.

To confirm the results obtained using the 'Ancient Polynesian' individuals as a contrast population, we repeated the D-statistics above using Fijians and the different Polynesian populations in the SNP array dataset (Section S4.1) as a contrast population, i.e., *D(Polynesian, Ancient Rapanui; Native American, Yoruba)*. Results are summarised in Figure S17 and Table S12. For all contrast populations, we obtained statistically significant values of D indicating that the 'Ancient Rapanui' share more alleles with Native Americans than other Polynesians do, with the exception of present-day Rapanui. For the latter we could not reject that ancient and present-day Rapanui are symmetrically related to all tested ancient and present-day Native American populations. Furthermore, when we compared the geographic distribution of these D-statistics, we observed comparable results, regardless of the Polynesian test population (Figure S18, Table S12). These results support that the Native American component in the 'Ancient Rapanui' is most closely related to ancient and present-day Indigenous populations from western South America (particularly from the Andes), as shown by the f_3-statistics restricted to the 'Ancient Rapanui' Native American component or the D-statistics using the 'Ancient Polynesian' individuals as a contrast population. However, we caution that due to the small SNP overlap between the '1240k' SNP capture set and the Affymetrix Human SNP Array 6.0 (~200,000 sites), the resolution of these new tests is substantially lower compared to that of the f_3- and D-statistics tests described above. Moreover, we note that the interpretation of these D-statistics can be complicated as they are expected to capture the specific population history of different Polynesians, *e.g.*, D-statistics involving Fijians are larger due to excess Papuan ancestry compared to 'Ancient Rapanui'.

Although these results strongly support that Andean populations share the most drift with the Native American component in the 'Ancient Rapanui', we conducted an additional test where we extended the Native American reference dataset (Section 4.4) with present-day SNP array data from [78]. These data include individuals from the Andes, Amazonia and the Pacific Coast of South America, where present-day proxies for the Native American component in Polynesians have been identified [68]. To identify the individuals from [78] that carry European or African admixture, we first computed the following eight D-statistics for each individual (Table S13):

- Four D-statistics to identify individuals with European admixture
D(SpiritCave/USR1, present-day individual; FrenchSGDP/SpanishSGDP, Chimp)

- Four D-statistics to identify individuals with African admixture
D(SpiritCave/USR1, present-day individual; YorubaSGDP/MbutiSGDP, Chimp)

In the absence of European and African admixture, we expect D≈0 for all tests. Thus, we excluded all individuals for which at least one of the D-statistics yielded |Z|>2.57 (corresponding to a *p*-value ~ 0.01). Using the filtered dataset, we computed outgroup f_3-statistics of the form f_3*(Yoruba; Native American, Native American tracts in ancient Rapanui)* as detailed above. In agreement with other tests presented in this section, we observe that the Andean populations share the most drift with the Native American ancestry tracts in the 'Ancient Rapanui' (Figure S19, Table S14). We caution that genomic data from present-day individuals may carry genetic signatures stemming from colonial practices (that still occur today) that could obscure pre-European patterns of allele sharing. Thus, we anticipate that a better proxy for the Native American population that interacted with the ancestors could be identified as more pre-European colonisation genomic data from Polynesia and South America become available.

S7.6. Conclusions

Altogether, these *f*-statistics-based analyses show that the 'Ancient Rapanui' individuals are most closely related to present-day individuals from the island (Section S7.2). Like present-day Rapanui, 'Ancient Rapanui' carry Native American ancestry (f4-ratio estimate 6.5-12.4%, Section S7.3). Importantly, none of the 'Ancient Rapanui' individuals bear European-related ancestry (Section S7.3). Furthermore, the Native American-related genome fraction in the 'Ancient Rapanui' is most closely related to ancient and present-day populations from the South American west coast including Andean groups (Section S7.5). These findings are recapitulated by MDS (Section S6), *ADMIXTURE* (Section S12) and local ancestry inference (Section S13) analyses. That these results are consistent across different genotype calls (pseudo-haploid and imputed diploid genotypes with different reference panels) and data subsets with different error profiles (all sites and transversion polymorphisms only) supports that the observed allele sharing patterns are unlikely to be driven by biases inherent to our data. Furthermore, using downsampling experiments, we show that the apparent absence of Native American ancestry in previous ancient Rapanui sequencing data from [37], can be explained by reduced statistical power (Section S7.4).

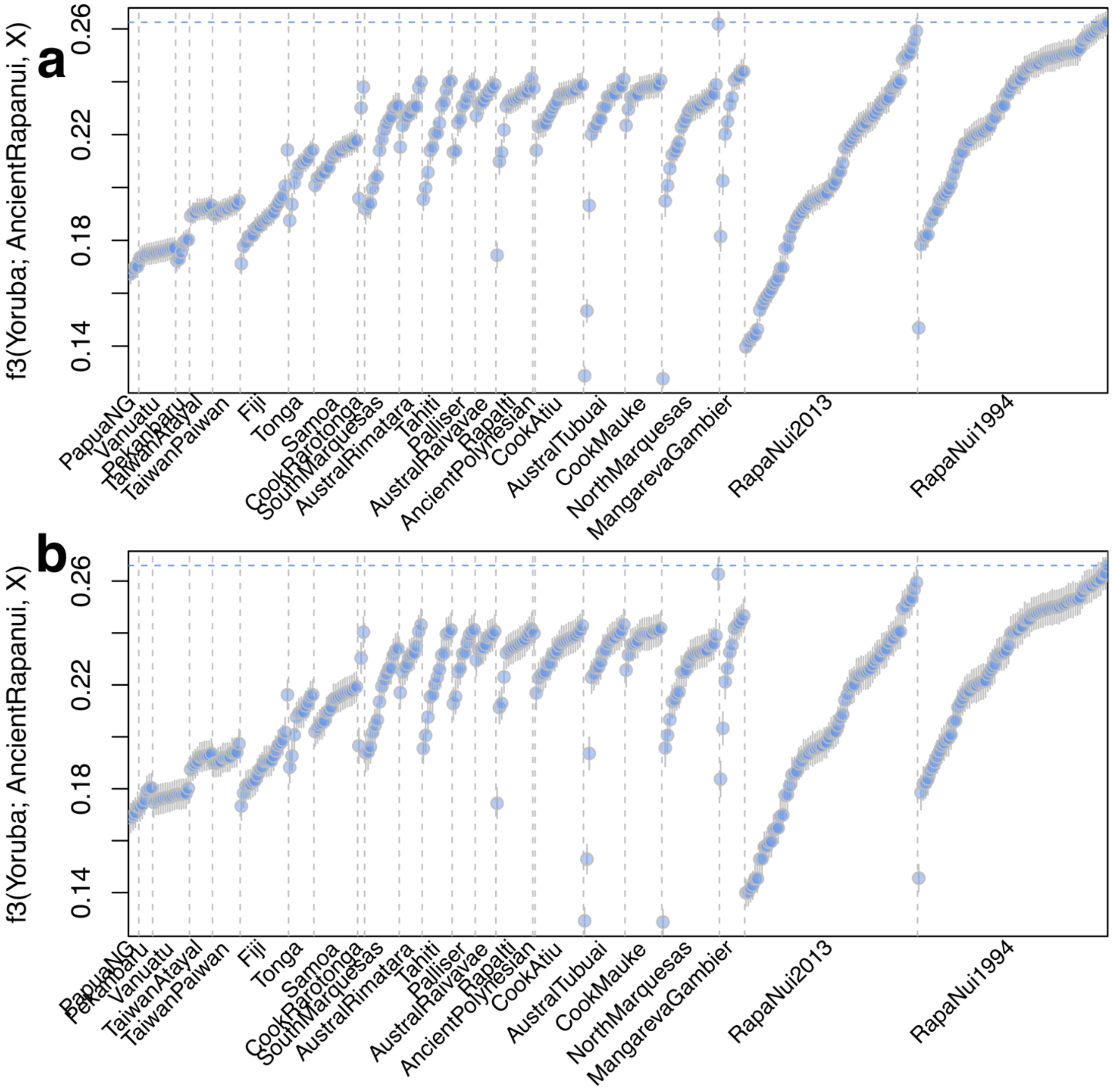

Figure S12. f3-statistics measuring shared drift between 'Ancient Rapanui' individuals and other present-day and ancient individuals in Oceania. We estimated the 'Ancient Rapanui' allele frequencies for the sites included in the SNP array data from [68,73]and computed f_3-statistics of the form *f_3(Yoruba; X, Ancient Rapanui)*. For each individual, the point represents the point estimate for f_3, and error bars correspond to the 95% confidence intervals. Individuals are ordered following their island of provenance. Horizontal dashed lines correspond to the maximum f_3-statistic (an individual among 'RapaNui1994'). **a.** f_3-statistics results for all sites. **b.** f_3-statistics results for transversion polymorphisms. Raw results for these f_3-statistics are presented in Table S5.

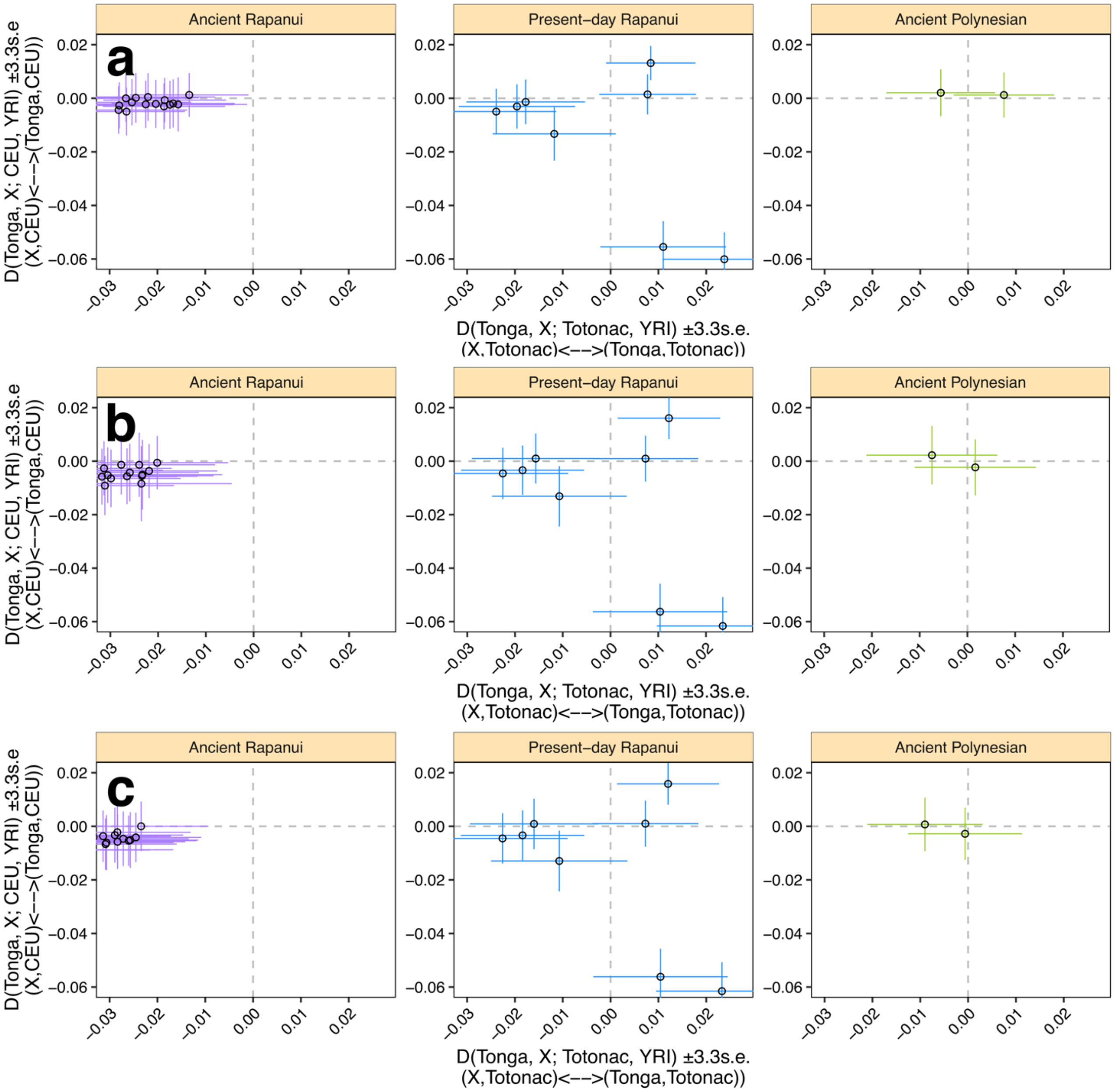

Figure S13. *D*-statistics testing for Native American (*x*-axis) and European (*y*-axis) admixture. This figure is related to Figure 3. We computed *D(Tonga, X; Totonac, Yoruba)* and *D(Tonga, X; CEU, Yoruba)* for ancient and present-day Rapanui as well as the two 'Ancient Polynesians' from an unknown island. We used the Totonac to represent Native American ancestry and Utah residents (CEU) to represent European ancestry. Figure 3 shows results for the case where we consider imputed diploid genotypes for the ancient individuals. **a.** Test results for pseudo-haploid calls for ancient individuals. **b.** Test results for pseudo-haploid calls for ancient individuals, for analyses restricted to transversion polymorphisms. **c.** Test results for imputed diploid genotypes for ancient individuals, but for analyses restricted to transversion polymorphisms. Points represent *D*-statistics, and error bars represent ~3.3 SEs (*p*-value of ~0.001 in a *Z* test; 755,094 SNPs in 5Mb blocks). Under each axis, we indicate the pair of populations with excess allele sharing, depending on the sign of *D*. Raw results are reported in Table S6.

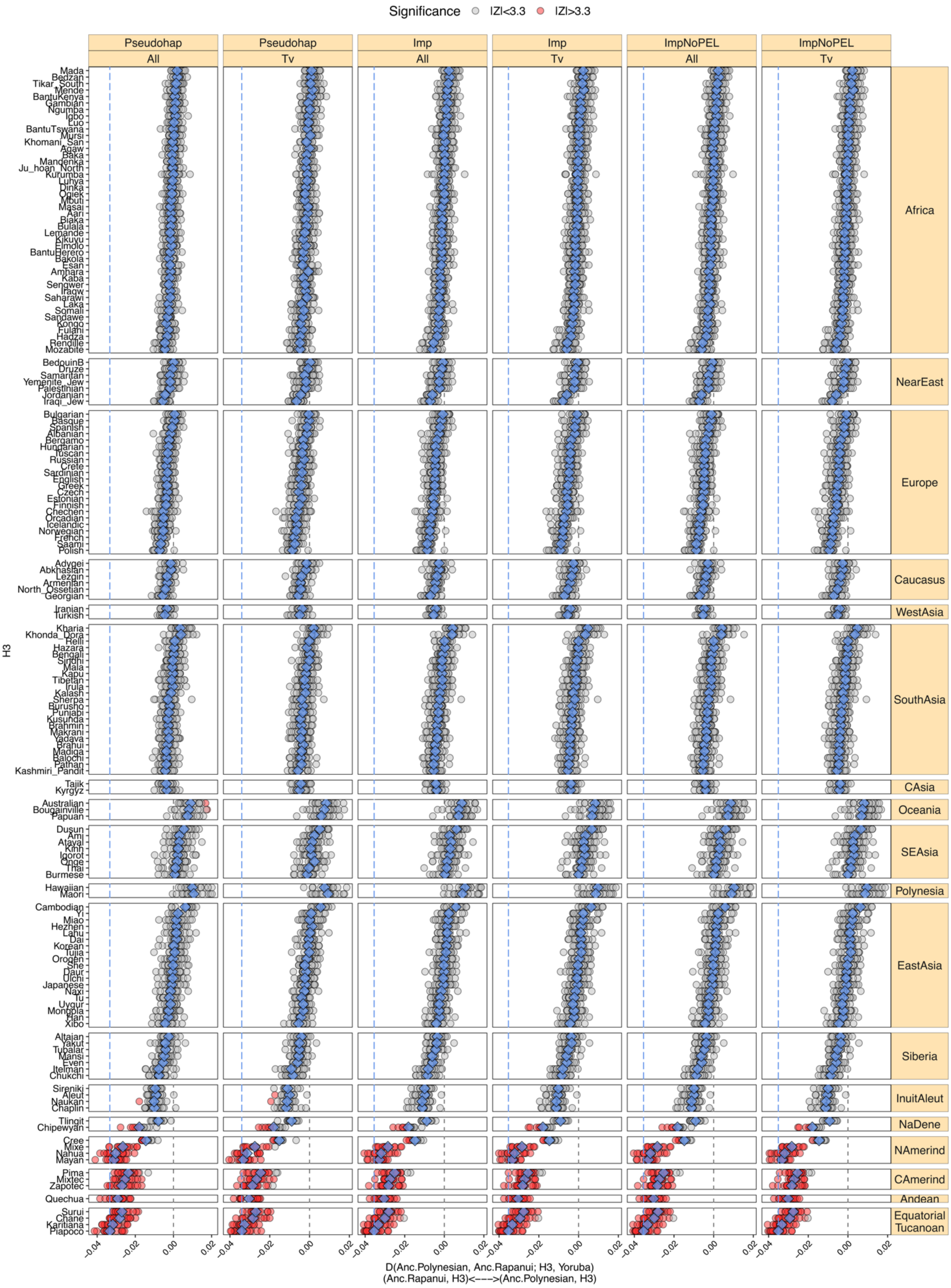

Figure S14. General *D*-statistics measuring symmetry between 'Ancient Rapanui' and 'Ancient Polynesians' with respect to a worldwide whole-genome reference panel. We

computed *D(AncientPolynesian, AncientRapanui; SGDP, Yoruba)* for all SGDP populations. We show results for each of the 15 'Ancient Rapanui' individuals (circles) and for a pool including all individuals (diamonds). Points represent *D*-statistics, and colours (red and grey) represent their statistical significance for $D \neq 0$ ($|Z|>3.3$ represents a *p*-value of ~0.001 in a *Z*-test). Grey dashed lines show $D=0$ and blue dashed lines show the maximum value of *D* for the pool of the 15 'Ancient Rapanui' individuals, for each condition. For each test, we considered pseudo-haploid calls and imputed diploid genotypes for the ancient individuals across all sites or transversion polymorphisms only (top labels). Under the *x*-axis, we indicate the pair of populations with excess allele sharing, depending on the sign of *D*. Non-Native American populations are sorted according to their geographic origin, and Native American populations (Sireniki, Aleut, Naukan, Chaplin, Tlingit, Chipewyan, Cree, Mixe, Nahua, Maya, Pima, Mixtec, Zapotec, Quechua, Suruí, Chané, Karitiana and Piapoco) are sorted according to their language family following [77]. Raw results are reported in Table S7.

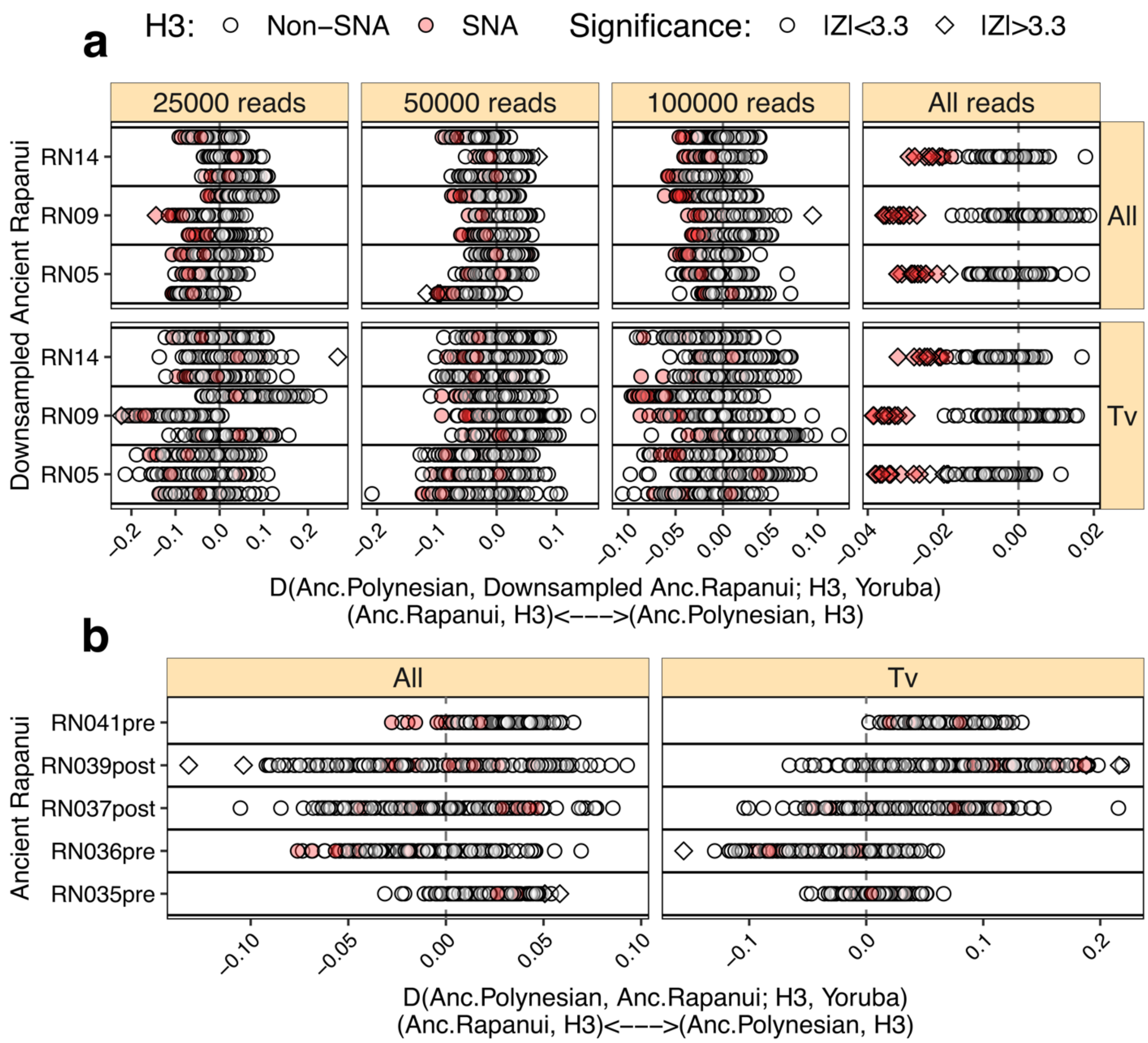

Figure S15. Statistical power tests for detecting Native American admixture in low-depth data and reanalysis of low-depth ancient Rapanui genome data. a. We downsampled three 'Ancient Rapanui' individuals to the number of reads shown at the top (to match the sequencing depth in [37]) and computed *D(AncientPolynesian, AncientRapanui; SGDP, Yoruba)* across three independent replicates, for all sites in the SGDP reference dataset or for transversion polymorphisms only (right panel labels). **b.** Similarly, we computed *D(AncientPolynesian, AncientRapanui; SGDP, Yoruba)* for the ancient genome data from [37] for all sites in the SGDP reference dataset or for transversion polymorphisms only (top panel labels). For both tests, points represent *D*-statistics, and their shapes (circles or diamonds) represent their statistical significance for $D \neq 0$ ($|Z|>3.3$ represents a *p*-value of ~0.001 in a *Z*-test). Red filled points show results for South Native American populations in the SGDP dataset. Under the *x*-axis, we indicate the pair of populations with excess allele sharing, depending on the sign of *D*. Raw results are reported in Table S8,9.

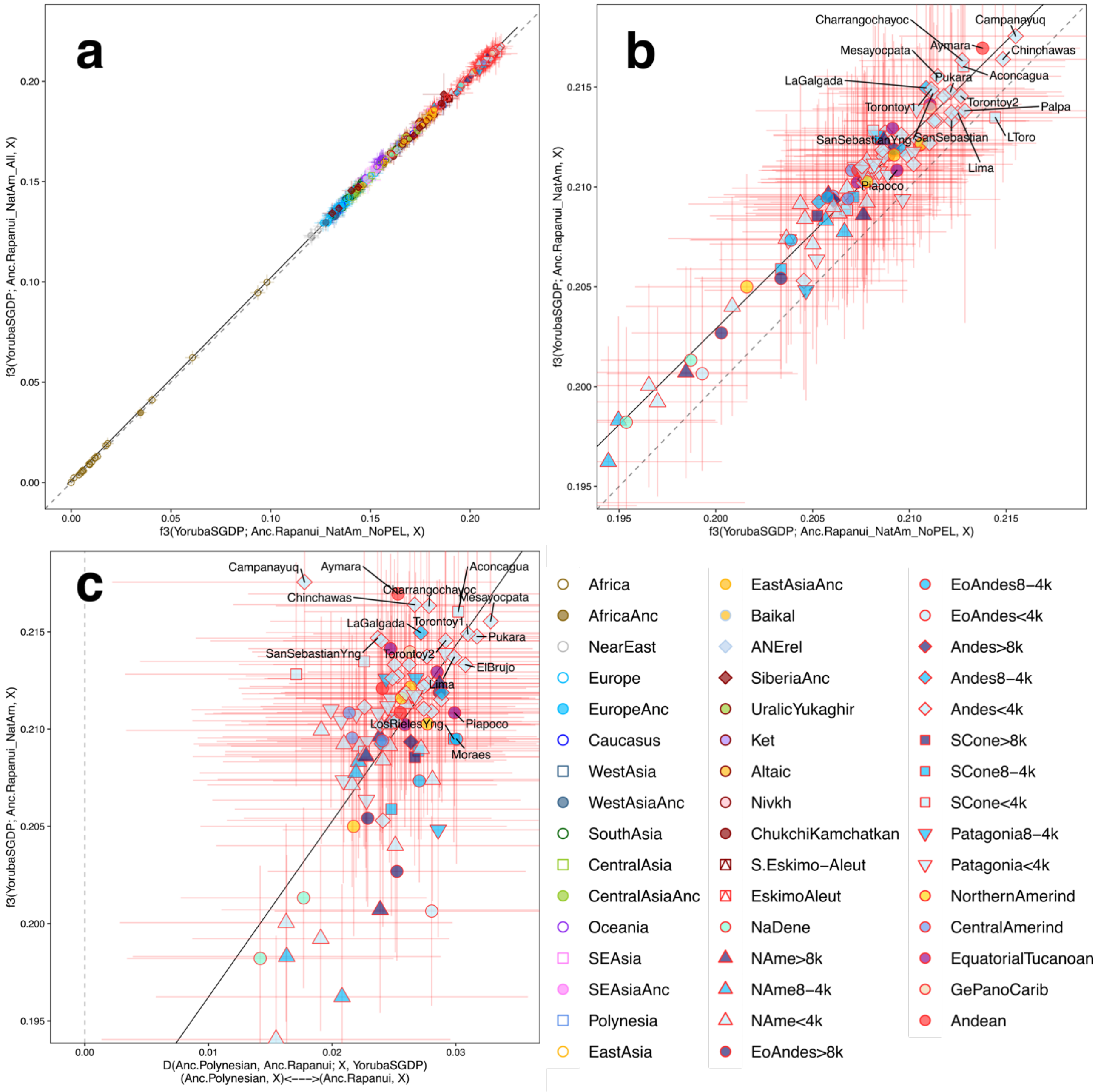

Figure S16. *f*-statistics exploring the genetic affinities between the 'Ancient Rapanui' and ancient and present-day Native Americans. We inferred the local Native American ancestry tracts in the 'Ancient Rapanui' genomes and computed *f_3(Yoruba; Native American, Native American tracts in ancient Rapanui)* for two separate imputation runs: one where we rely on the full imputation reference panel (labelled _All in the figure) and a second where we exclude Peruvian individuals (labelled _NoPel in the figure) (Section S5). **a,b**. Pairwise plot of the f3 vectors for each imputation run for all populations in the Native American reference dataset (a), and for South Native American populations only (b). Dashed lines represent the *x=y* line and solid lines represent a linear regression. Both f3 vectors are correlated suggesting the inferred genetic affinities for the Native American tracts in the 'Ancient Rapanui' are not driven by the ancestry composition of the imputation reference panel. We interpret that the reduced reference imputation panel resulted in less accurate imputed diploid genotypes, ultimately leading to lower f_3-values regardless of the test population. **c.** As a complementary approach we computed *D(AncientPolynesian,*

AncientRapanui; Native American, Yoruba) using pseudo-haploid calls for ancient individuals. We plot these *D*-statistics as a function of the f_3-statistics in b. In b and c, we label the ten populations that yield the largest f_3 or *D* values on each axis. Raw results are reported in Table S10,11.

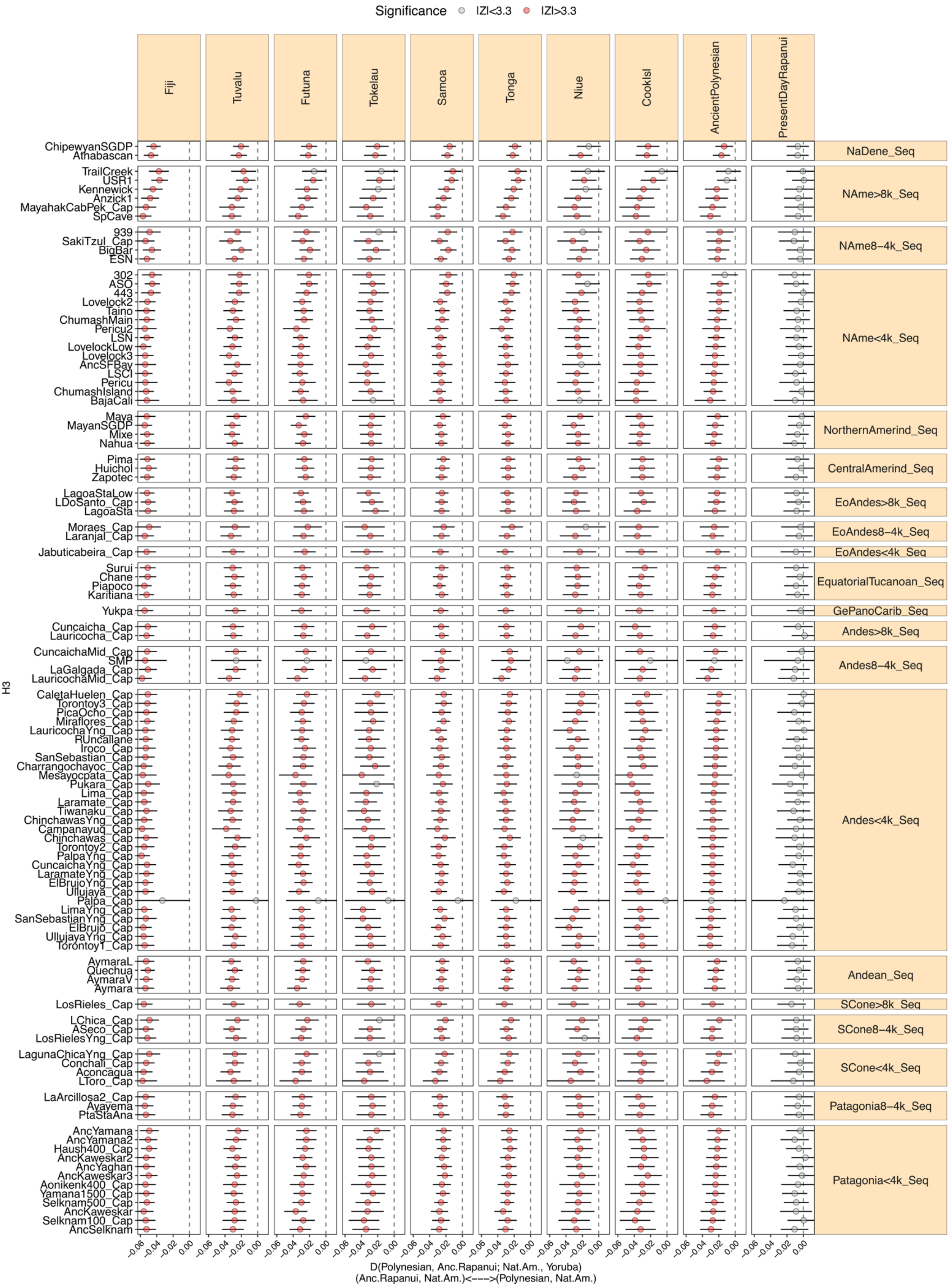

Significance |Z|<3.3 |Z|>3.3
Fiji
Tuvalu
Futuna
Tokelau
Samoa
Tonga
Niue
CookIsl
AncientPolynesian
PresentDayRapanui
H3
ChipewyanSGDP
Athabascan
NaDene_Seq
TrailCreek
USR1
Kennewick
Anzick1
MayahakCabPek_Cap
SpCave
NAme>8k_Seq
939
SakiTzul_Cap
BigBar
ESN
NAme8–4k_Seq
302
ASO
443
Lovelock2
Taino
ChumashMain
Pericu2
LSN
LovelockLow
Lovelock3
AncSFBay
LSCI
Pericu
ChumashIsland
BajaCali
NAme<4k_Seq
Maya
MayanSGDP
Mixe
Nahua
NorthernAmerind_Seq
Pima
Huichol
Zapotec
CentralAmerind_Seq
LagoaStaLow
LDoSanto_Cap
LagoaSta
EoAndes>8k_Seq
Moraes_Cap
Laranjal_Cap
EoAndes8–4k_Seq
Jabuticabeira_Cap
EoAndes<4k_Seq
Surui
Chane
Piapoco
Karitiana
EquatorialTucanoan_Seq
Yukpa
GePanoCarib_Seq
Cuncaicha_Cap
Lauricocha_Cap
Andes>8k_Seq
CuncaichaMid_Cap
SMP
LaGalgada_Cap
LauricochaMid_Cap
Andes8–4k_Seq
CaletaHuelen_Cap
Torontoy3_Cap
PicaOcho_Cap
Miraflores_Cap
LauricochaYng_Cap
RUncallane
Iroco_Cap
SanSebastian_Cap
Charrangochayoc_Cap
Mesayocpata_Cap
Pukara_Cap
Lima_Cap
Laramate_Cap
Tiwanaku_Cap
ChinchawasYng_Cap
Campanayuq_Cap
Chinchawas_Cap
Torontoy2_Cap
PalpaYng_Cap
CuncaichaYng_Cap
LaramateYng_Cap
ElBrujoYng_Cap
Ullujaya_Cap
Palpa_Cap
LimaYng_Cap
SanSebastianYng_Cap
ElBrujo_Cap
UllujayaYng_Cap
Torontoy1_Cap
Andes<4k_Seq
AymaraL
Quechua
AymaraV
Aymara
Andean_Seq
LosRieles_Cap
SCone>8k_Seq
LChica_Cap
ASeco_Cap
LosRielesYng_Cap
SCone8–4k_Seq
LagunaChicaYng_Cap
Conchali_Cap
Aconcagua
LToro_Cap
SCone<4k_Seq
LaArcillosa2_Cap
Ayayema
PtaStaAna
Patagonia8–4k_Seq
AncYamana
AncYamana2
Haush400_Cap
AncKaweskar2
AncYaghan
AncKaweskar3
Aonikenk400_Cap
Yamana1500_Cap
Selknam500_Cap
AncKaweskar
Selknam100_Cap
AncSelknam
Patagonia<4k_Seq
-0.06 -0.04 -0.02 0.00
D(Polynesian, Anc.Rapanui; Nat.Am., Yoruba)
(Anc.Rapanui, Nat.Am.)<--->(Polynesian, Nat.Am.)

Figure S17. ***D*-statistics measuring symmetry between 'Ancient Rapanui' and other Polynesians with respect to ancient and present-day Native Americans**. We computed *D(Fiji/Polynesian, AncientRapanui; Native American, Yoruba)* for all Polynesian populations in the SNP array dataset (Section S4.1) and all ancient and present-day populations in the Native American reference panel (Section S4.4). Points represent *D*-statistics, error bars represent ~3.3 SEs (*p*-value of ~0.001 in a *Z*-test; 188,811 SNPs in 5Mb blocks), and colours (red and grey) represent their statistical significance for $D \neq 0$ ($|Z|>3.3$ represents a *p*-value of ~0.001). Grey dashed lines show $D=0$. Under the *x*-axis, we indicate the pair of populations with excess allele sharing, depending on the sign of *D*. Present-day Native American populations are sorted according to their language family following [77] and ancient Native American populations are sorted according to their sampling location and age: 'NAme': North America, 'EoAndes': East of Andes, 'SCone': Southern Cone, >8k: ancient data pre-dating 8,000 years ago, 8-4k: ancient data ranging between 8,000 and 4,000 years ago, <4k: ancient data post-dating 4,000 years ago. Raw results are reported in Table S12.

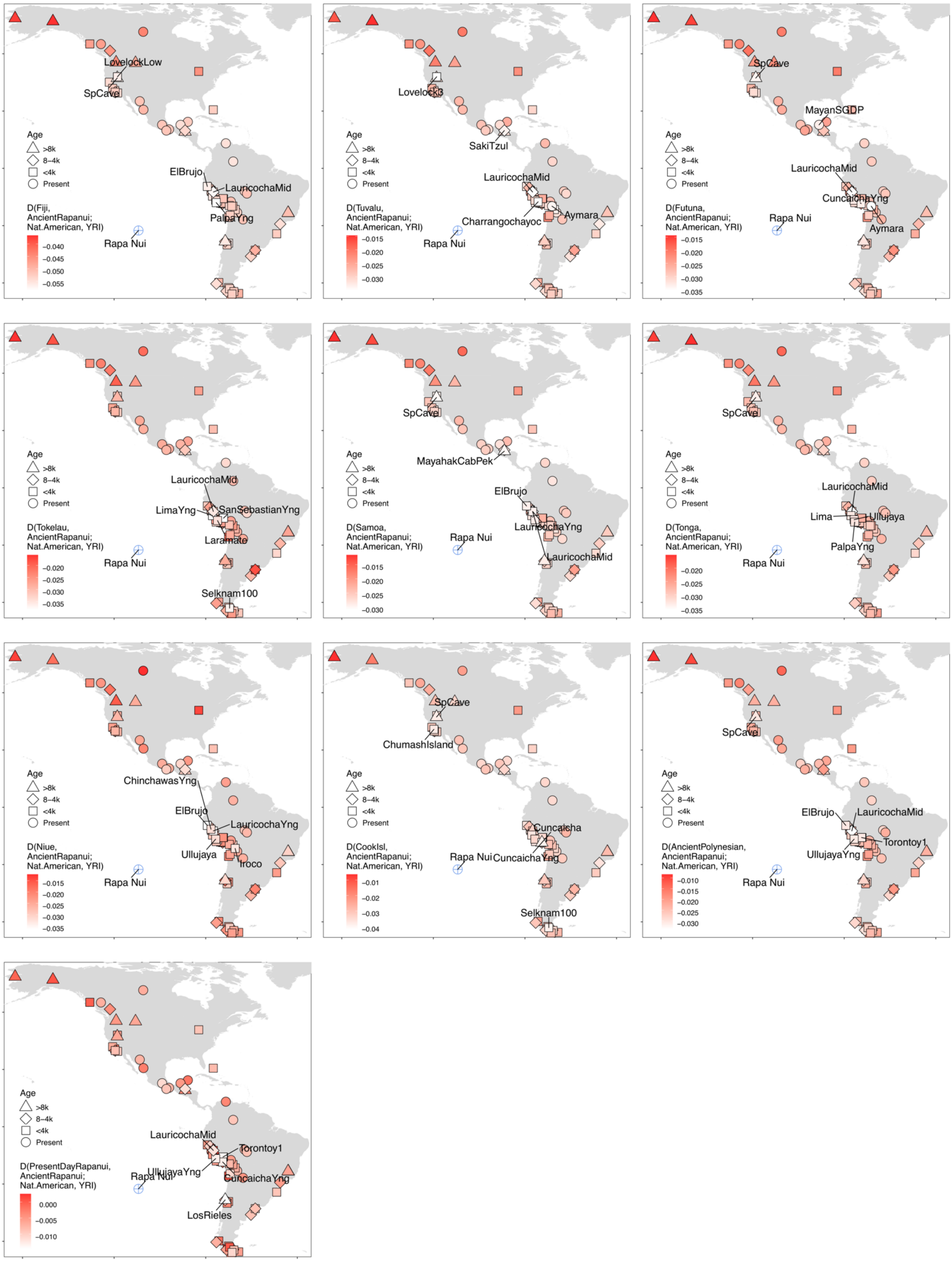

Figure S18. Geographic distributions of the D-statistics presented in Figure S17. For each Polynesian test population, we show the results for all tests based on ≥5,000

ABBA+BABA sites. Lighter colours represent greater shared drift between a Native American population and the 'Ancient Rapanui'. In each panel, we label the five Native American populations that lead to the largest D-statistics. Point shapes represent the age of Native American populations in years before present. Raw results are reported in Table S12.

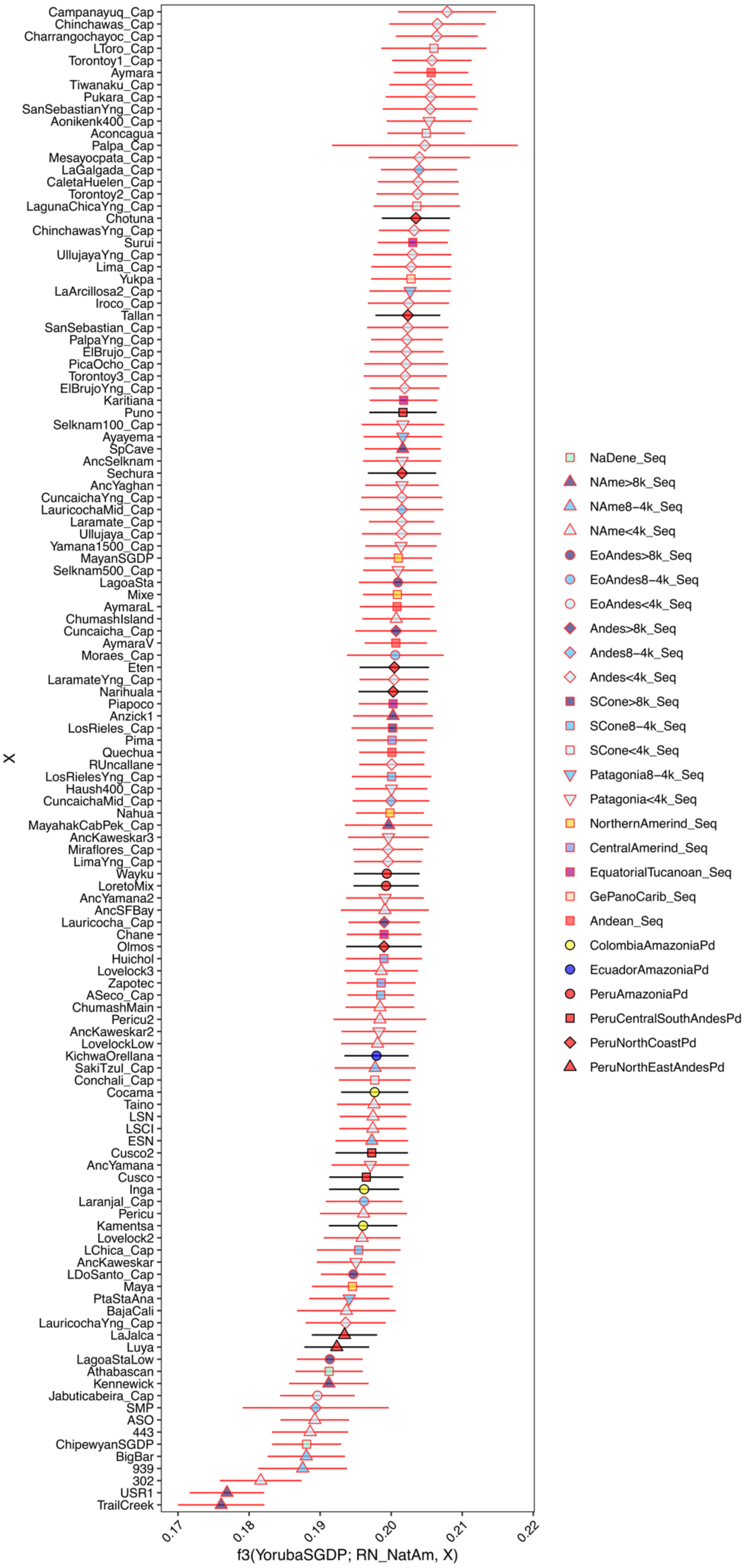

Figure S19. ***f_3*-statistics exploring the genetic affinities between the 'Ancient Rapanui' and ancient and present-day Native Americans.** We extended the Native American

reference dataset (Section S4.4) with present-day individuals from the Andes, Amazonia and the Pacific Coast (points with black outline). Using this extended dataset, we computed outgroup f_3-statistics of the form f_3*(Yoruba; Native American, Native American tracts in ancient Rapanui)*. For each population, the point represents the point estimate for f_3, and error bars correspond to the 95% confidence interval (399,323 SNPs in 5Mb blocks). Point shapes and colours represent sampling locations and age: 'NAme': North America, 'EoAndes': East of Andes, 'SCone': Southern Cone, >8k: ancient data pre-dating 8,000 years ago, 8-4k: ancient data ranging between 8,000 and 4,000 years ago, <4k: ancient data post-dating 4,000 years ago. Raw results are reported in Table S14.

S8. Identity-by-descent segment sharing

We used linkage disequilibrium information to confirm the MDS (Section S6) and *f*-statistics (Section S7) results suggesting the ancient individuals are most closely related to present day Rapanui. We used *IBDseq* [79] to infer the genomic regions that are identical by descent (IBD) between the 'Ancient Rapanui' individuals and other Remote Oceanians (Fijians and Polynesians). We ran *IBDseq* on the full SNP array dataset (Section S4.1) including a set of worldwide reference populations (to consider more accurate allele frequency estimates) and imputed diploid genotypes for all ancient individuals. In Figures 1b, S20 (Table S15), we show the cumulative length of the genome shared IBD for increasing maximum IBD segment lengths, for the 15 'Ancient Rapanui', the two 'Ancient Polynesians' and three present-day Rapanui who do not carry European admixture [46]. For reference, we compare those individuals to the Remote Oceanian individuals present in the SNP array dataset.

We observed that the 15 'Ancient Rapanui' share the largest proportion of their genomes IBD—distributed across the longest IBD segments—with each other, followed by present-day Rapanui and the two 'Ancient Polynesian' individuals. That the present-day Rapanui and not the two 'Ancient Polynesians' show a complementary pattern supports this signal is unlikely to be driven by a bias arising from diploid genotype imputation (Section S5). We caution that *IBDseq* relies on unphased genotypes and that Remote Oceanians are characterised by historically low effective population sizes, which is expected to give rise to extensive IBD sharing. Therefore, these results should be interpreted in the context of the comparisons shown in Figures 1b, S20 and not in terms of the reported absolute values.

We used *ancIBD* [80] as a complementary method to evaluate the *IBDseq* calls. Since the reference panel distributed together with *ancIBD* is based on the 1240K SNP sites, we restricted this analysis to the ancient Polynesian individuals for which whole-genome data is available ('Ancient Rapanui' and 'Ancient Polynesians') and not to other Polynesians who were genotyped using a SNP array (Section S4.1). In Figure S21 (Table S16), we show per-individual IBD sharing similar to Figure S20. The IBD segment calls from both methods follow a similar trend. In particular, we observe a close correspondence for IBD segments longer than 15cM. We also observe a linear relationship between the short IBD segments called by *IBDseq* and *ancIBD* with the latter method calling systematically fewer shorter segments (Figure S22). Therefore, in Figure 1b we only present results for the IBD segments >15cM that are not method dependent.

Furthermore, the estimated IBD sharing distribution supports that all individuals are unlikely to be related to each other as third degree (or closer) relatives [80] (Section 9). The highest sharing (>15cM) between any two individuals (RN05 and RN12) is ~900cM, which could indicate they are third- or fourth-degree relatives.

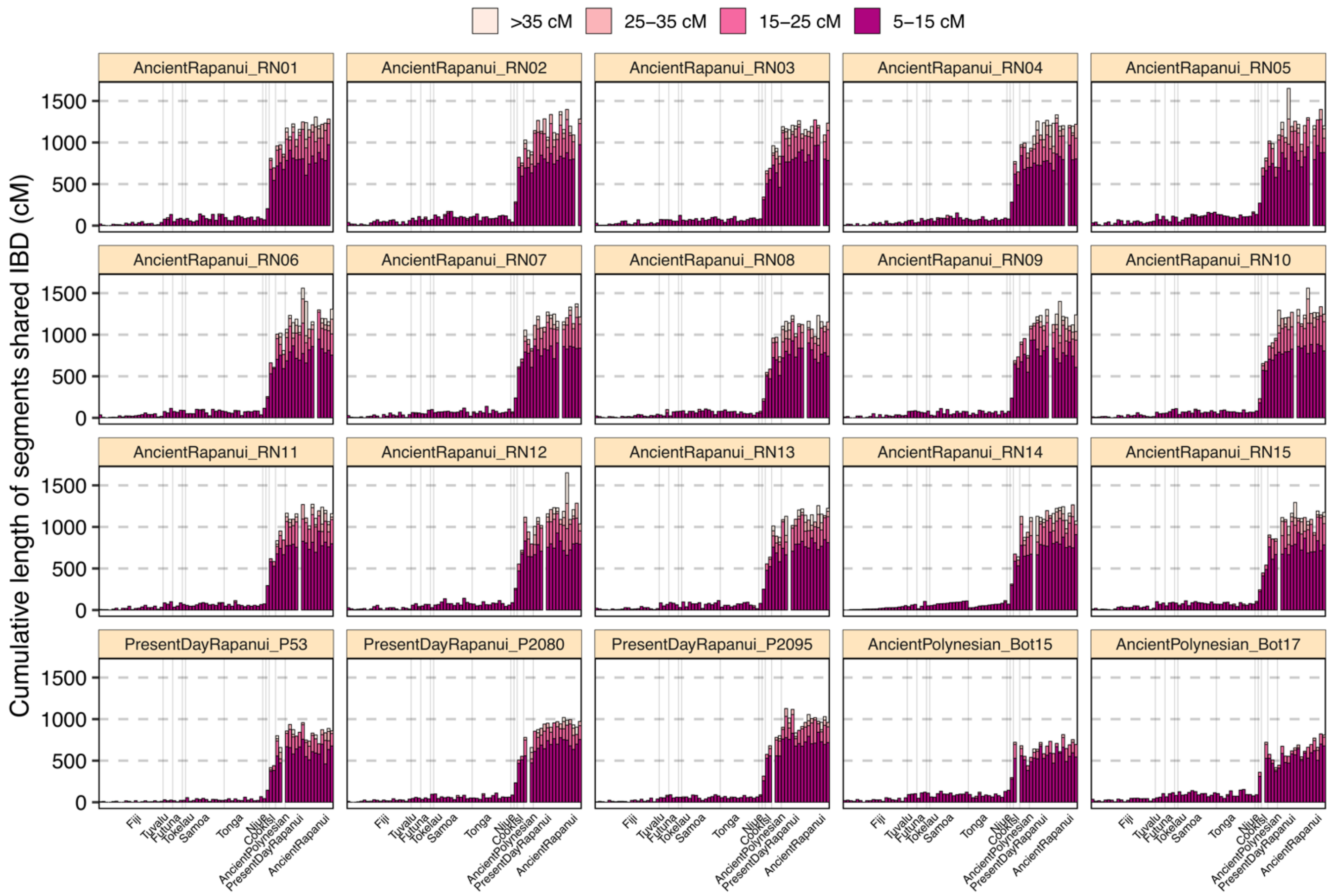

Figure S20. IBD sharing between each ancient individual and 78 ancient and present-day Polynesians and Fijians, as estimated using *IBDseq*. For each pair of individuals, we plot the cumulative length of the genome shared IBD across increasing IBD segment length bins. We show results for the 15 'Ancient Rapanui', three representative present-day Rapanui with low European admixture and two 'Ancient Polynesians' with unknown sampling location (see orange panel labels). For this analysis, we imputed the ancient individual sequence data to obtain diploid genotypes. This figure shows results for each individual included in the pooled mean data presented in Figure 1b. Individual *IBDseq* estimates stratified by length are presented in Table S15.

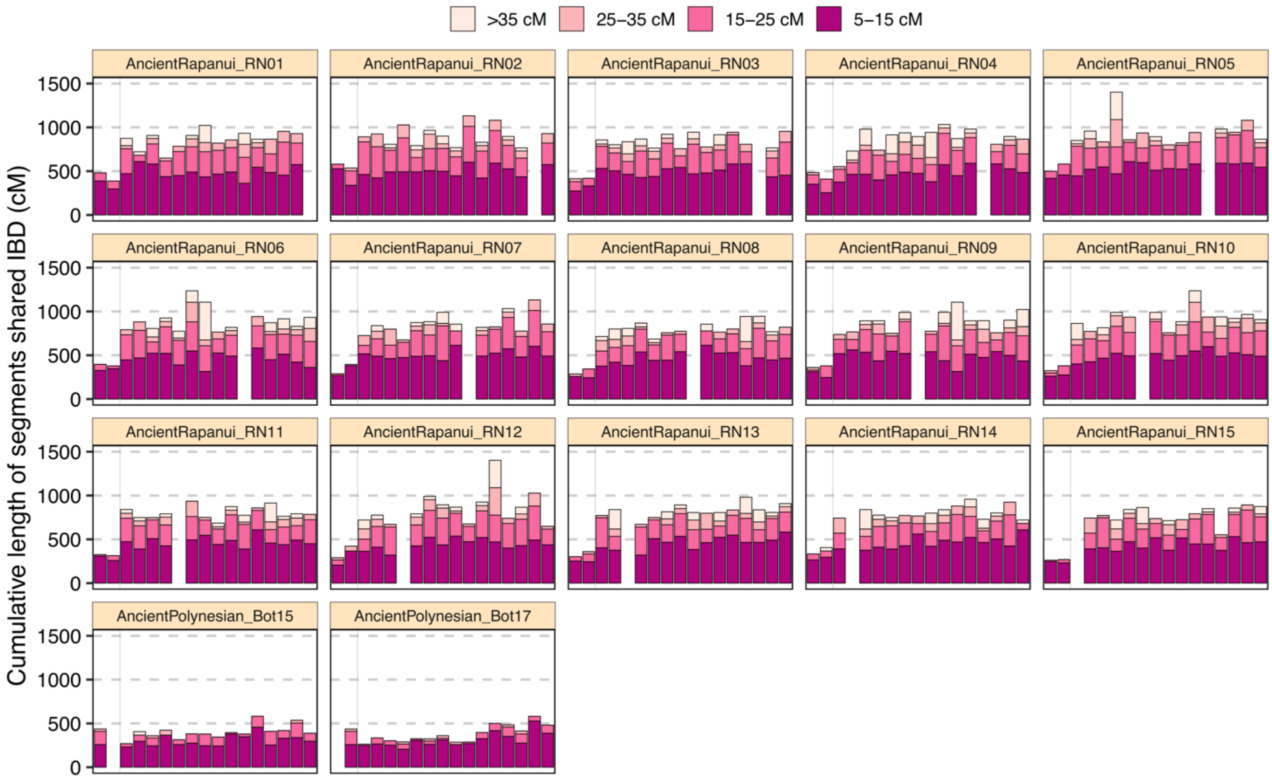

Figure S21. IBD sharing between each ancient Polynesian individual for which whole-genome data is available ('Ancient Rapanui' and 'Ancient Polynesians'), as estimated using *ancIBD*. For each pair of individuals, we plot the cumulative length of the genome shared IBD across increasing IBD segment length bins. Individual *ancIBD* estimates stratified by length are presented in Table S16.

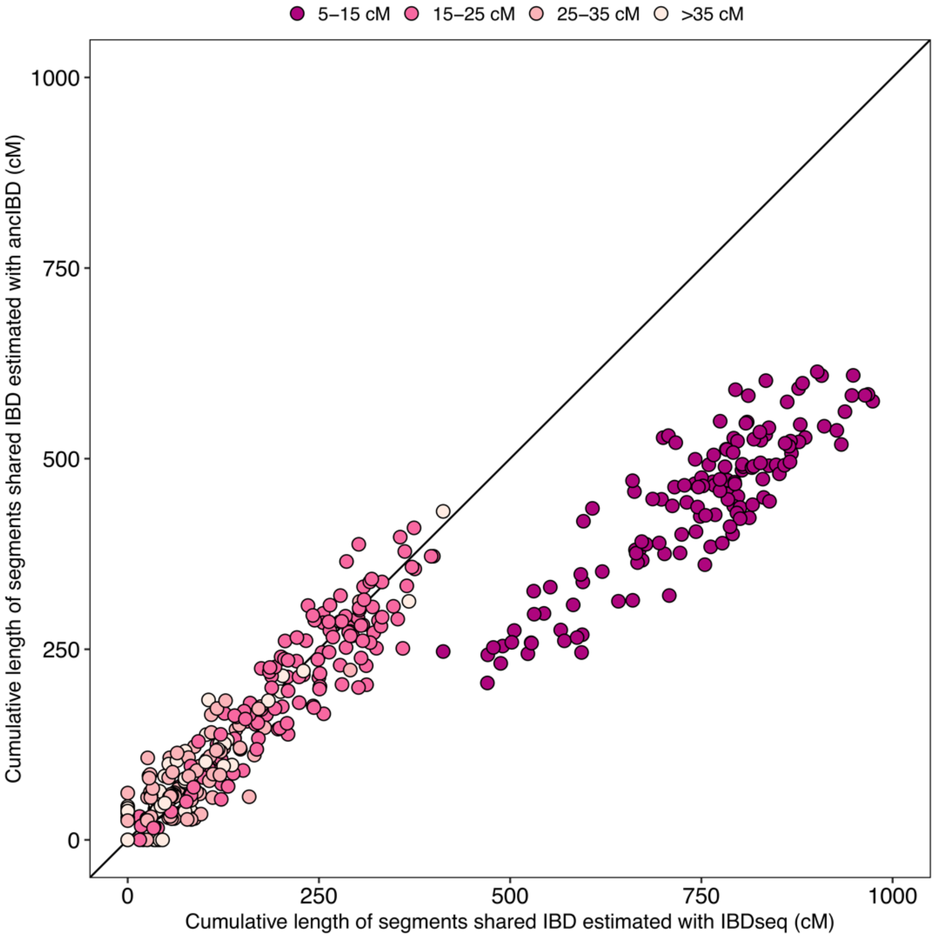

Figure S22. Comparison between IBD segments called by IBDseq and *ancIBD*. For each pair of ancient Polynesian individuals, we plot the cumulative length of the genome shared IBD across increasing IBD segment length bins (see results for each method in Figures S20 and S21). For reference, we plot the *x*=*y* line in black.

S9. Relatedness analysis of 'Ancient Rapanui' individuals

We explored the kinship relationships between the 15 'Ancient Rapanui' using two different methods: *READ* [81] and *ngsRelate2* [82]. *READ* takes as input pseudo-haploid data and can detect relatedness up to second degree of kin, such as, niece/nephew-uncle/aunt or grandchild-grandparent relationships. The input data for *ngsRelate2* are either genotype likelihoods or genotype calls and it estimates relatedness to several degrees even in the presence of consanguinity (also estimated by *ngsRelate2*).

S9.1. Methods

S9.1.1. *READ*

We estimated kinship between the 15 'Ancient Rapanui' using *READ*, with pseudo-haploid data for the same sites as in dataset S4.1. We also included the two 'Ancient Polynesians' in this analysis following the authors' recommendation of introducing individuals of similar ancestry known to be unrelated [81].

S9.1.2. *ngsRelate2*

We estimated kinship and *inbreeding coefficients* using genotype likelihoods obtained from directly calling from the non-imputed data using bcftools, *i.e.*, the input data for imputation, and using genotype calls from the imputed data (Section S5). In the case of non-imputed data, we removed transitions to decrease the *post-mortem* damage impact. The imputed data contained sites with minor allele frequency above 1% (allele frequency in the 1000 Genomes panel [62]) and with genotype probability above 0.99. We ran *ngsRelate2* with input files in VCF format (*-h vcf.gz*).

S9.2. Results

S9.2.1. *READ*

By running *READ*, we could exclude the possibility of any pair of these individuals being related up to second degree of kin. Figure S23 depicts the sorted average proportion of mismatching alleles, P_0, across 1 Mbp-long windows between any two individuals before normalisation. Here none of the pairs of individuals falls within the range of 2nd or 1st degree of kinship, that is, no pair is below the 2nd or the 1st degree dashed lines indicated in the plot. Moreover, the individual pairs with the highest values of P_0, and, therefore, expected to be less related, include an 'Ancient Polynesian' and an 'Ancient Rapanui'.

S9.2.2. *ngsRelate2*

The relatedness estimates obtained with *ngsRelate2* also showed no evidence that any two Rapanui individuals were closely related (Figure S24). Furthermore, *inbreeding coefficients* for these individuals were close to zero (Figure S24a). The 'Ancient Polynesian' individuals

were found to be the most closely related pair, but with still low relatedness coefficients (0.03 and 0.05 for low-coverage and imputed data, respectively).

We further evaluated the estimated R0 and KING coefficients (as estimated by *ngsRelate2*) against R1 following [83] (Figure S24b,c). These kinship statistics are based on identity by state instead of identity by descent, as is the case of the *ngsRelate2* coefficients (genome-wide allele sharing pattern frequencies (Jacquard [84]), relatedness and *inbreeding coefficients*) reported in Figure S24a. The considered pairs were located in a cloud around the theoretical values of unrelated individuals for both R0 vs. R1 and KING vs. R1 and no distinct patterns distinguished imputed from genotype-likelihood estimates. For more information on the theoretical values of these statistics, see [83].

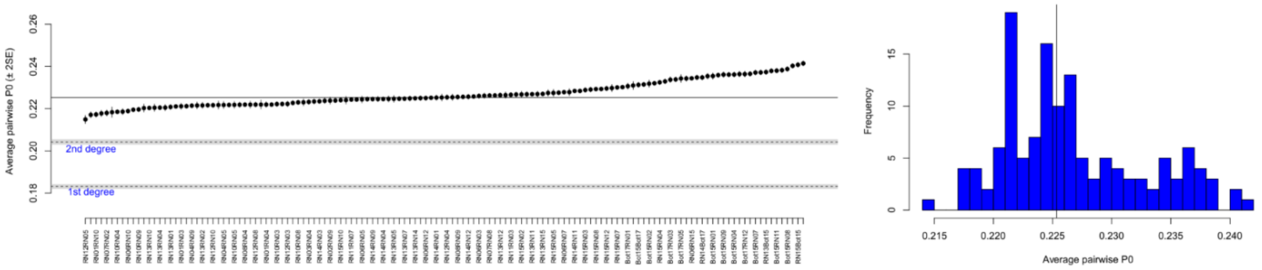

Figure S23. READ kinship results for the 'Ancient Rapanui' and the two 'Ancient Polynesians'. Left: Sorted average proportion of unshared alleles, P_0, for pairs of ancient genomes before normalizing by the median of the average P_0 values (solid line in the plot). Two standard errors of the mean are represented for each average P_0. Two dashed lines within a grey shade indicate the threshold value of P_0 within the 95% confidence interval for which a pair of individuals is classified as 2nd and 1st degree relatives. Right: histogram of the average pairwise P_0 values, where the vertical line indicates the median, as in the plot on the left.

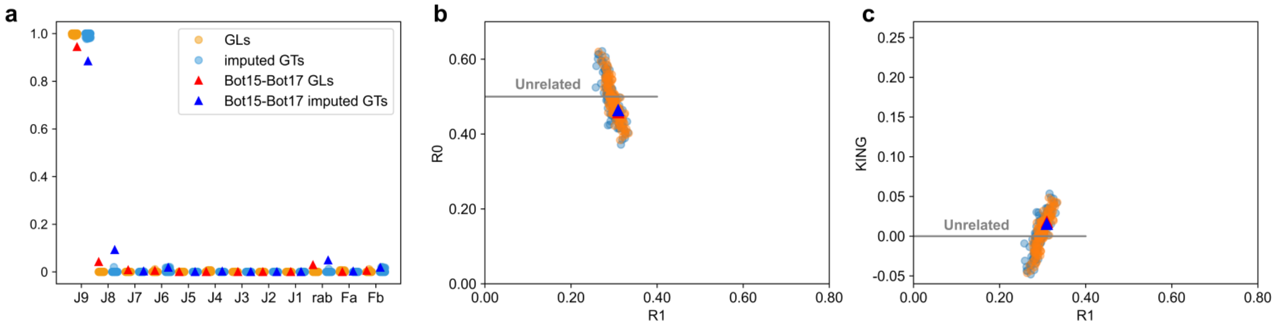

Figure S24. ***ngsRelate2* relatedness estimates for all possible pairs of 'Ancient Rapanui' individuals and the two 'Ancient Polynesians'.** We used both genotype likelihoods (orange) and imputed genotypes (blue). **a.** Estimated quantities for each pair of individuals (where the two individuals in the corresponding pair are denoted as 'a' and 'b'), including Jacquard coefficients (J9 to J1), relatedness (rab), *inbreeding* in individuals a (Fa) and b (Fb). **b.** R0 against R1 values for all individual pairs. **c.** KING against R1 values for all individual pairs. The estimated values for the pair of 'Ancient Polynesians' (Bot15 and Bot17) are represented by triangles.

S10. Runs of homozygosity (ROH)

S10.1. Methods

S10.1.1 Estimating ROH with *hapROH*

We used *hapROH* [85] to estimate ROH using pseudohaploid versions of the ancient Rapanui genomes. We generated pseudohaploid genotypes at the 1240K sites using *pileupCaller* (https://github.com/stschiff/sequenceTools) with output in eigenstrat format. We intersected the 1000 Genomes biallelic and 1240K sites to extract the imputed genotypes to be used as input for *hapROH*. As before, the imputed genotypes were filtered for MAF and GP. We ran *hapROH* in haploid (*e_model="haploid"* and *random_allele=True*) and diploid mode (*e_model="diploid_gt"* and *random_allele=False*) to estimate ROH in the pseudohaploid and imputed datasets, respectively.

S10.1.2 Estimating ROH with *PLINK*

We estimated ROH for the 15 imputed 'Ancient Rapanui' genomes, the two 'Ancient Polynesians' [47], Sumidouro5 [49] (an ancient Native American), and 22 present-day genomes, including one present-day Rapanui (P2077) [28] and 21 worldwide genomes from the SGDP [30](Table S17). The genetic data used in this analysis had been imputed and filtered (MAF>1% and GP≥0.99). We used *plink v1.9.20200712* [67] to estimate ROH with the following arguments [86]:

plink --bfile input --homozyg --homozyg-kb 500 --homozyg-gap 100 --homozyg-density 50 --homozyg-snp 50 --homozyg-window-het 1 --homozyg-window-snp 50 --homozyg-window-threshold 0.05 --out output.

As in [87], we removed ROH segments that overlapped with known assembly genome gaps (http://hgdownload.cse.ucsc.edu/goldenPath/hg19/database/gap.txt.gz).

S10.2. Assessing ROH estimated on imputed data

We started by assessing the accuracy of ROH calls in the imputed 'Ancient Rapanui' genomes. Making use of the two high-coverage genomes sequenced in this study, *i.e.*, RN13 (17X) and RN14 (26X), and *PLINK*, as described in S10.1.2, we compared ROH total length categorised by ROH size between imputed and diploid calls obtained from the high-coverage genomes. We further estimated ROH using all SNPs in the 1000G dataset (MAF>1%) and using the subset of those that are transversions. We found that, for the same SNPs subsets, the total ROH lengths in the imputed and high-coverage genomes were similar (Figure S7). Focusing on the ROH size categories, we found similar total lengths, except for RN14 when using all sites. In the case of RN14, ROH with sizes above 12 cM are practically absent from the validation dataset when considering transitions in contrast to the ROH estimated using imputed genotypes. However, this difference between validation and imputed datasets disappears when we restrict to transversions. RN14 contains both non-UDG and UDG-treated libraries, which leads to a substantial increase of transition-type

SNPs being affected by *post-mortem* damage, whereas the RN13 genome is comprised of UDG-treated libraries only. Such transition sites may be wrongly called heterozygous, thus breaking ROH segments in the validation dataset. Hence, the validation ROH estimated from transversions alone are more likely to resemble the true ones. However, we also found that the presence of deamination can affect ROH estimates even when using imputed data, as in the case of an ROH segment in chromosome 8 for RN14 (Figure S25). In this example, we detected ROH of ~9.5 cM when using all 1000 Genomes SNPs in the high-coverage and imputed datasets, while *hapROH* detected a 12 cM ROH, that was similarly found by *PLINK* when using only transversions. Overall, the similarity between validation and imputed ROH, particularly when restricting to transversions, suggests that ROH estimates using imputed genotypes and *PLINK* are reliable for the ancient genomes we sequence in this study.

S10.3. *hapROH*-inferred ROH

We used *hapROH* [85], a tool that can detect ROH longer than 4 cM in both pseudohaploid and diploid data. We compared the inferred ROH with the ROH≥4cM that we inferred using *PLINK*. We ran *hapROH* in pseudohaploid mode for the 15 'Ancient Rapanui' and the two 'Ancient Polynesians' and a subset of the present-day genomes in Figure 2a. We generated *PLINK*-inferred ROH calls using i) the transversions 1KG SNPs, and ii) the sites in the intersection of 1KG and 1240K. We found large differences when comparing *hapROH*- and *PLINK*-inferred ROH (Figure S26, Tables S18-21), particularly in the case of the Paiter Suruí genomes, for which the total ROH lengths differed by at least 68 cM owing to fewer long ROH (ROH≥20 cM) being detected using PLINK. For the Icelandic-2 genome, while *PLINK* detected a total of ~47 cM of the genome in ROH, with a single ROH larger than 12 cM (25 cM), *hapROH* detected a total ROH of 69 cM, of which 52 cM were in ROH≥20 cM. Focusing on the *PLINK* ROH estimates using transversions, we found that *hapROH* detects a higher amount of ROH≥12cM than *PLINK*: hapROH inferred ROH≥12 cM for the 15 'Ancient Rapanui', whereas *PLINK* found ROH≥12 cM for 11 of these individuals. Despite these differences, the total proportion of the genome contained in ROH remained similar across the four sets of inferred ROH (Figure S26).

While it is challenging to determine which of the three ROH sets is the most accurate, the observed differences do not affect our conclusions regarding consanguinity in the ancient Rapanui, as confirmed by the estimated low *inbreeding coefficients* (Section S9) and small effective population sizes (Section S11). Furthermore, the observed ROH distribution strongly resembles the expected for a small population according to [85]. If we consider the *hapROH* estimates for pseudohaploid data, the Paiter Suruí, who are known to have endogamic and consanguineous practices [88], have 231-241 cM of their genome in ROH≥20 cM, while only three 'Ancient Rapanui' have a single ROH longer than 20 cM (20-26 cM). Moreover, the 'Ancient Rapanui' ROH distribution is largely homogeneous across the 15 individuals, which is likely the result of a shared population history.

S10.4. Comparing ROH in ancient and present-day Rapanui individuals

To compare ROH in ancient and present-day Rapanui, we inferred ROH with *PLINK* for the Polynesians in the SNP-array dataset, which includes high-quality genetic data for five

present-day Rapanui (that were not whole-genome amplified [46,89]). Additionally, we estimated ROH for the two imputed 'Ancient Polynesians', the 15 imputed 'Ancient Rapanui' and P2077, a high-coverage present-day Rapanui genome [28], using the intersection of the 1000 Genomes and the SNP-array sites and *PLINK* as described in Section S10.1.2. In contrast to the ancient Rapanui individuals, the ROH sizes and total amounts were highly variable in the present-day Rapanui (Figure S27). For instance, P2083 had a total ROH of 29.4 cM (four segments in the 4≤ROH<8 cM size bin and one segment in the 8≤ROH<12 cM size bin), while P2094 had 582.7 cM, 41% of which were in ROH≥20 cM (241.0 cM), likely as a result of consanguinity. These individuals also had a variable amount of European-related ancestry, ranging between <1% in P2080 and P2095 and 46% in P2083 (Section S12). P2083 and P2092, with more 40% of European ancestry, had the lowest amounts of ROH. For ROH in the 4≤ROH<8 cM size bin, the ancient individuals and the present-day Rapanui with limited European-like ancestry (P2080, P2095 and P2094) show similar amounts of ROH.

S10.5. Conclusions

Comparing with worldwide populations, the sequenced 'Ancient Rapanui' have one of the largest amounts of ROH, similarly to what is observed in Native Americans and the present-day Rapanui (Section S10). Contrary to these present-day genomes, however, the 'Ancient Rapanui' do not have a large number of ROH segments larger than 12 cM, which seems to be the result of low consanguinity in Rapanui (Section S9). The overall large amount of ROH is probably a consequence of its small population size (point estimate below 2,000 individuals). These results support that there was a system in place to avoid consanguinity, as described in [90].

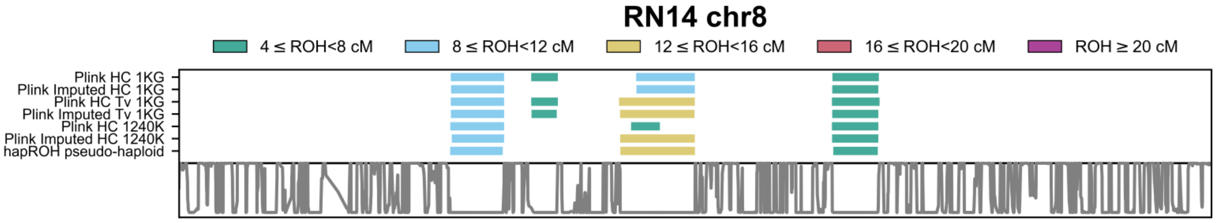

Figure S25. Zoom-in of ROH detected in chromosome 8 for RN14 (26X). We plot the ROH detected with, from top to bottom, *PLINK* and all available 1000 Genomes (1KG) sites for the high-coverage called genotypes ('Plink HC 1KG'), *PLINK* and all available 1000 Genomes sites for the high-coverage imputed genotypes ('Plink imputed HC 1KG'), *PLINK* and transversions sites in 1000 Genomes for the high-coverage called genotypes ('Plink HC Tv 1KG'), *PLINK* and transversions sites in 1000 Genomes for the high-coverage imputed genotypes ('Plink imputed Tv 1KG'), *PLINK* and the intersection between 1000 Genomes and 1240K capture sites for the high-coverage called genotypes ('Plink HC 1240K'), *PLINK* and the intersection between 1000 Genomes and 1240K capture sites for high-coverage imputed genotypes ('Plink imputed HC 1240K'), and *hapROH* for the pseudo-haploid version of RN14 ('hapROH pseudo-haploid'). The ROH segments are coloured according to their size bin. At the bottom, we show the *hapROH* posterior probability in grey.

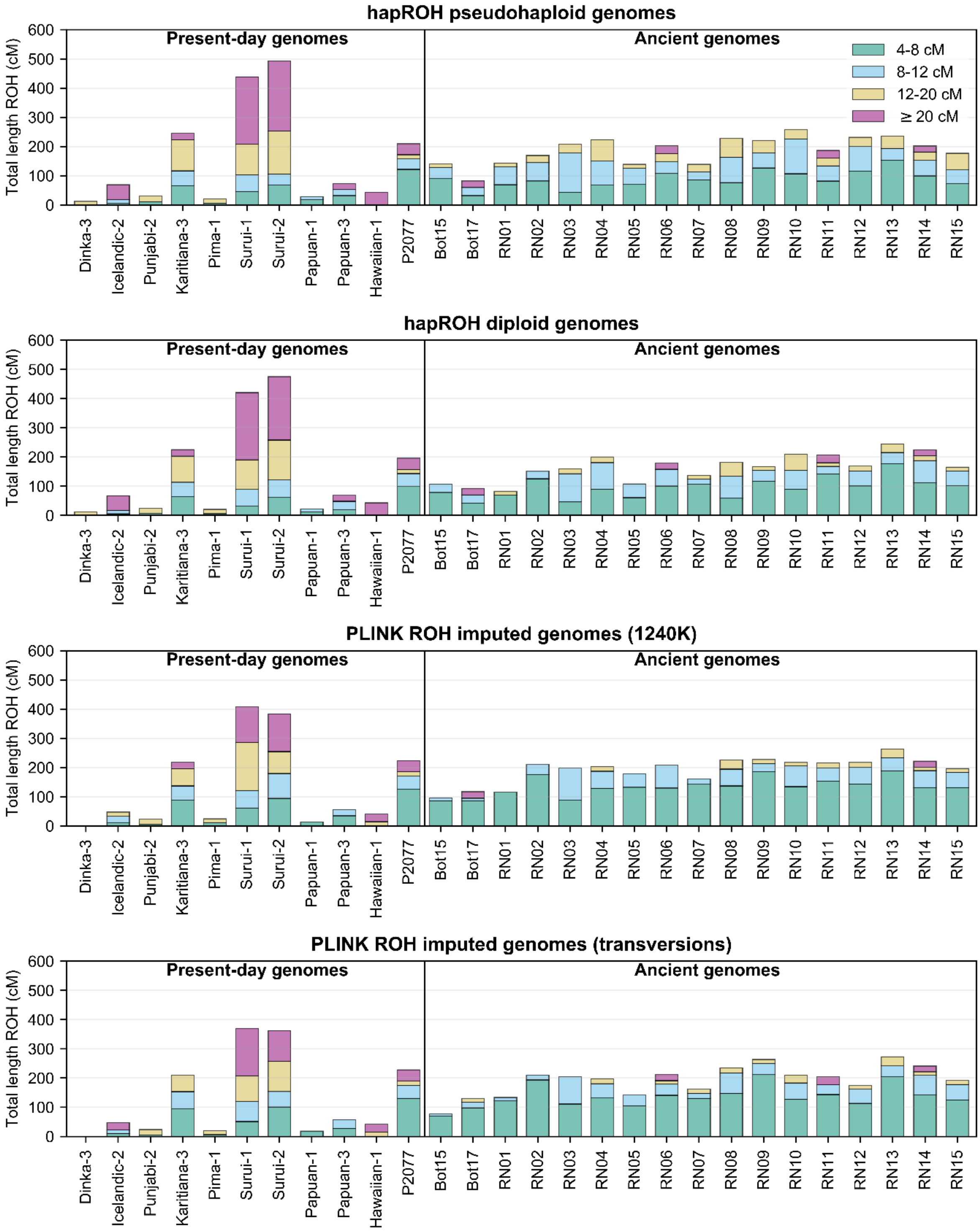

Figure S26. *HapROH* and *PLINK* inferred runs of homozygosity (ROH) estimates for worldwide and 'Ancient Rapanui' genomes. Total lengths of ROH categorised by segment size for 11 present-day genomes, the two 'Ancient Polynesians' and the 15 'Ancient Rapanui', estimated with, from top to bottom, *hapROH* and pseudohaploid genomes, *hapROH* and (imputed) diploid genomes, *PLINK* and imputed diploid genomes at the intersection of the 1000 Genomes and 1240K capture SNPs, and *PLINK* and diploid genomes when restricting to transversions in the 1000 Genomes. Individual results are presented in Tables S18-21.

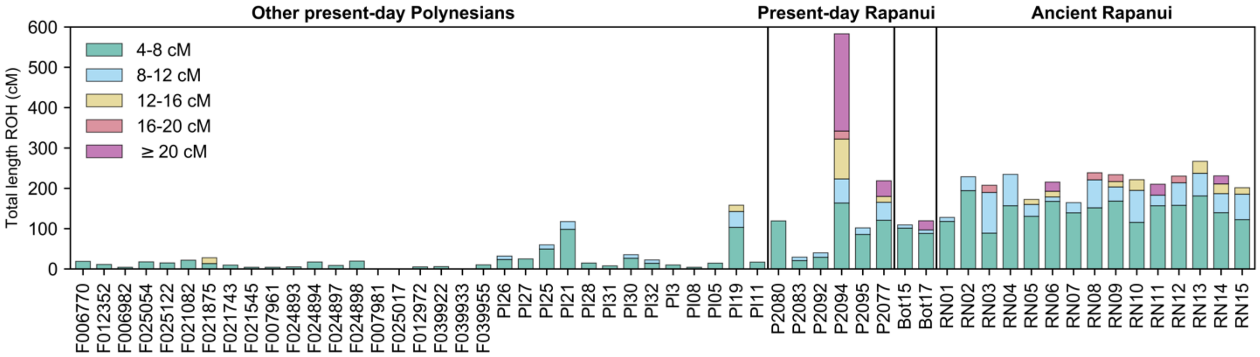

Figure S27. ROH inferred for Polynesians in SNP-array dataset. ROH inferred with *PLINK* for the Polynesian individuals in the SNP-array dataset, including five present-day Rapanui, and for genomes restricting to the overlap of 1000 Genomes sites and the SNP-array, namely, a present-day Rapanui (P2077), the two 'Ancient Polynesians' (Bot15 and Bot17) and the 15 'Ancient Rapanui'. Bars are coloured according to the length categories in the legend.

S11. Effective population size and population collapse

We used the whole-genome data from the 15 'Ancient Rapanui' individuals to test the 'ecocide' hypothesis [91,92]. In particular, we explored whether the Rapanui population went through a strong bottleneck during the 1600s. We estimated recent effective population size of both the present-day and the 'Ancient Rapanui' populations. These estimates are expected to reflect the demographic history of the pre-European contact Rapanui and the admixture events that took place post-contact [46]. Since these admixture events are expected to decrease IBD sharing between Rapanui individuals (thus resulting in seemingly larger population sizes), we conducted a simulation study to test the 'ecocide' hypothesis accounting for the admixture history of the Rapanui.

S11.1. Estimates of recent effective population size

S11.1.1. Methods

We used *HapNe-LD* [93] in diploid mode (with the HapMap recombination rates [40]) to estimate recent effective population sizes of the Rapanui over the last 100 generations. We estimated population sizes for 'Ancient Rapanui' and the present-day Rapanui individuals from [46] separately. The ancient data used in this analysis had been imputed and filtered (MAF>1% and GP≥0.99) (Section S5).

S11.1.2. Results

The estimated recent effective population sizes of present-day (eight individuals) and 'Ancient Rapanui' (15 individuals) had similar trajectories (Figure S28a), starting from an almost constant size up to ~70 generations in the past, when the population size decreased reaching a minimum at 28 and 30 generations in the past in the case of the ancient and the present-day Rapanui, respectively. We interpret that this drop corresponds to a founding event related to the early peopling of the island [44,94]. After that, in both cases, the population steadily rose until the time the individuals were alive, with median size estimates of 2,100 and 1,700 for the effective sizes of ancient and present-day Rapanui, respectively. For most of the time points, the present-day Rapanui population size was higher. To compare the population size trajectories for the ancient and present-day Rapanui, we examined their slopes at each time point. The population size slopes further show the similarity in trajectories for present-day and 'Ancient Rapanui' (Figure S28b).

We did not find evidence for a bottleneck associated with the proposed population collapse in the 1600s for either dataset. Whereas we also could not detect a bottleneck in the present-day Rapanui (associated with the slave raids and smallpox that killed most of the population, leaving only 110 survivors [74,75]), we note very recent admixture events (estimated to 1850-1870 CE [46]) might have masked such a strong bottleneck.

S11.2. Coalescent-based simulations of demographic scenarios and effect on population size

Although the *HapNe-LD* effective population size reconstructions do not include a strong bottleneck in the 1600s, as in principle several demographic histories could lead to similar *HapNe-LD* inferred effective size trajectories, we carried out a simulation study to explore whether we can detect or rule out a strong bottleneck using the 'Ancient Rapanui' whole-genome dataset. To test whether the estimated effective population size trajectory is consistent with a second strong bottleneck taking place around 1600 CE, as proposed by the population collapse hypothesis, we simulated 15 ancient genomes with a similar demographic history to the Rapanui, while varying five parameters that model a first bottleneck (*Bottleneck 1*: bottleneck time (*Tb1*) and strength (*Sb1*)), representing the peopling of the island, and a second bottleneck (*Bottleneck 2*: bottleneck time (*Tb2*) and strength (*Sb2*)), associated with the hypothesized population collapse in 1600 CE, and exponential growth with a growth rate, α, after the bottlenecks (see details below). We then compared the *HapNe-LD* effective population size estimates of the simulated and the observed data.

S11.2.1. Methods

S11.2.1.1. Coalescent-based simulations of ancient Rapanui genomes

We used *msprime* [95] to simulate 245+15 genomes to mimic a worldwide dataset, such as SGDP, and the ancient genomes in this study. We simulated diploid genomes using the HapMap [40] recombination rates, a mutation rate of 1.25×10^{-8} per unit of sequence length per generation [96,97], and a generation time equal to 29 years [98,99]. We incorporated the population genetics history depicted in Figure S29a with the following events:

i) the first split takes place 72,000 years before present (ybp) between the ancestors of Africans and non-Africans [28]
ii) the ancestors of present-day Papuans split from the other non-African populations 55,000 ypb [28]
iii) the split between Europeans and East Asian populations 42,000 ypb [28,76]
iv) the East Asian populations further split 30,000 ypb in Northern East Asians (NEA) and Southern East Asians (SEA) [18,76,100–102]
v) the ancestors of Native Americans further branch out from the NEA 25,000 [18,76]
vi) an admixture event between the Native American and European ancestor populations representing the formation of Native American genomic ancestry ~20,000 ybp (30% incoming European-related gene flow) [18,50,76]
vii) the split between the ancestors of the Polynesians and SEA 5000 ybp [44]
viii) Papuan gene flow into Polynesian ancestor population (20% gene flow) [44,103]
ix) an admixture event between Native Americans and the Rapanui ancestors 700 ypb [46,68]

All these events, their corresponding parameters and simulated effective population sizes are listed in Table S22.

Starting from a constant population size equal to 500 individuals, we changed the Rapanui population size by adding two bottlenecks followed by exponential growth. We explored the effect of these bottlenecks on the population effective size of the Rapanui by varying the

following five parameters: time of *Bottleneck 1* (*Tb1* in generations in the past), strength of *Bottleneck 1* (*Sb1*, proportion of the population remaining after *Bottleneck 1*), time of *Bottleneck 2* (*Tb2* in generations in the past), strength of *Bottleneck 2* (*Sb2*, proportion of the population remaining after *Bottleneck 2*), population growth rate (*α*, growth rate per year) (Figures 2,S29b).

Initially, we explored the parameter space with 14,400 combinations of these five parameters, without replicates ('Simulation values 1' in Table S23). For *Tb1*, we chose values that were older than the Native American gene flow into the Rapanui and we considered the Rapa Nui peopling estimated range to define the upper bound [94,104]. We then defined *Tb2* as having happened after the Native American admixture event. The growth rate was defined based on estimated pre-industrial population growth rates [105] (below 0.5% per year) and allowing it to exceed this value up to 0.7% per year. After the initial parameter space exploration, among the 14,400 simulated datasets under 'Simulation values 1', the simulations that produced *HapNe-LD* estimates closest to the observed data included a strong Bottleneck 1 (*Sb1*=0.1) taking place 47 generations in the past, a growth rate of 0.1% per year and no Bottleneck 2 (*Sb2*=1). See Section S11.2.1.2. for details on the metrics we use to compare *HapNe-LD* estimates for simulated and observed data.

To reduce the computational burden and allow for simulation replicates we defined a second set of 648 parameter combinations that we refer to as 'Simulation values 2' (Table S23). Note that it takes over two hours to complete the simulation and processing of each replicate including 260 whole-genomes from seven populations and three admixture events runs. 'Simulation values 2' is a subset of 'Simulation values 1' and includes the parameter combinations that produced *HapNe-LD* estimates closest to the observed data as well as parameter combinations for simulating a population collapse in the 1600s (*e.g.*, *Tb2*=11, *Sb2*=0.1, Table S23). More specifically, 'Simulation values 2' include the same range of values for *α* and *Tb2* as 'Simulation values 1', but we restricted *Tb1*, *Sb1* and *Sb2*. *Tb1* can take up four different values that include the extremes and midpoint of the 'Simulation values 1' range and the timing for which we obtained the simulations that resembled the observed data the most. In 'Simulation values 2', *Sb1* and *Sb2* are now allowed to take up only three different values: 0.1 (strong bottleneck), 0.5 (intermediate-strength bottleneck) and 1.0 (no bottleneck). Using 'Simulation values 2', we generated 10 replicates for each parameter combination, which resulted in 6,480 simulations.

After generating the 245+15 genomes, we removed variant sites with minor allele frequency below 1% to mimic the quality control we performed on the imputed ancient genomes. We then extracted the simulated ancient Rapanui genomes and estimated their recent effective population size using *HapNe-LD* as above.

S11.2.1.2. Comparing effective population sizes of simulated and observed data

To determine which simulated scenarios lead to effective population sizes closest to the estimated from the observed data, we tested three distance metrics, briefly described as:

- *Metric 1*: number of generations during which the estimated effective size for the simulated population is outside the 95%-CI of the estimated effective population size for the observed data (in the last 50 generations)

$$d_1 = 50 - \sum_{i=1}^{n=50} I_i$$

$$I_i = \begin{cases} 1 & \text{if} obs[Q2.5]_i \leq [Q50]_i \leq obs[Q97.5]_i \\ 0 & \text{otherwise} \end{cases}$$

- *Metric 2*: number of generations during which the slope signs of the simulated and observed data are opposite

$$d_2 = 50 \sum_{i=1}^{n=50} sign(obs[Q50]_i) \cdot sign([Q50]_i)$$

- *Metric 3*: sum of Metric 1 and half of Metric 2.

$$d_3 = d_1 + \frac{d_2}{2}$$

To decide the most suitable metric for comparing effective population size estimates for simulated and observed data, we calculated the root mean square error (RMSE) between the effective population sizes point estimates of the 'Ancient Rapanui' (Figure S30a) and the simulated genomes ('Simulation values 2' in Table S23) for the first 50 generations. We observed that *Metric 1* was more correlated with RMSE (Spearman correlation of 0.61) than the two other metrics and thus we use it in the following comparisons.

S11.2.2.2. Results

We found the smallest *Metric 1* values when 0.001≤ α ≤0.003, *Sb1*=0.1, *Tb1*=42 or 52 generations in the past (Figure S31). We performed a parametric permutation test where we split the simulations ('Simulation values 2', n=6,480, which include 10 replicates per parameter combination) into two groups: i) strong or intermediate *Bottleneck 2* (*Sb2*≤0.5) and ii) weak *Bottleneck 2* (*Sb2>0.5*). We defined the test statistic as the absolute difference of the mean values of *Metric 1* between the two groups and shuffled the data 100,000 times. We obtained a *p*-value < 1/100,000 for the observed mean *Metric 1* difference. Based on this test result we reject that the Rapanui population underwent a strong bottleneck that reduced its effective size by 50% or more after the initial peopling of the island.

Given these parameters (*Sb1*=0.1, *Tb1*=52 generations, *Sb2*=1.0, *Tb2*=21 generations, α=0.002 year^{-1}), we simulated eight present-day Rapanui-like genomes to understand why we could not detect the population crash associated with the smallpox outbreak in the 1860s that reduced the population size from 4000 to 110 individuals [75], i.e., 2.8% of the population survived. We simulated genomes (10 replicates) with and without a population collapse in

the 1860s (*Bottleneck 3*, Tb3=4 generations) and with and without European admixture (17% as in [46]) following the collapse when there was one.

Regardless of European admixture, when we modelled a strong collapse of *Sb3*=0.1 (10% survival), the recent effective population size trajectories were first constant and then decreased until the present (Figure S32a,b). We then varied the strength of the bottleneck while keeping the European admixture event. We found that the demographic model that yields the population size trajectories that are most similar to that inferred for the real data is the one without a bottleneck in the 1860s (Figure S32c and Figure S33). Given that a bottleneck of considerable strength (*Sb3={0.1, 0.3, 0.5}*) always had an effect on the effective population size and that the smallpox collapse is a well-documented event, we believe that we could not properly model the population from which the present-day Rapanui individuals originate.

After the 19th century, Rapa Nui was no longer isolated, and there was an influx of migrants, leading to significant gene flow from other populations. In fact, the genetic clustering results for these eight individuals show a high variability in ancestry, with a European-like component above 40% in two of the individuals (Figure S37). This lack of homogeneity is also reflected in the ROH distribution (Figure S27). In other words, we believe that, when it comes to modelling *Ne*, our model does not capture the main features of the sample of present-day individual genome-wide data we had access to, which in turn probably explains why we do not detect the population crash that almost decimated the Rapanui. However, ultimately, future work and data should help explain why we cannot detect the more recent bottleneck in present-day data.

S11.3. Summary statistics to test the 'ecocide' hypothesis

Up to this point, we have focused on inferring recent effective population size (*HapNe-LD* trajectories) to determine whether the Rapanui underwent a strong bottleneck between the peopling of the island and the arrival of Europeans. Such demographic events are expected to affect consanguinity levels and runs of homozygosity (ROH). Therefore, we tested whether inferred ROH distributions can allow us to distinguish between different scenarios as well as recent effective population size. To further examine whether we can reject a strong bottleneck in Rapanui, we compared ROH estimates for simulated and observed ancient genomes.

S11.3.1. Methods

To explore how ROH distributions are affected by a bottleneck of varying strength, we focused on a subset of the simulated data, for which we fixed four of the parameters, *i.e.*, Tb1, Sb1, Tb2 and α, while varying the strength of *Bottleneck 2* (Sb2). The fixed parameters were set to the values underlying the simulations that were the most similar to the observed data in terms of their inferred *HapNe-LD* trajectories (Table S24). For each parameter combination, we generated 10 replicates. We inferred ROH with *plink v1.9.20200712* using the following parameters:

plink --bfile input --homozyg --homozyg-kb 500 --homozyg-gap 100 --homozyg-density 50 --homozyg-snp 50 --homozyg-window-het 1 --homozyg-window-snp 50 --homozyg-window-threshold 0.05 --out output.

As before, we removed ROH overlapping with assembly genome gaps (http://hgdownload.cse.ucsc.edu/goldenPath/hg19/database/gap.txt.gz). In the case of the imputed ancient data, we restricted the variant sites to transversions so as to minimize the impact of *post-mortem* damage on ROH detection. In addition to *PLINK*-inferred ROH, we included the *hapROH* estimates for the ancient Rapanui (Section S10.1.2).

S11.3.2. Results

We analysed the distribution of total amount of ROH (SROH) when simulating a population with a similar history as the Rapanui, but with a Bottleneck 2 with varying strength. Regardless of the minimum ROH length that we considered for computing SROH, we found that SROH decreases with decreasing bottleneck strength (Figure S34 and Figure S35). A strong bottleneck with *Sb2*=0.1 (only 10% of the population survives) stood out as the most distinct, yielding median SROH values of 364 cM and 75 cM for ROH with at least 4 cM and 16 cM, respectively, while a bottleneck with *Sb2*=0.5 (50% of the population survives) had median SROH of 185 cM and 18 cM for the same ROH thresholds, respectively.

We compared the SROH distributions from the simulated data with the SROH distribution for the imputed 'Ancient Rapanui' genomes as obtained using *PLINK* and *hapROH*. Since *hapROH* yielded comparatively longer ROH than *PLINK*, we use its estimates as an upper bound of SROH in the ancient individuals. We found the *hapROH* and *PLINK*-inferred SROH to be similar, with differences arising at a minimum ROH of 8 cM, in which case *hapROH* SROH was greater. For SROH≥4 cM, hapROH and PLINK had identical distributions that were most similar to SROH in simulated data with *Sb2*=0.3, *Sb2*=0.5 and *Sb2*=0.7. For higher ROH thresholds, the *PLINK* SROH distribution matches better the *Sb2*=0.7 (weak Bottleneck 2) and *Sb2*=1.0 (no Bottleneck 2) SROH distributions. In conclusion, based on the whole *hapROH* and *PLINK* SROH distributions, the SROH in the 'Ancient Rapanui' could only be distinguished from SROH of simulated genomes whose populations underwent very strong bottlenecks of *Sb2*=0.1 and *Sb2*=0.2. This can be partly explained by SROH distributions in simulated genomes with *Sb2* equal to 0.3, 0.5, 0.7 and 1.0 not differing much. However, we note that their respective *HapNe-LD* curves can verify that the simulated genomes with *Sb2*=1.0 are the closest to the observed data.

S11.4. Discussion

In our estimates of effective population size, we detected only one bottleneck, for which the population reached its minimum size around 28 generations before the mean time of birth of the ancient individuals sequenced in this study, *i.e.*, around the year 1000 CE. The peopling of Rapa Nui has been dated to around 1250 CE [94], and hence this bottleneck is likely the result of this founding event (or of the several bottlenecks that Polynesians went through while exploring and settling in the different Polynesian islands) [106,107]. The population size then follows a monotonous increase with no sign of a second bottleneck.

Furthermore, coalescent-based simulations of 15 genomes under demographic scenarios with a second strong bottleneck after initial peopling and around the 18 were not consistent with the observed population size and runs of homozygosity (ROH). Simulated population sizes were closer to the observation when the growth rate was between 0.1% and 0.3%, for older and strong *Bottleneck 1* times (*Sb1*=0.1 and *Tb1*≥42 generations), and when *Bottleneck 2* was weak (*Sb2*≥0.5).

We also found the total amount of ROH distributions for data simulated under a very strong Bottleneck 2 (Sb2=0.1 and Sb2=0.2) to be clearly distinct from the observed data, thus allowing further rejection of the 'ecocide' scenario. Jared Diamond estimated that, prior to the collapse in the 1600s, there were 15,000 Rapanui inhabiting the island [91] and, if we consider the 1,500-3,000 population-size estimates for the 18th century (based on the European visitors' records [105]) as the number of individuals left after the collapse, this would represent a collapse with a strength between 0.1 and 0.2, that we can confidently reject with our effective population size and ROH analyses.

Gene flow from other Polynesian islands or regions could potentially obscure evidence of the strong collapse that presumably took place in the 17th century. While we cannot say that such contacts did not occur, we believe that they were at most infrequent, as i) the Rapanui did not have seafaring boats (the Dutch expedition described their canoes as 'leaky' ~100 years after the hypothesised collapse [105]), ii) the nearest currently inhabited Polynesian islands are Pitcairn and Mangareva at ~2000km and ~2600km respectively, and iii) Native American gene flow occurred before the 17th century and is therefore unlikely to have masked the hypothesised population collapse. However, the question of how much travel took place between Rapa Nui and the rest of Polynesia may be answered in the future as more ancient genomes from the region are sequenced.

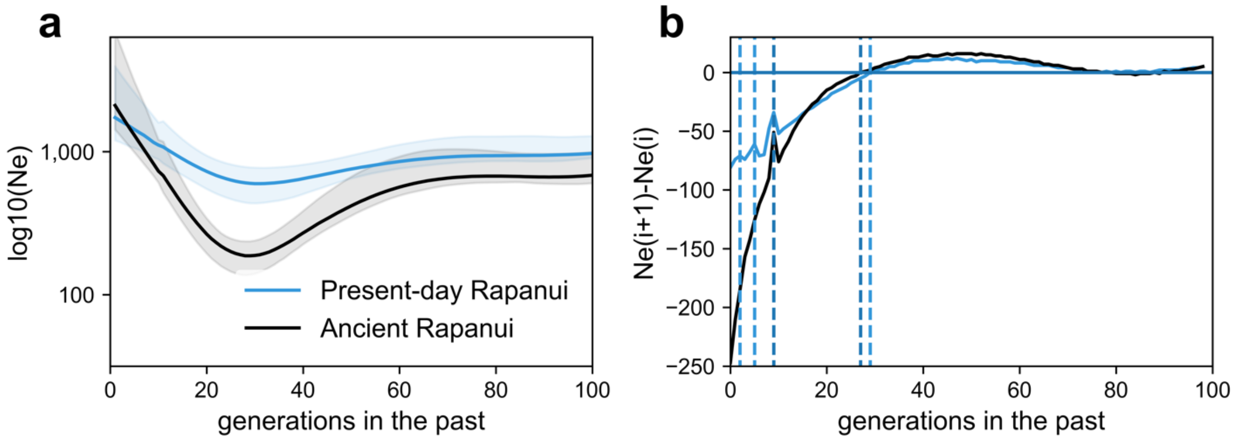

Figure S28. Recent effective population size estimates for present-day (n=8) and 'Ancient Rapanui' (n=15). a. Effective population size estimated over 100 generations in the past since the mean time of birth of the corresponding individuals. Shaded regions delimit estimates within 2.5% and 97.5% quantiles and solid line indicates the median. **b.** Approximate derivative of the median of the population size estimates as a function of time, where vertical dashed lines on the right indicate zero slope, and vertical dashed lines on the left show abrupt changes in the slope.

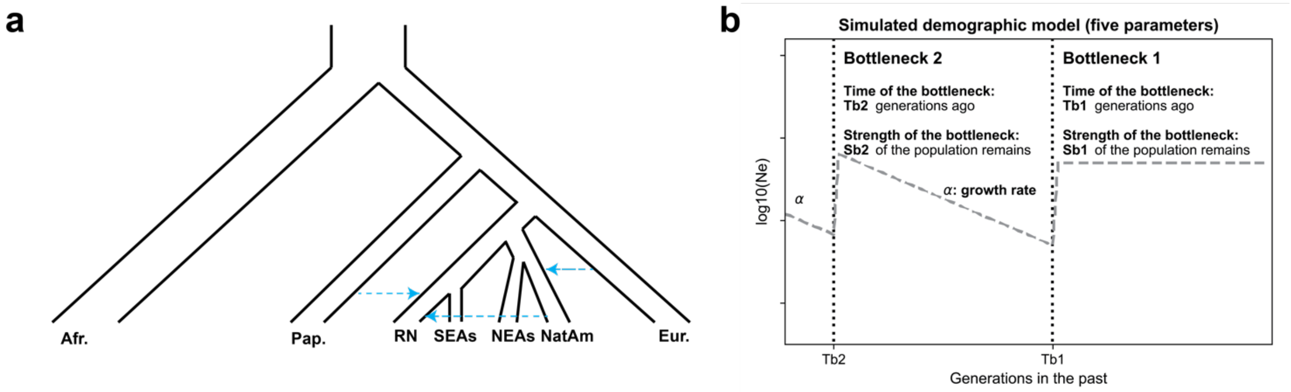

Figure S29. Simulated population history and simulated demographic model. a. Population history of the ancient Rapanui, including Native American gene flow as detected and dated in this study. **b.** Scheme representing the different varying parameters affecting recent demography of the Rapanui. Afr.: Africans; Pap.: Papuans; RN: Rapanui; SEAs: South East Asians; NEAs: North East Asians; NatAm: Native Americans; Eur.: Europeans. All these events, their corresponding parameters and simulated effective population sizes are listed in Table S22.

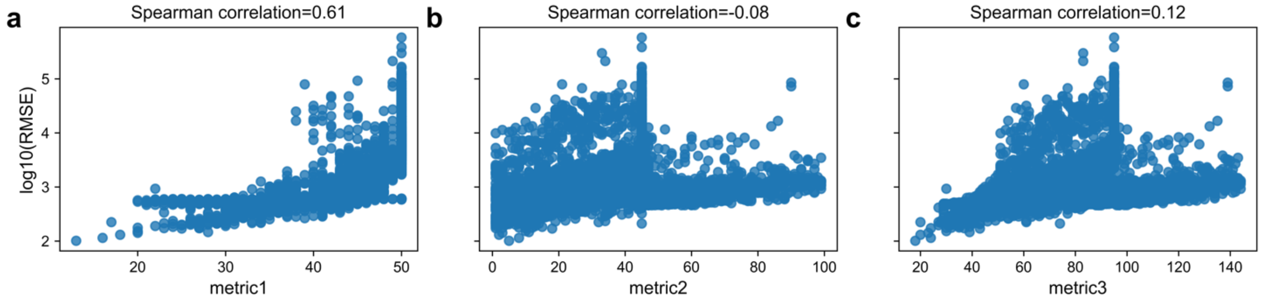

Figure S30. Root mean square error (RMSE) against the three tested distance metrics for 6,480 simulations (varying five parameters with 10 replicates). From left to right, the *x*-axis represents distance metrics 1, 2 and 3. The Spearman correlation between RMSE and each of the metrics is indicated above the respective panel.

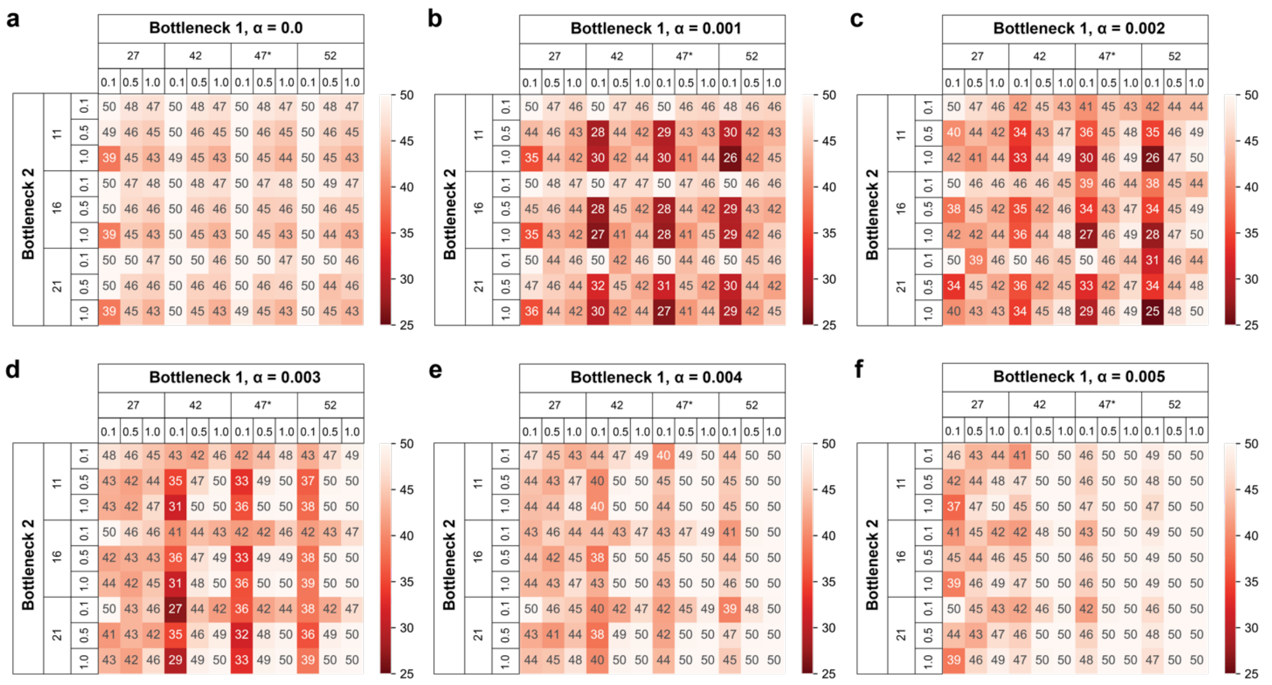

Figure S31. Average distance heatmaps across different growth rate values, α. Displayed values are the mean of distance metrics estimated from 10 replicates when varying the five parameters according to 'Simulation values 2' in Table S23. Darker red colours indicate smaller distance between observed and simulated data, *i.e.*, closer agreement. *Bottleneck 1* times and strengths are indicated on the second and third lines of the top matrix header, respectively, and *Bottleneck 2* parameters are similarly shown on the side matrix header.

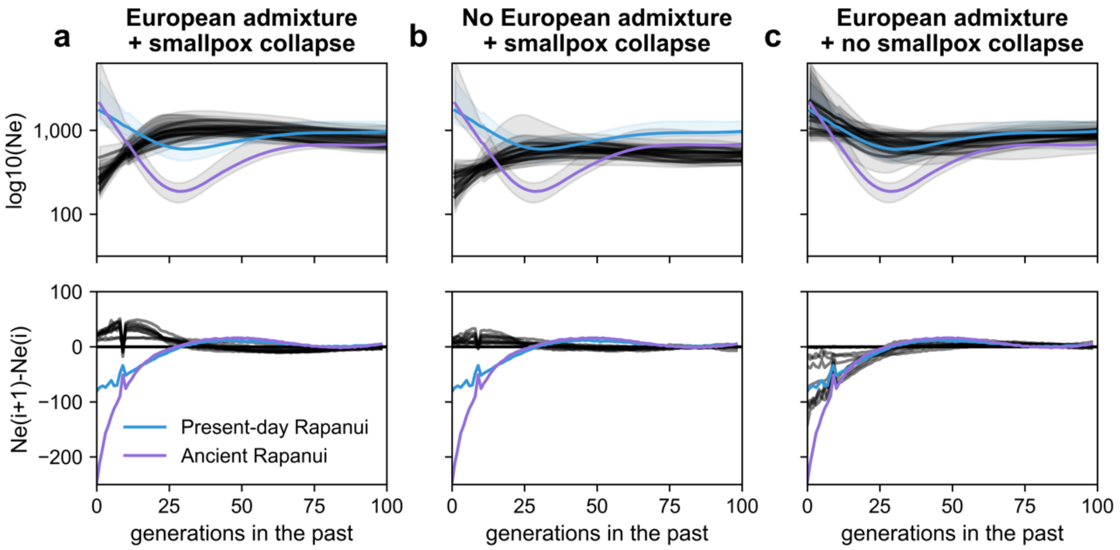

Figure S32. Recent effective population size for simulated present-day Rapanui-like genomes in models with European admixture and recent population collapse as result of the smallpox outbreak. Using the simulation parameters we found to yield the most similar population size (Ne) curves to the one obtained for the ancient Rapanui genomes, we inferred population size with *HapNe-LD* when: **a.** a strong bottleneck (*Sb3*=0.1) takes place followed by 17% of European gene flow into the population; **b.** a strong bottleneck and no European admixture; **c.** no bottleneck and 17% European admixture. The *Ne* estimates from real present-day and ancient Rapanui genetic data are represented in blue and purple, respectively, and the estimates for the simulated data are represented in black. The first row contains the logarithm base 10 of the population size as a function of generations and the second row has the slope of these curves (non-logarithmic scale). Shaded areas show *HapNe-LD* 95% bootstrap confidence intervals.

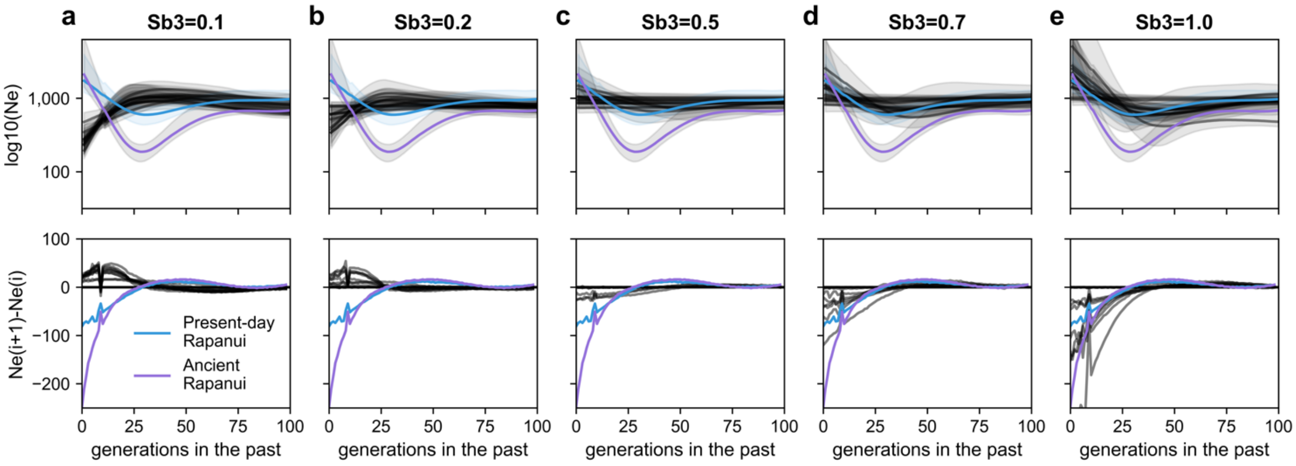

Figure S33. Effect of a 'smallpox bottleneck' strength on the effective population size of simulated present-day individuals in the presence of European admixture. We varied the strength (*Sb3*) of the 'smallpox bottleneck' between 0.1 (10% of the individuals survive) and 1.0 (no bottleneck) (a-e). The demographic model includes 17% of European gene flow one generation after the bottleneck. We simulated each set of parameters with 10 replicates (black curves). Present-day and ancient Rapanui Ne estimates are depicted in blue and purple, respectively. The first row contains the *Ne* trajectories (logarithmic scales) and the second row their rate of change. Shaded areas show *HapNe-LD* 95% bootstrap confidence intervals.

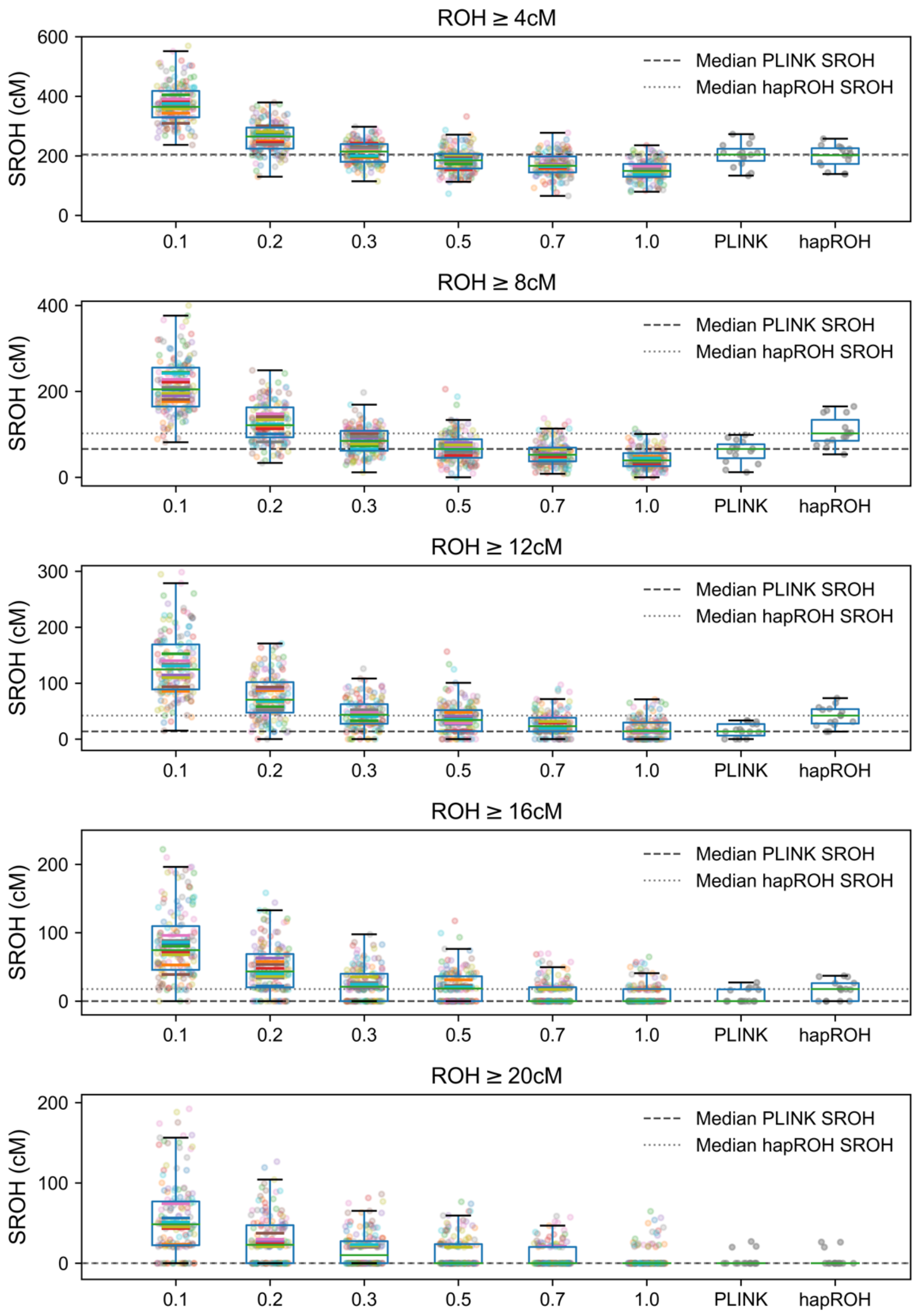

Figure S34. Comparing total amount of ROH (SROH) in 'Ancient Rapanui' and in simulated genomes while varying *Bottleneck 2* strength (*Sb2*). Boxplots of SROH for simulated genomes with *Sb2* between 0.1 and 1.0 and the imputed ancient genomes (*'PLINK'* and *'hapROH'*), where horizontal lines represent, from bottom to top, the first quartile, the median and the third quartile, and the whiskers lengths are 1.5 times the interquartile range. For each value of *Sb2*, 15 genomes were simulated from 10 populations simulated with the same parameters, that is, 10 replicates, and points from the same simulation (for the same *Sb2* value) are coloured equally with their respective medians in the same colour (the short horizontal lines).

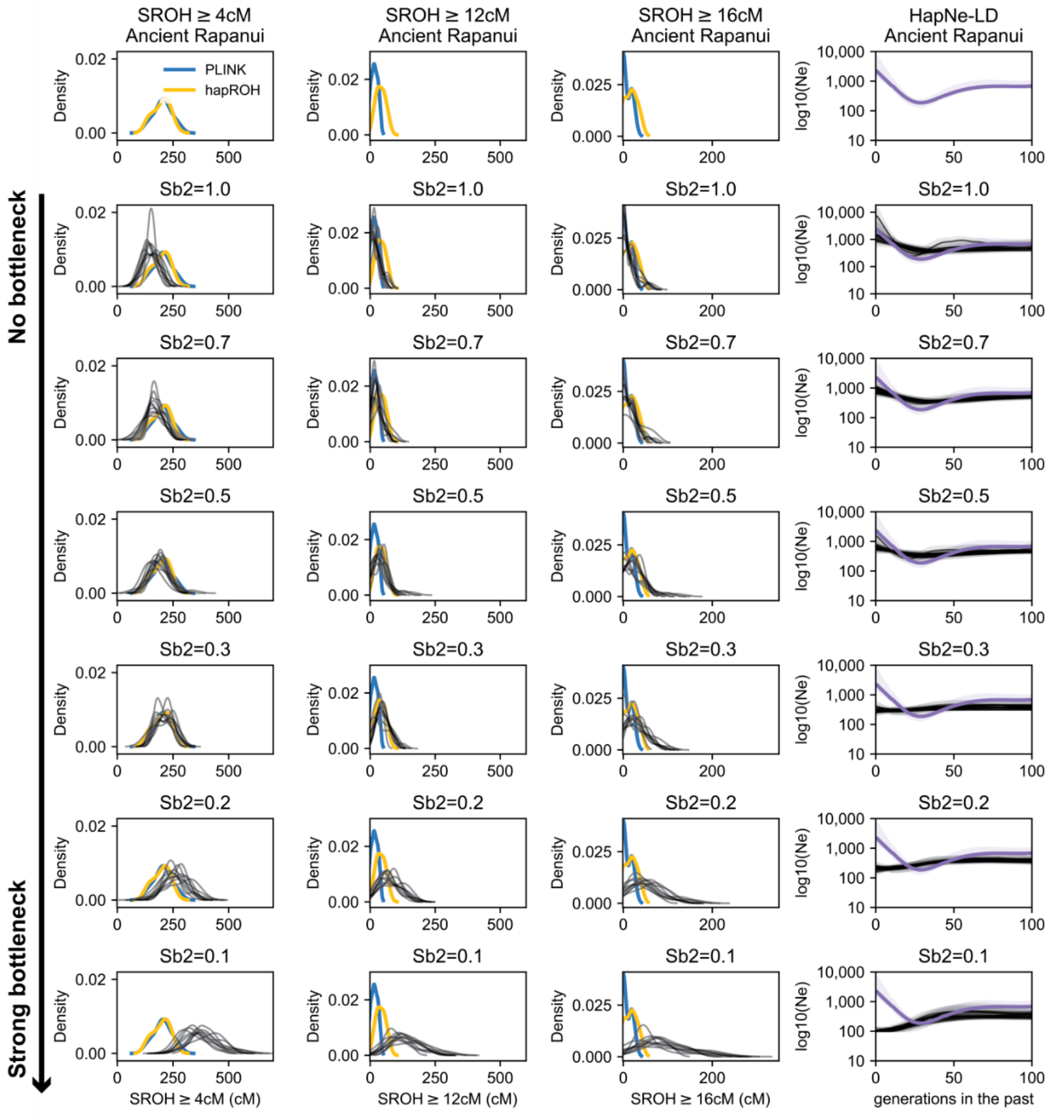

Figure S35. Using runs of homozygosity (ROH) and recent effective size (Ne) as statistics to test the 'ecocide' hypothesis. The first three columns contain the density of total amount of ROH (SROH) when considering a minimum size of 4 cM, 12 cM and 16 cM, respectively. The third column contains *Ne* estimates obtained with *HapNe-LD*. Density (*hapROH* and *PLINK*-inferred ROH in yellow and blue, respectively) and *Ne* (purple) curves estimated with the imputed 'Ancient Rapanui' data are shown on the first row and throughout, while the remaining rows include the same estimates for simulated genomes with varying *Bottleneck 2* strength (*Sb2*). We produced 10 replicates for each set of parameters.

Table S22. Demographic events and corresponding parameters, including their timing, effective population sizes and admixture proportions when relevant, used to simulate ancient Rapanui genomes. See schematic representation of the simulated scenario in Figure S29a.

**Event**	**Time in the past (years)**	**Other parameters**
Split of non-Africans from Africans [28]	72,000	N_African=20,000; N_nonAfrican=5000
Split of Papuans from other non-Africans [28]	55,000	N_Papuan=5000; N_Eurasians=10,000
Split of Europeans from EA [28,76]	42,000	N_EUR=10,000; N_EA=10,000
Split of NEA and SEA [18,76,100–102]	30,000	N_NEA=10,000; N_SEA=10,000
Split of NA from NEA [18,76]	25,000	N_NEA=10,000; N_NA=1000
Native American formation (European-related gene flow) [18,50,76]	20,000	Admixture proportion EUR=0.30
Split Polynesians from SEA [44]	5000	N_SEA=10,000; N_POL=500
Polynesian formation (Papuan gene flow) [44,103]	3000	Admixture proportion Pap=0.20
Native American gene flow into Rapanui [46,68]	700	Admixture proportion NA=0.10

Table S23. Parameter values used in coalescent-based simulations. In this table, we specify the values we used in the two rounds of simulations ('Simulation values 1' and 'Simulation values 2').

Parameter	Unit	Simulation values 1	Simulation values 2
Tb1	generation	27, 32, 37, 42, 47, 52	27, 42, 47, 52
Sb1	1	0.1, 0.2, 0.3, 0.4, 0.5, 0.6, 0.7, 0.8, 0.9, 1.0	0.1, 0.5, 1.0
Tb2	generation	11, 16, 21	11, 16, 21
Sb2	1	0.1, 0.2, 0.3, 0.4, 0.5, 0.6, 0.7, 0.8, 0.9, 1.0	0.1, 0.5, 1.0
α	$year^{-1}$	0, 0.001, 0.002, 0.003, 0.004, 0.005, 0.006, 0.007	0, 0.001, 0.002, 0.003, 0.004, 0.005

Table S24. Parameter values used in coalescent-based simulations for ROH estimates comparison. The indicated values for Tb1, Sb1, Tb2 and α represent the parameters that yielded effective population size curves that were closest to the inferred with the ancient Rapanui data. For the purpose of our analysis, we vary the strength of bottleneck 2 (Sb2) as indicated in the table.

Parameter	Unit	Simulation values
Tb1	generation	52
Sb1	1	1.0
Tb2	generation	21
Sb2	1	0.1, 0.2, 0.3, 0.5, 0.7, 1.0
α	$year^{-1}$	0.002

S12. Model-based clustering analyses (*ADMIXTURE*)

S12.1. Methods

We performed genetic clustering analyses on the 15 'Ancient Rapanui' individuals, the five ancient Rapanui individuals sequenced in [37], the two 'Ancient Polynesians' [47], and a reference panel comprising present-day individuals from around the world (Section S4.1). This reference panel is a subset of the SNP-array data (Section S4.1) with 153 individuals, including individuals from Africa (Yoruba, n=20), Europe (CEU, n=20), East Asia (Japan, n=20), America (Bolivia, n=10, and Totonac, n=7), Near Oceania (New Guinea Highlands, n=15, and Fiji, n=20) and Polynesia (Rapa Nui, n=8, Samoa, Tonga and other islands, n=33). We sampled an allele per position in the array to generate pseudo-haploid data for all genomes in this analysis. We estimated admixture proportions using *ADMIXTURE* v1.3.0 [108] in unsupervised and haploid mode with 20 replicates (20 different seeds) and chose the replicate that yielded the highest likelihood value.

S12.2. Results

We observed that the results of the genetic clustering analyses differed when we ran *ADMIXTURE* with all 15 'Ancient Rapanui' individuals or with one individual at a time. For *K*=6, when we estimated admixture proportions for the reference panel and a single ancient Rapanui, the genomic ancestry of the ancient individual was composed mostly of a Polynesian-like component (darker blue, ~90%) and a Native-American-like component (red, ~10%). However, when we estimated admixture proportions for the reference panel and the 15 'Ancient Rapanui', there was a new component that was maximised in the ancient Rapanui (darker blue), and not in the Polynesians as before. To explore this pattern, we performed this analysis with a varying number of 'Ancient Rapanui' together with the reference panel. We observed that as we increased the number of 'Ancient Rapanui' (*n*) the Native-American-like component (red) in the 'Ancient Rapanui' decreased. After *n*=9, the Native-American-like component (red) was absent in most of the 'Ancient Rapanui' whose ancestry was fully assigned to a Rapanui-like component. Furthermore, present-day (non-Rapanui) Polynesians were assigned to the Rapanui- (darker blue), Japanese- (orange) and Papuan-like (purple) components (Figure S36). These observations are likely the result of the small population size of the 'Ancient Rapanui' and the bottleneck that followed the split from the remaining Polynesians in the reference panel. In [109], a similar scenariPolynesiao is described, where *ADMIXTURE* would assign the full ancestry of the smaller, bottlenecked population that recently split from another population to its own component. Given these observations, we ran *ADMIXTURE* separately for each ancient individual (reference panel+1 ancient Polynesian).

Figure S37 shows the estimated admixture proportions for different numbers of ancestral components *K*=2-6. At *K*=6, there was a clear separation between Africans (brown), Europeans (light blue), East Asians (orange), Native Americans (red), Papuans (purple) and Polynesians (darker blue), that is, each group could be mostly represented by one ancestry component. We found that all 'Ancient Rapanui' sequenced in this study had an ancestry component that was maximised in present-day Americans (red), which varied between 6.0%

and 11.4% (mean of 8.7%) (Table S25). This component was absent in the two 'Ancient Polynesians', but it was present in the three previously published ancient Rapanui with the highest depth of coverage (RN035_pre, RN041_pre and RN036_pre, by increasing order of genome coverage, Table S25). Furthermore, we detected European admixture in the two post-European-contact individuals, in agreement with ADMIXTURE and contamination estimates in [37]. However, for these two last individual samples, we also estimated non-zero African-like and East-Asian-like components, which are probably spurious signals due to their low genome coverage (Table S25).

S12.3. Discussion

We estimated a Native-American-like component (6.0%-11.4%, average 8.7%) in all 'Ancient Rapanui' sequenced in this study. Importantly, a European-like component (present in present-day Rapanui) was absent. In the case of the ancient Rapanui analysed in [37] (Section S4.6.), we found a Native-American-like component in three out of five individuals. These were also the genomes with the highest coverage and with the most sites overlapping with the SNP array dataset (Section S4.1). In S16, we estimate the Rapanui-Native American admixture event (~1394-1477 CE) likely predated the earliest individuals from [37] (dated 1450-1620 CE). These estimates fall in line with the detected Native American admixture in them. That the Native American ancestry was not apparent before might be due to, on the one hand, the usage of a different reference panel and, on the other hand, to the fact that the software *ADMIXTURE* was previously ran on the five individuals simultaneously.

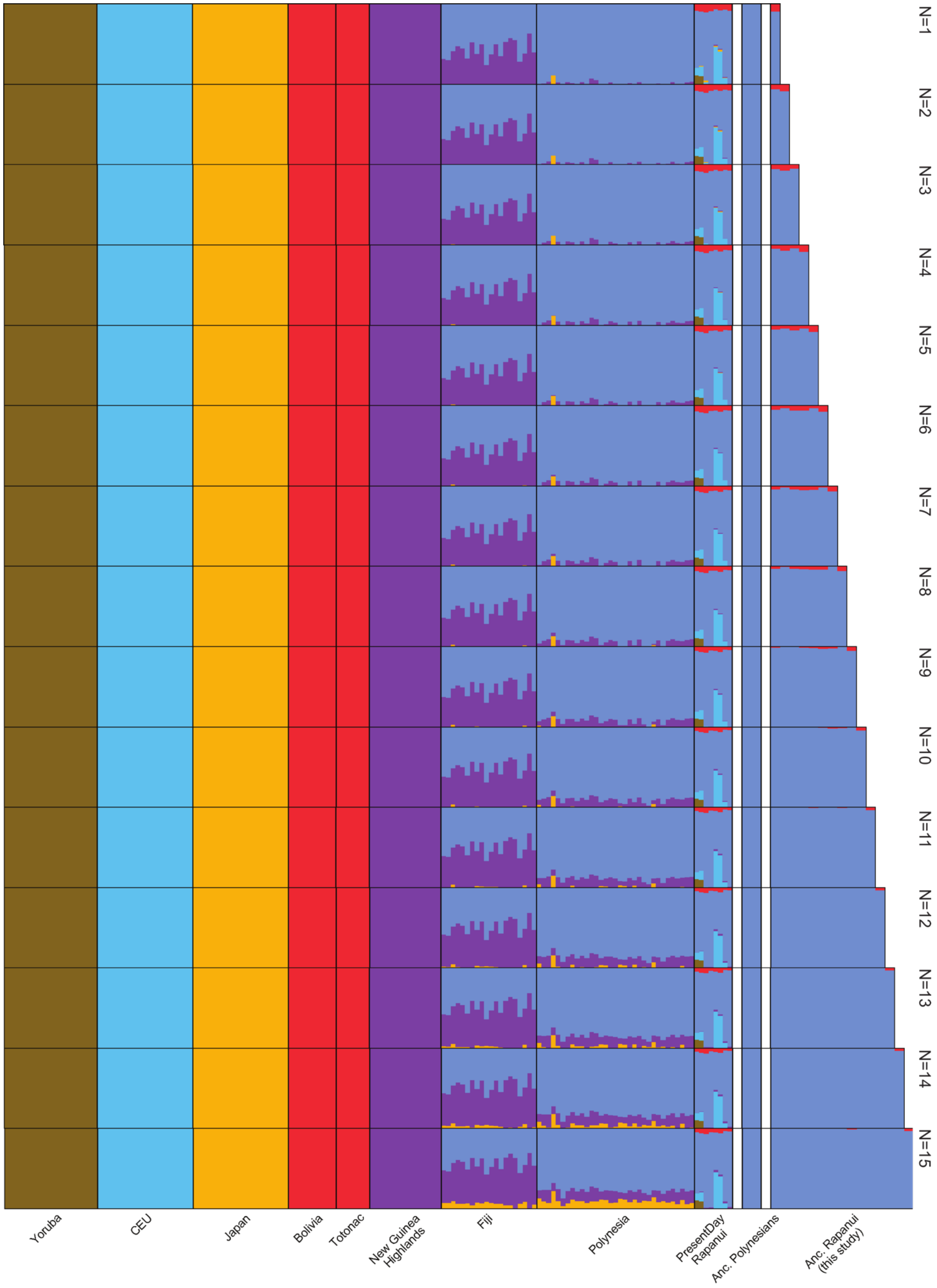
Yoruba
CEU
Japan
Bolivia
Totonac
New Guinea Highlands
Fiji
Polynesia
PresentDay Rapanui
Anc. Polynesians
Anc. Rapanui (this study)
N=1
N=2
N=3
N=4
N=5
N=6
N=7
N=8
N=9
N=10
N=11
N=12
N=13
N=14
N=15

Figure S36. *ADMIXTURE* results for present-day individuals from worldwide populations and increasing numbers of 'Ancient Rapanui' individuals. We ran *ADMIXTURE* (K=6) for 15 different datasets, which contain increasing subsets of the 'Ancient Rapanui' individuals (N=1-15). In contrast to the results shown in Figure S37, running *ADMIXTURE* with all 'Ancient Rapanui' individuals jointly, results in the Polynesian-like component becoming Rapanui-specific and thus a reduction in the Native American-like proportion in the 'Ancient Rapanui' and an increase in the Papuan- and East Asian-like proportions in other Polynesians. The ancient Polynesians include the 'Ancient Polynesians' and the 15 Rapanui sequenced in this study. Present-day Polynesians include individuals from Tuvalu, Futuna, Tokelau, Tonga, Samoa, Niue, and the Cook Islands. For this analysis, we used pseudo-haploid calls for all individuals to have a dataset as uniform as possible.

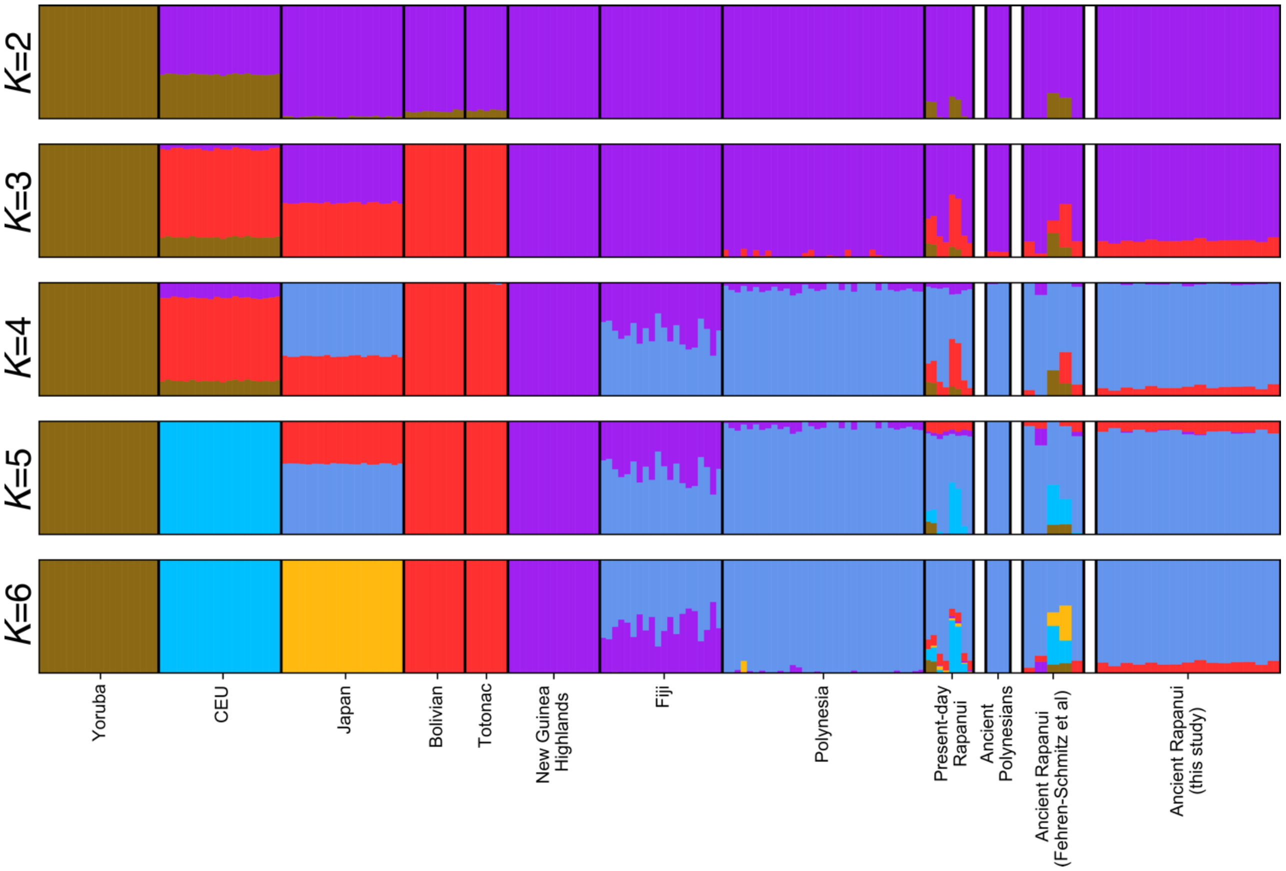

Figure S37. *ADMIXTURE* results for present-day individuals from worldwide populations and for ancient Polynesians, assuming *K*=2-6 ancestral components. To minimise the effect of strong drift in Polynesians, we estimated admixture proportions for each Polynesian individual independently (Section S12.2). That is, we ran *ADMIXTURE* by including the African (Yoruba), Europan (CEU), East Asian (Japan), American (Bolivia and Totonac), Near Oceanian (New Guinea Highlands and Fiji) reference populations and one Polynesian individual at a time. The ancient Polynesians include the 'Ancient Polynesians', the Rapanui sequenced in [37] and the 15 'Ancient Rapanui' sequenced in this study. Present-day Polynesians include individuals from Tuvalu, Futuna, Tokelau, Tonga, Samoa, Niue, and the Cook Islands. For this analysis, we used pseudo-haploid calls for all individuals to have a dataset as uniform as possible.

S13. Local ancestry inference

S13.1. Methods

Using *f*-statistics (Section S7) and *ADMIXTURE* (Section S12), we found that the 15 'Ancient Rapanui' individuals carry ~10% Native American admixture. To confirm these results and date the admixture event between the Polynesian ancestors of Rapanui and Native Americans (Section S14), we conducted local ancestry inference on the 'Ancient Rapanui' genomes and on the present-day Rapanui and 'Ancient Polynesians' for reference. To infer local Polynesian, Native American and European ancestry tracts, we followed the strategy in [46]. We subset the SNP array dataset (Section S4.1) to a set of Polynesian (*n*=33) and Native American (*n*=17) individuals without European ancestry, together with European CEU individuals (*n*=58), and combined them with imputed diploid genotypes for the target ancient and present-day Rapanui and 'Ancient Polynesian' individuals. For all ancient individuals, we subset the imputed diploid genotypes (Section S5) at the sites overlapping with the SNP array dataset (Section S4.1). We phased the diploid genotypes for all individuals in the merged dataset using *shapeit2* [110] together with the phased 1000 Genomes project Phase 3 reference dataset [62] and the HapMap genetic map [40]. Finally, we used *RFmix* [111] (together with the HapMap genetic map [40]) on the phased genotypes to infer local Polynesian, Native American and European ancestry tracts in the target individuals.

Since 'Ancient Rapanui' individuals were inferred to bear virtually no European admixture (see below and Section S7,13), we repeated the phasing and local ancestry inference steps on a reduced dataset where we only include Polynesian and Native American individuals without European ancestry. Furthermore, to confirm our results based on SNP array data, we repeated these analyses using the SGDP whole-genome dataset. In this case, since Polynesian ancestry is underrepresented in the SGDP [30], we selected Papuan (*n*=15) and Southeast Asian (Ami, Atayal, Dai, Igorot, Kinh; *n*=11) individuals to represent the two main ancestries in Polynesians [44], together with Native Americans (Chane, Karitiana, Maya, Mixe, Nahua, Piapoco, Quechua, Suruí, Zapotec; *n*=20).

In what follows, we refer to these inferred tract sets as:
- '3 sources': We infer local Polynesian, Native American and European ancestry tracts using SNP array data.
- '2 sources': We infer local Polynesian and Native American ancestry tracts using SNP array data.
- '3 sources SGDP': We infer local Papuan, Southeast Asian and Native American ancestry tracts using the SGDP whole-genome dataset.

S13.2. Concordance of local Native American ancestry tract inference

For the ancient individuals—who do not carry European admixture—we observed qualitatively concordant results across different local ancestry tract sets for the placement of Polynesian and Native American ancestry tracts. In Figure S38, we show an example of the distribution of these ancestries in representative 'Ancient Rapanui' and 'Ancient Polynesian' individuals. We highlight that we only inferred a low number of short Native

American tracts in the two 'Ancient Polynesian' individuals (<1.9% of the total genome), in agreement with the MDS (Section S6), *ADMIXTURE* (Section S12), and *f*-statistics (Section S7) results indicating they do not carry Native American ancestry. By contrast, we observed less similar results between the '3 sources' and '2 sources' tract sets for present-day Rapanui, since European admixture hampers accurate local ancestry inference using only Polynesian and Native Americans as sources.

To quantify concordance across pairs of local ancestry tract sets for each ancient individual, we recorded the genetic map positions that are covered by zero, one or two Native American tracts, concordantly, in two given sets. For the two sets based on SNP array data ('3 sources' and '2 sources'), we observed ~97% concordance for the majority of the ancient individuals (Figure S39a). Furthermore, despite the absence of an appropriate Polynesian ancestry whole-genome proxy in the SGDP dataset (Section S5), we observed ~92% concordance when we compared the whole-genome ('3 sources SGDP') and SNP array ('2 sources') tract sets (Figure S39b).

Finally, following the rationale in Section 5, we repeated the same analyses with a set of imputed diploid genotypes obtained through a modified reference panel where Peruvian individuals were excluded. In this case, we observed similar concordance values when we compared the local ancestry inference results from the two imputation runs to the SNP array ('2 sources') ancestry tract set (92% and 91%, respectively; Figure S39b,c). Overall, these results support the accuracy of the inferred Native American tracts in the 'Ancient Rapanui' (imputed diploid genotypes): 1. we infer virtually no Native American ancestry tracts in 'Ancient Polynesians', 2. we observe concordant inference of Native American ancestry tracts with SNP array and whole-genome data, 3. we infer a negligible proportion of European tracts in all ancient individuals, and 4. we observe concordant results for different imputation reference panels.

S13.3. 'Ancient Rapanui' carry ~10% Native American (and no European) admixture

We estimated the Native American and European admixture proportion in ancient and present-day Rapanui and the two 'Ancient Polynesian' individuals by aggregating the corresponding local ancestry tracts for each individual, for the '3 sources' and '2 sources' tract sets. For each individual, we estimate a 95% confidence interval for these admixture proportions using 200 replicates of a non-parametric bootstrap procedure over the 22 autosomes (Figure S40). For the 15 'Ancient Rapanui' we estimate, on average, 10% (7.9-12.3%) Native American admixture and <1% European admixture. These results are consistent with the *ADMIXTURE* and f_4-ratio admixture proportion estimates (Section S7,12).

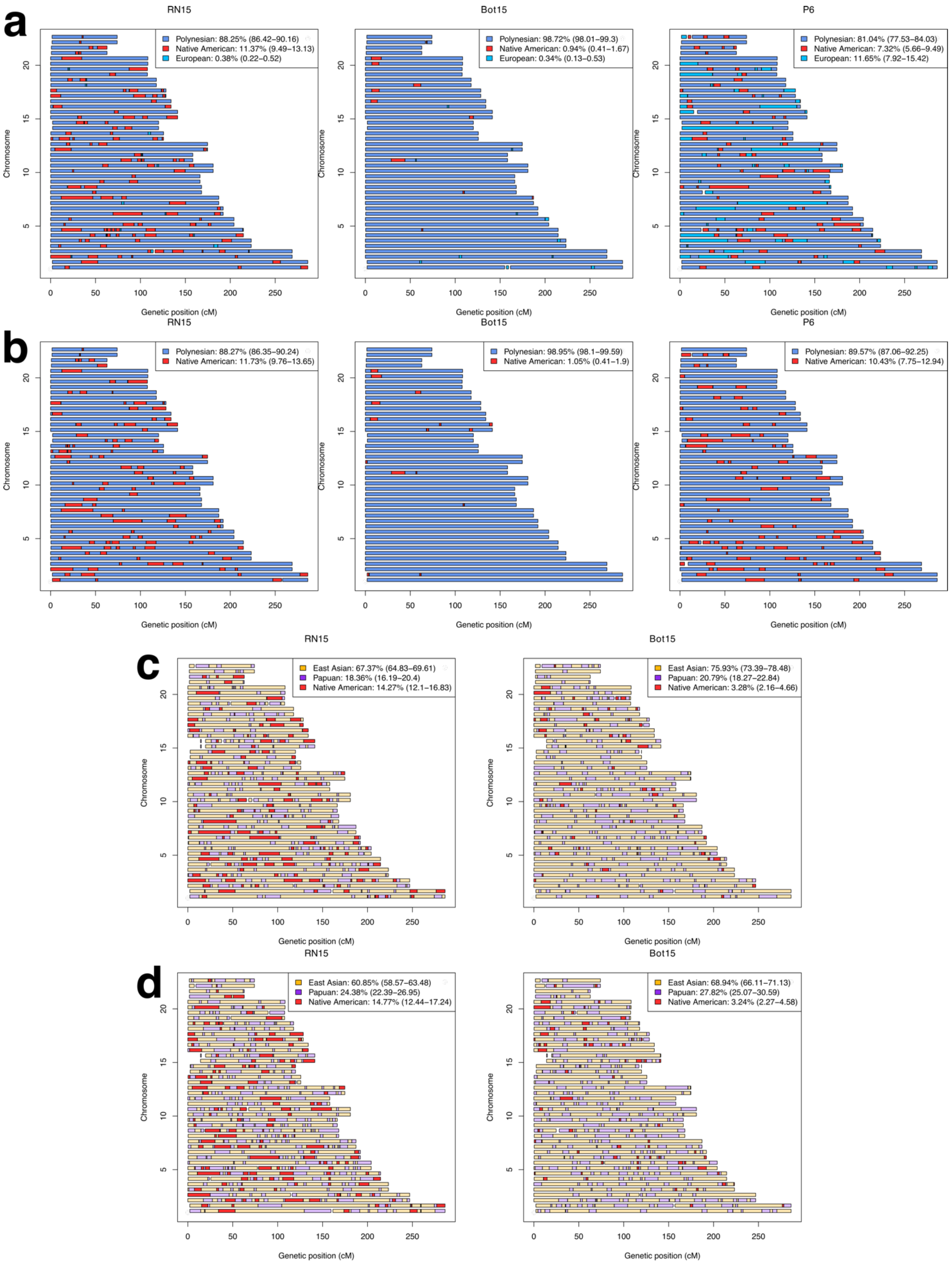

Figure S38. Local ancestry tract distributions for representative ancient and present-day Rapanui and 'Ancient Polynesian' individuals. We carried out local Polynesian, Native American and European ancestry inference using different datasets for the 15

'Ancient Rapanui' sequenced in this study (here represented by RN15), present-day Rapanui [46] (here represented by P6) and two 'Ancient Polynesian' [47] individuals (here represented by Bot15). **a.** Local ancestry distribution for the '3 sources' SNP array tract set. **b.** Local ancestry distribution obtained for the '2 sources' SNP array tract set. **c.** Local ancestry distribution for the '3 sources SGDP' tract set. **d.** Local ancestry distribution for the '3 sources SGDP' tract set, when considering diploid genotypes obtained through imputation with a reference dataset excluding Peruvian individuals (Section S5). For each individual, we show the total admixture proportion from each ancestry and confidence intervals obtained through a non-parametric bootstrap procedure (Section S13.3, Figure S40).

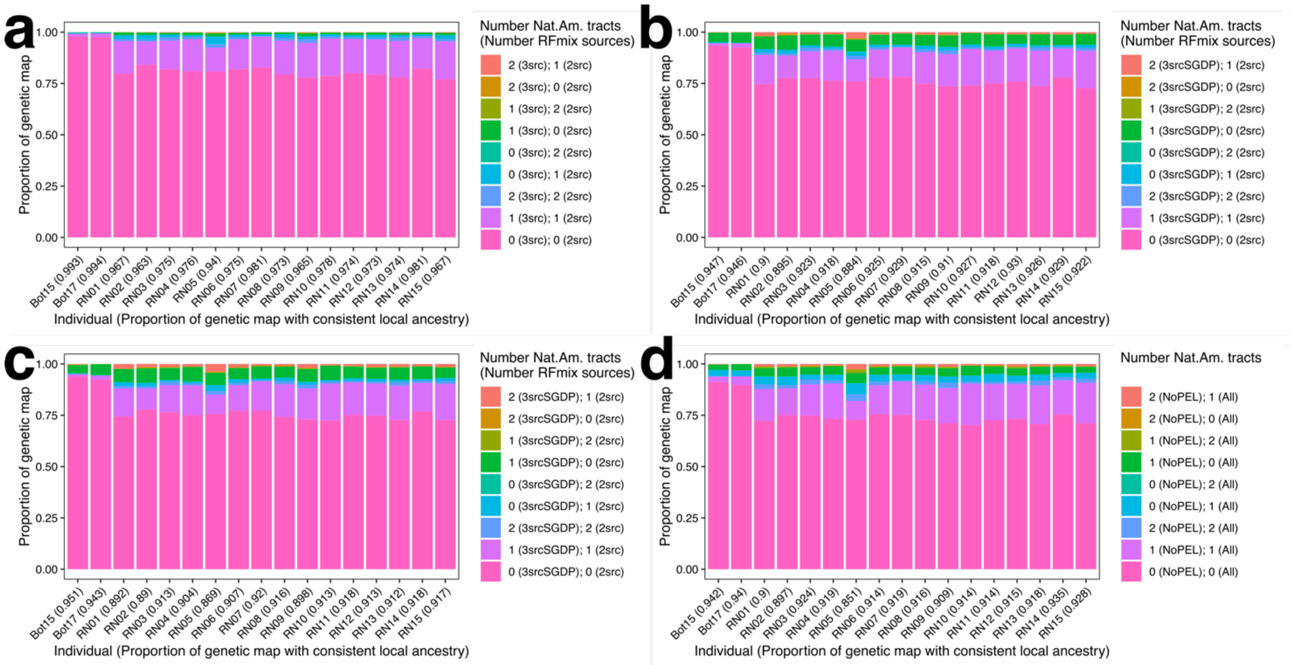

Figure S39. Concordance estimates for local Native American ancestry tract inference. For different pairs of inferred local ancestry tract sets (Section S13.1), for each ancient individual, we recorded the genetic map positions that are covered by zero, one or two Native American tracts, consistently, in both sets. **a.** Comparison between the '3 sources' and '2 sources' SNP array tract sets. **b.** Comparison between the '3 sources SGDP' and the '2 sources' SNP array tract sets. **c.** Comparison between the '3 sources SGDP' and the '2 sources' SNP array tract sets when we carried out imputation with a reference panel excluding Peruvian individuals (Section S5). **d.** Comparison between two versions of the '3 sources SGDP' tract sets with two different imputation runs (Section S5). Different colour categories represent the number of Native American tracts that are present in each tract set. For instance, the bottom component of each bar in panel *a* represents the proportion of the genetic map where a given individual carries zero Native American tracts in both tract sets. For each individual, we show in parentheses the aggregated proportion of three bottom categories, which represent the total concordance across the corresponding pair of tract sets.

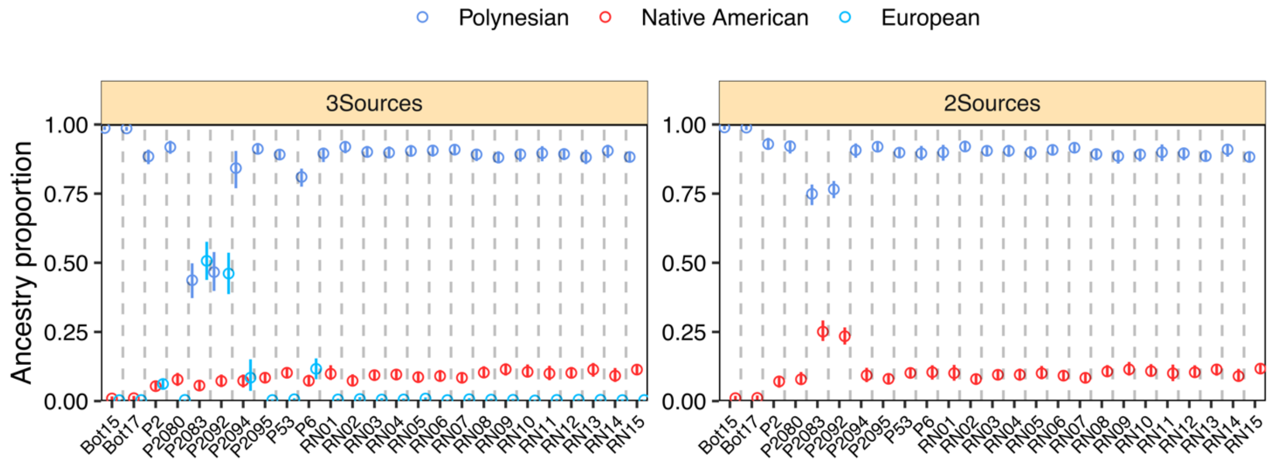

Figure S40. Estimated admixture proportions for ancient and present-day Rapanui and two 'Ancient Polynesians'. For the '3 sources' and '2 sources' SNP array tract sets, we computed the total proportion of the genome that is covered by Polynesian, Native American and European ancestry tracts. Points correspond to point estimates and error bars show a 95% confidence interval obtained using 200 replicates of a non-parametric bootstrap procedure over the 22 autosomes.

S14. Admixture dating using genetic data

Through MDS (Section S6), *f*-statistics (Section S7), *HapNe-LD* (Section S11), *ADMIXTURE* (Section S12) and local ancestry inference (Section S13), we found evidence that the 'Ancient Rapanui'—like present-day individuals from the island—carry ~10% Native American-related ancestry. To confirm that this signal is derived from an admixture event between the Polynesian ancestors of Rapanui and Native Americans, that was not mediated by Europeans, we sought for evidence of admixture linkage disequilibrium (ALD). Furthermore, we dated the admixture event using methods that rely on different features of the data.

S14.1. Admixture linkage disequilibrium-based dating

S14.1.1. Methods

To confirm a potential Rapanui-Native American admixture event, we used *ALDER* [112] and *DATES* [113] to build admixture linkage disequilibrium (ALD) curves for the 'Ancient Rapanui' genomes. Under the assumption of admixture and due to the fact that genetic markers on the same chromosome are inherited together, we expect that alleles that are more frequent in Native American populations (compared to Polynesians) are distributed nearby to each other in the 'Ancient Rapanui' genomes. In brief, ALD for pairs of SNPs at a given genetic distance is proportional to the covariance of those SNPs, weighted by the allele frequency differences between the two source populations contributing to the genomic ancestry of the admixed population [112–114], *i.e.*, Polynesians and Native Americans.

For both methods (*ALDER* and *DATES*), we modelled the ancestry of the 'Ancient Rapanui' as a mixture of a Polynesian source (represented by individuals from Tonga) on the one hand, and a set of African, European, East Asian, Papuan and Native American populations on the other, using the SNP array dataset (Section S4.1). In addition, using *ALDER*, we built ALD curves where we used the 'Ancient Rapanui' itself, as one of the source populations. To contextualise the results of both methods, we also obtained ALD curves for present-day Samoans (*ALDER*) and the 'Ancient Polynesian' individuals (*DATES*). Since these two latter Polynesian populations do not carry Native American admixture, we expect a stronger Polynesian-Native American ALD signal in the 'Ancient Rapanui'. Note that we use a different 'control' group for each method since *ALDER* requires the admixed group to include >4 individuals, whereas *DATES* can be run with as few as a single individual. Furthermore, we replicated all analyses using pseudo-haploid calls and imputed diploid genotypes for the ancient individuals.

S14.1.2. Native American admixture linkage disequilibrium in 'Ancient Rapanui'

As a first approach, we used *ALDER* to obtain ALD curves for the 'Ancient Rapanui', where the 'Ancient Rapanui' are used as one of the source populations itself, combined with other worldwide source populations. For all African and Eurasian source populations, we observed ALD curves with qualitatively similar amplitudes and decay rates. By contrast, ALD curves where the second source was a Native American population had larger amplitudes and

slower decay rates (Figure S41a,b). When we modelled the Samoan (*ALDER*) and the 'Ancient Polynesian' (*DATES*) individuals, we observed similar results for all non-Native American source populations. Nevertheless, Native American ALD curves had larger amplitude and slower decay rates for the 'Ancient Rapanui' compared to the 'Ancient Polynesians' (Figure S41a,b). In addition, when Papuans were used as a source population, ALD curves for all groups had higher amplitudes and slower decay rates compared to Africans and Eurasians. We interpret the Papuan ALD curve is related to the admixture event between Southeast Asian and Papuan populations that gave rise to the genomic ancestry of present-day Polynesians [44,103].

To investigate the possibility that the *ALDER* results using the 'Ancient Rapanui' as a source population itself are driven by strong drift [112], we computed ALD curves using two independent source populations using *ALDER* and *DATES*; the latter is optimised for ancient genome-wide data [113]. For all tests, we fixed Tongans (representing Polynesian genomic ancestry) as one of the source populations and coupled them with the same source populations that we considered for the 'single source' *ALDER* models above. In agreement with the latter, ALD curves for African and Eurasian populations were similar for the 'Ancient Rapanui', present-day Samoans and the 'Ancient Polynesians' (Figures S41c,d, S42a,c). For the three approaches (*ALDER* with one and two source populations and *DATES*), we obtained qualitatively comparable results when we considered pseudo-haploid calls and imputed diploid genotypes for the ancient individuals. Thus, these results support that the 'Ancient Rapanui' bear Native American ancestry that is not present in the 'Ancient Polynesians' or present-day Samoans. Furthermore, such ancestry—which is not consistent with having a non-Native American origin—likely entered the population through trans-Pacific contact.

To date the proposed admixture event, both ALDER and DATES rely on fitting exponentials to the estimated ALD. For the Rapanui-Native American admixture event, we infer it occurred 19-32 generations before the average date of birth for the 15 'Ancient Rapanui' individuals. Although coarse, these results are in agreement with previous estimates based on present-day individuals (1340-1445 CE) [46]. Importantly, using complementary methods (MDS (Section S6), *f*-statistics (Section S7), *ADMIXTURE* (Section S12) and local ancestry inference (Section S13)), we only find supporting evidence for an admixture event between Rapanui and Native Americans, and no other worldwide population. This result is supported by the non-informative confidence intervals we obtain when we model the 'Ancient Rapanui' as a mixture between Polynesians and any non-Native American population (Figures S41, S42b,d).

S14.2. Local ancestry tract distribution-based dating

S14.2.1. Methods

We used *tracts* [115] to obtain more accurate dates for the admixture event between the Polynesian ancestors of the Rapanui and Native Americans following [46]. Based on the inferred local ancestry tracts using '3 sources' and '2 sources' from the SNP array data

(Section S13), we built tract length distributions with 50 length bins for ancient and present-day Rapanui and the 'Ancient Polynesian' individuals. For each distribution, we obtained a 95% confidence interval using a non-parametric bootstrap approach over the 22 autosomes. For each target population, we consider two *tracts* models that include two and one admixture events, respectively. In each model, an admixture event is parametrised by an admixture time and an admixture proportion. We obtained maximum likelihood estimates (MLE) for two models:

- '3 sources': Model with four parameters. The Polynesian ancestors of the target population admix with Native Americans at time (t_{NatAm}) with a proportion (p_{NatAm}) and with Europeans at time (t_{Eur}) with a proportion (p_{Eur})
- '2 sources': Model with two parameters. The Polynesian ancestors of the target population admix with Native Americans at time (t_{NatAm}) with a proportion (p_{NatAm}).

For both models, admixture times are non-negative and are given in generations elapsed between the corresponding admixture event and the average date of birth of the individuals included in the test population. For the '3 sources' model, the order of the two admixture events is not constrained. Admixture proportions range between 0 and 1.

For every *tracts* optimisation, we initialised all parameters from ten random starting points and kept the MLE in each case. Finally, to obtain 95% confidence intervals, we obtained MLEs for each of the 500 non-parametric bootstrap replicates that we generated to build confidence intervals for the tract length distributions in Figure S43.

S14.2.2. Rapanui-Native American contact pre-dating European contact

In Figure S43, we show Polynesian, Native American and European ancestry tract length distributions for the three target populations: ancient and present-day Rapanui and 'Ancient Polynesians'. These distributions are in general agreement with the ancestry profiles that we reconstruct using other methods (Section S6,7,12). In particular, we find that both ancient and present-day Rapanui carry Native American admixture, in contrast to 'Ancient Polynesians' for which we infer a non-informative Native American tract length distribution (Figure S43). Similarly, while we obtained a European distribution enriched in longer tracts for present-day Rapanui, the European distribution was non-informative for 'Ancient Rapanui' and 'Ancient Polynesians'. That European ancestry is inferred only in present-day Rapanui and is present in longer tracts compared to Native American ancestry suggests that the latter entered the Rapanui population earlier.

Since the European tract length distribution was not informative for the two ancient groups, we explored the likelihood surface for a grid of different European admixture times and proportions, for the three target populations, for the '3 sources' model (Figure S44). For each cell of the grid, we fixed t_{Eur} and p_{Eur}, and estimated MLEs for t_{NatAm} and p_{NatAm}. As expected, and following [46], the highest likelihoods for present-day Rapanui corresponded to a region of the surface with recent t_{Eur} (3-6 generations ago) and p_{Eur}~15%. By contrast, for the 'Ancient Rapanui' and the 'Ancient Polynesians', the likelihood surface had a wide maximum that included all tested values of t_{Eur} when p_{Eur}<3%. These results support that, in contrast

to present-day Rapanui, 'Ancient Rapanui' do not carry European admixture. Thus, we rely on the '2 sources' model to estimate the Native American admixture date for the 'Ancient Rapanui', and on the '3 sources' model to estimate the Native American and European admixture dates for present-day Rapanui.

For each of the 500 bootstrap replicates, for ancient and present-day Rapanui, we estimated MLEs for the models described above and obtained the following 95% confidence intervals (Figure S45):

- 'Ancient Rapanui': t_{NatAm}=(15-17) generations before average time of birth; p_{NatAm} (0.09-0.11).
- Present-day Rapanui: t_{NatAm}=(19-21) generations before average time of birth; p_{NatAm} (0.07-0.1)%; t_{Eur}=(3-4) generations before average time of birth; p_{Eur} (0.15-0.17). These estimates recapitulate [46].

For the 'Ancient Rapanui', assuming a generation time of 29 years, we estimate their ancestors admixed with Native Americans 435-493 years before their average time of birth. While the date of birth of the individuals was unknown, we can safely assume it was before 1877 CE (collection date (Section S1,2)). Thus, we estimate 1384-1442 CE as an upper bound for the Rapanui-Native American admixture. This estimate pre-dates the European arrival in the island in 1722 CE.

Finally, to also account for the varying ages of each individual sample, we integrate these genetic estimates with radiocarbon dates (Section S2,15). As each individual had its own time of birth and death, we obtained *tracts* estimates and confidence intervals for all 15 'Ancient Rapanui' individuals separately (Figure S46) and include them in the Bayesian estimation in Section S15.

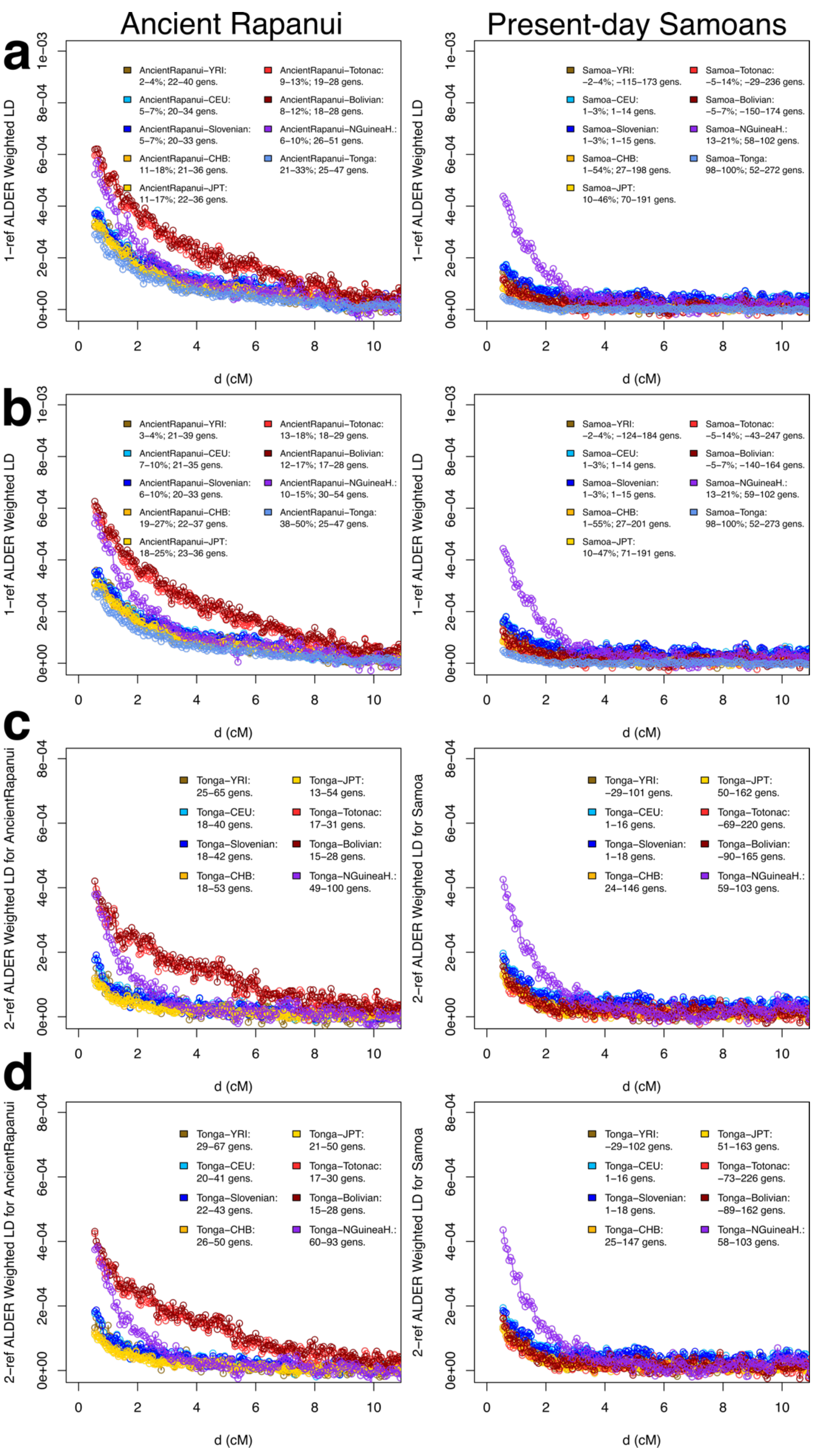

Figure S41. ***ALDER* admixture linkage disequilibrium curves and admixture time and proportion estimates for 'Ancient Rapanui' and present-day Samoans.** We modelled both target populations as a mixture of a Polynesian source population and a set of

worldwide reference populations. In each panel, estimates for 'Ancient Rapanui' are shown on the left and estimates for present-day Samoans are shown on the right. In brief, each point indicates the *ALDER* measure of linkage disequilibrium weighted by the allele frequency difference across the two corresponding source populations (*y*-axis), for all pairs of SNPs at a given genetic distance (*x*-axis). **a.** ALD curves using the target population as one of the sources itself and considering pseudo-haploid calls for ancient individuals. **b.** ALD curves using the target population as one of the sources itself and considering imputed diploid genotypes for ancient individuals. **c.** ALD curves using Tongans as the Polynesian source population and considering pseudo-haploid calls for ancient individuals. **d.** ALD curves using Tongans as the Polynesian source population and considering imputed diploid genotypes for ancient individuals. Colours indicate the non-Polynesian source population included in each model. In addition, we show estimated admixture times and proportions for each *ALDER* model.

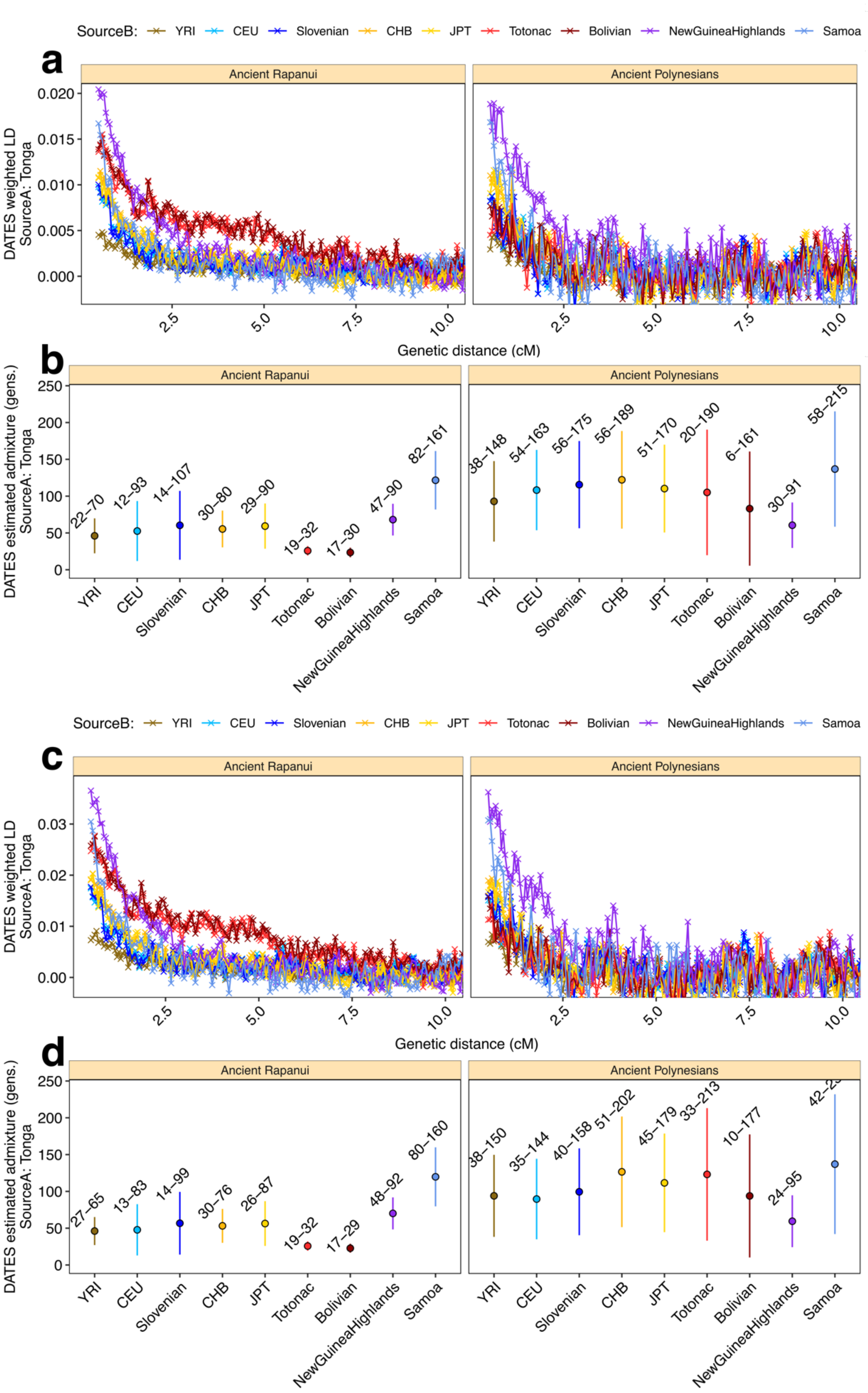

Figure S42. ***DATES* admixture linkage disequilibrium curves and admixture time and proportion estimates for 'Ancient Rapanui' and 'Ancient Polynesians'.** We modelled both target populations as a mixture of a Polynesian source population (Tongans) and a set of worldwide reference populations. In each panel, estimates for 'Ancient Rapanui' are

shown on the left and estimates for 'Ancient Polynesians' are shown on the right. In brief, each point indicates the *DATES* measure of linkage disequilibrium weighted by the allele frequency difference across the two corresponding source populations (*y*-axis), for all pairs of SNPs at a given genetic distance (*x*-axis). **a.** ALD curves considering pseudo-haploid calls for ancient individuals. **b.** *DATES* estimated admixture times and 95% confidence intervals (weighted block jackknife leaving each one of the 22 autosomes out) for each of the combinations shown in a, considering pseudo-haploid calls for ancient individuals. **c.** ALD curves considering imputed diploid genotypes for ancient individuals. **d.** *DATES* estimated admixture times and 95% confidence intervals (weighted block jackknife leaving each one of the 22 autosomes out) for each of the combinations shown in c, considering imputed diploid genotypes for ancient individuals. Colours indicate the non-Polynesian source population included in each model.

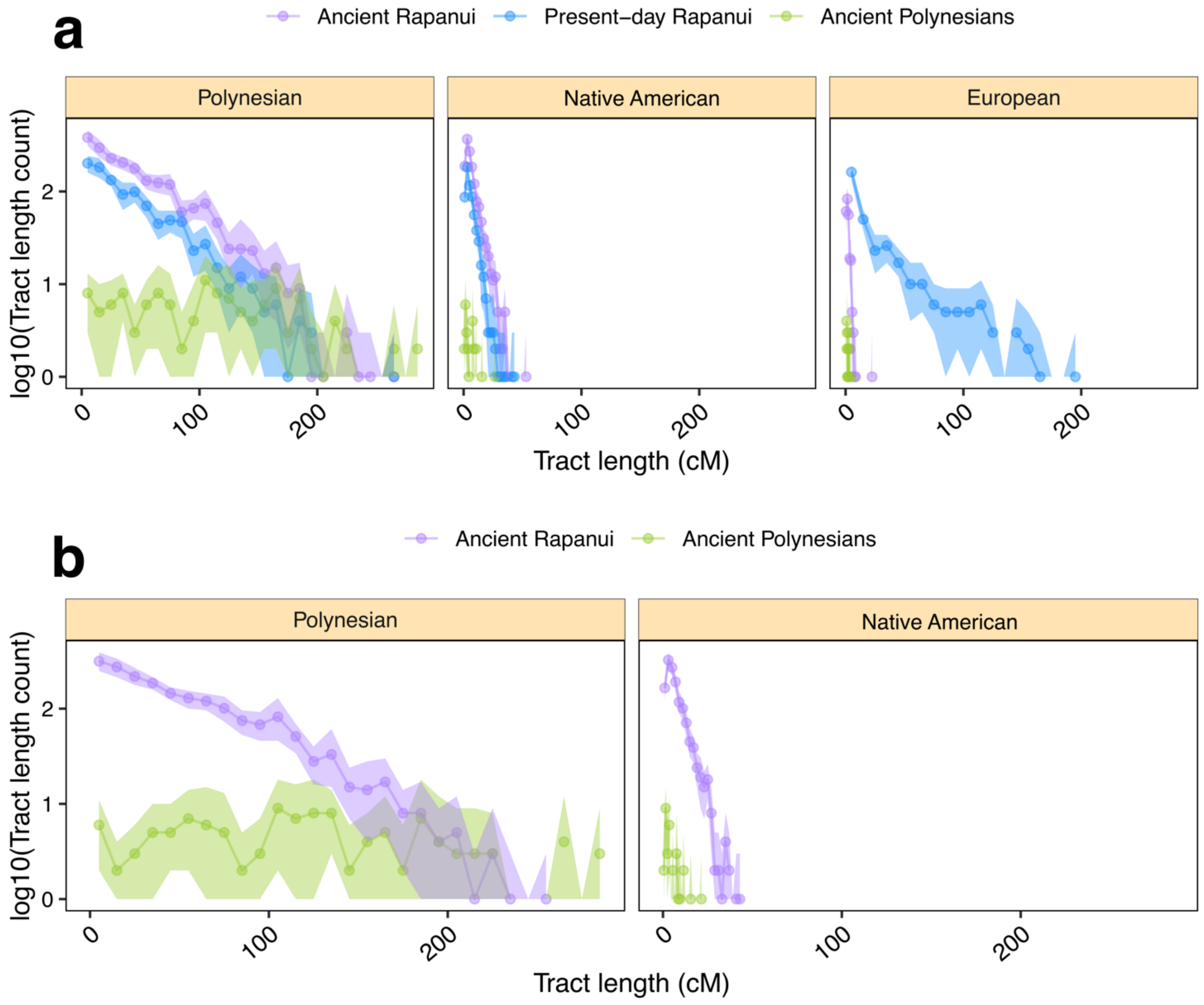

Figure S43. Polynesian, Native American and European local ancestry tract length distributions for ancient and present-day Rapanui and 'Ancient Polynesians'. We built tract length distributions with 50 bins for each ancestry, for each target population. **a.** Tract length distributions for three ancestry sources: Polynesian, Native American and European. **b.** Tract length distributions for two ancestry sources: Polynesian and Native American. Points and lines correspond to the observed data. Shaded areas correspond to a 95% confidence interval estimated from 500 non-parametric bootstrap replicates over the 22 autosomes. Colours indicate different target populations and different ancestry sources are shown in separate panels.

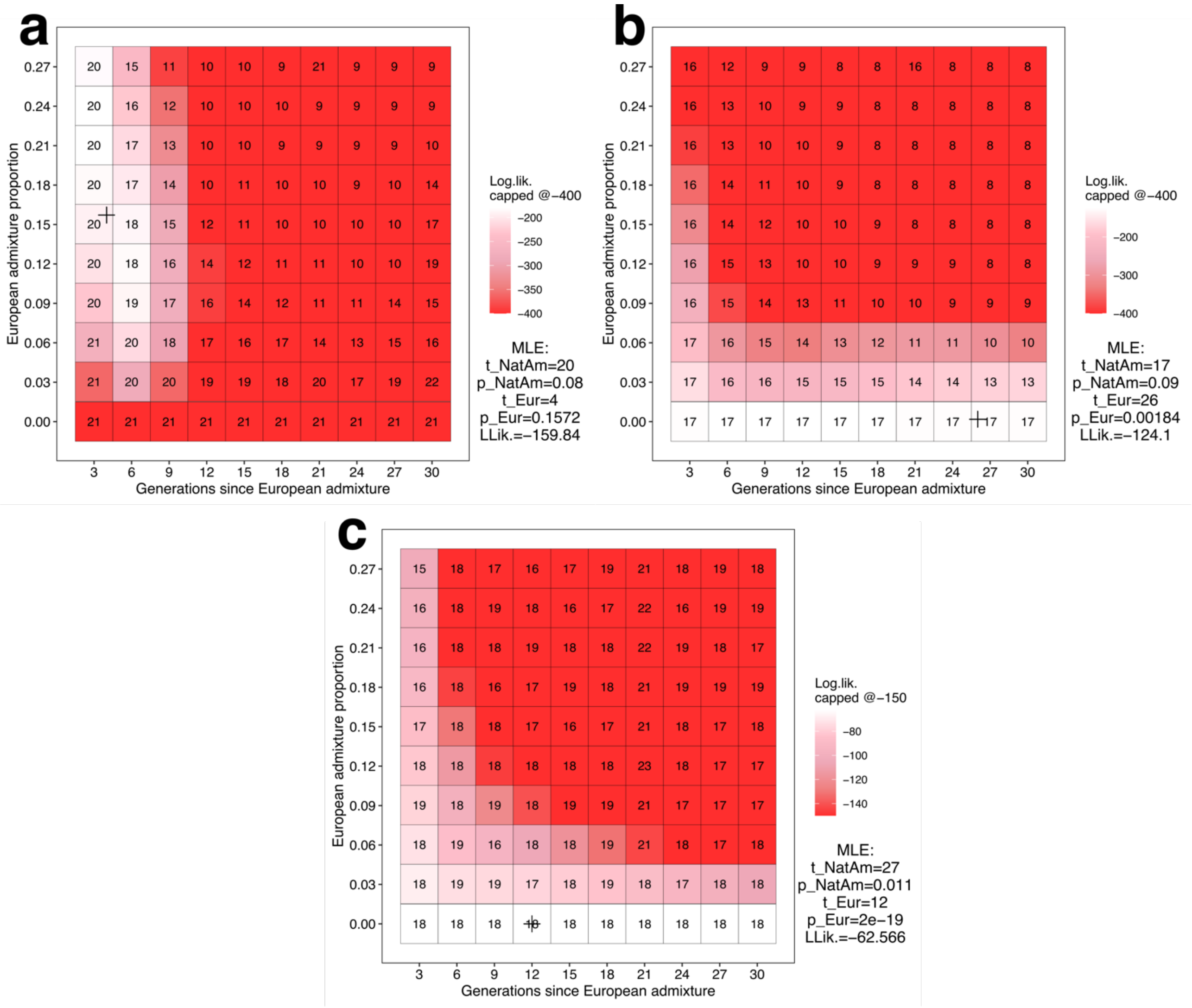

Figure S44. Likelihood surfaces for a grid of European admixture time and proportion values. Using *tracts*, we estimated the likelihood of the observed data under fixed values of t_{Eur} (x-axis) and p_{Eur} (y-axis), and estimated values of t_{NatAm} and p_{NatAm}. The colour gradient corresponds to the likelihood for each parameter set. Numbers in each cell indicate the estimated values for t_{NatAm} assuming the corresponding t_{Eur} and p_{Eur} values. MLEs for each target population are shown on the right and indicated with a cross. **a.** Likelihood surface for present-day Rapanui. **b.** Likelihood surface for 'Ancient Rapanui'. **c.** Likelihood surface for 'Ancient Polynesians'.

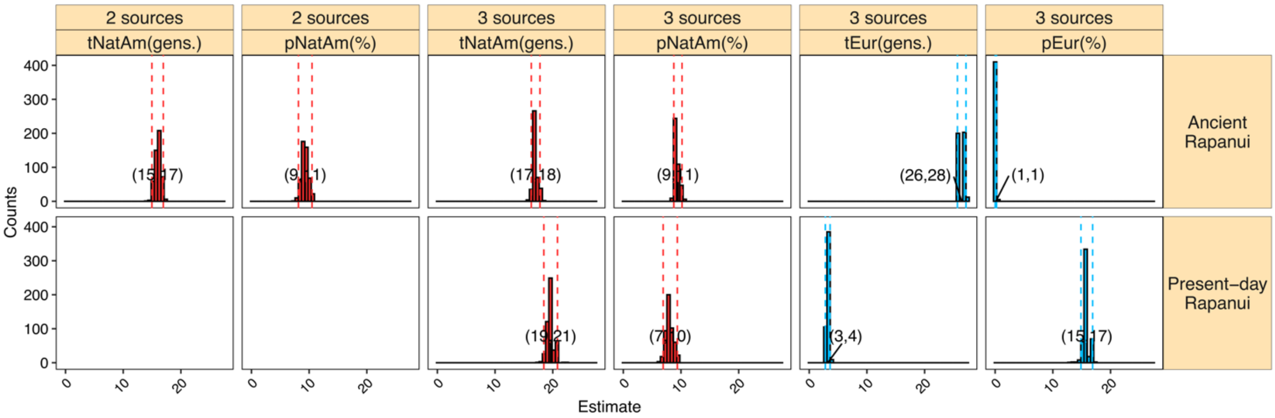

Figure S45. Non-parametric bootstrap estimate distributions for t_{NatAm}, p_{NatAm} ,t_{Eur} and p_{Eur} under different tracts models. For each target population (ancient and present-day Rapanui), we generated 500 bootstrap replicates over the 22 autosomes (Figure S35) and estimated MLEs for the '2 sources' and '3 sources' models. We plot histograms for the bootstrap estimates for each parameter. Dashed lines and numbers in parentheses correspond to 95% confidence interval upper and lower bounds.

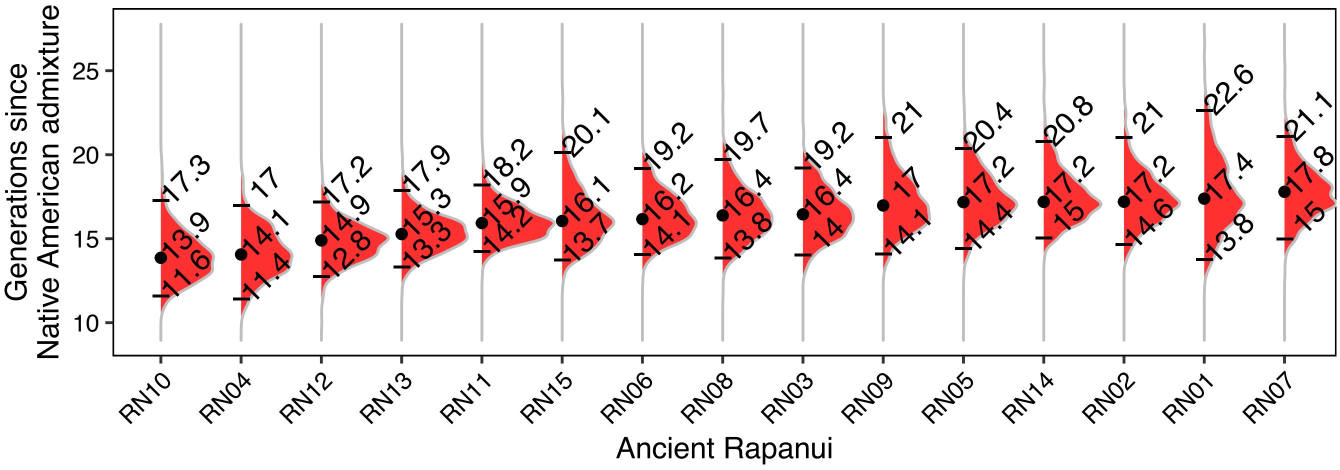

Figure S46. Native American admixture estimates for each 'Ancient Rapanui' individual. The radiocarbon dates are measured at an individual level. Hence, to be able to integrate over the radiocarbon and genetic data (Section S2,15), we also obtained *tracts* estimates for all 15 'Ancient Rapanui' individuals independently, using the '2 sources' model (Section S13). For each individual, we also obtained estimates for 500 non-parametric bootstrap replicates over the 22 autosomes to build 95% confidence intervals. We show per-individual MLEs (points), the distribution of the bootstrap estimates for the upper and lower bounds of the 95% confidence intervals (dashes).

S15. Admixture dating using genetic and C14 data jointly

We used a Bayesian statistical approach to calibrate and model the radiocarbon dates from Rapanui. We applied a novel method to estimate a date for the admixture or introgression of genetic material from the Americas using the OxCal 4.4 [11] software and the SHCAL20 calibration curve [15]. In Section S2 we described the marine carbon correction approach we applied to all of the radiocarbon dates. We describe the modelling approach below with OxCal commands given in `Courier New` font.

Using OxCal 4.4 we built a `Sequence` model using two overlapping `Phases` into which age estimates were placed. These were based on the historical date when the skeletal material was collected. We used these collection dates as *termini ante quos* (TAQ) in our models to constrain the posterior results. There are two collection dates known; 1877 and 1935. We used the `Date` command to place the calendar dates as TAQs after the end `boundary` of both `phases`. We experimented with an alternative approach of using the `Before` command instead of `Date`, but since the collection date is essentially outside the realm of the `phase` models themselves it is more appropriate to use `Date`. In any case, both methods resulted in identical posterior results.

For each dated sample we used the mean number of generations elapsed since introgression based on the per-individual *tracts* estimates (Section S14) as a constraint to calculate an 'Introgression' date in the Bayesian model. We multiplied these numbers by estimated generation times to determine a numerical estimate of the total elapsed time back to this 'Introgression' date. Initially, we estimated the generation time at 29 years (± 10% uncertainty). A total of 17 generations since introgression at 29 years per generation would give us an age since introgression of 493 years, for example. We calculated an overall uncertainty for each Introgression age based on the uncertainties in both the mean number of generations and the generation times in quadrature. Then, using the `Difference` command in OxCal, we calculated the difference between the introgression age estimate and the modelled radiocarbon posterior range for each result. We then used the `Date` function in OxCal to determine an average admixture estimate in years CE. We used a uniform prior with the `Date` function to help the model to converge, allowing it to range between 1000—1900 CE. We provide the `CQL` model codes below.

We tested the sensitivity of our modelling by varying some of the input variables. We used different per generation time estimates of 25, 29 and 30 years.

Using these methods we generated 3 models. Model 1.1 had a generation time set at 25 years. Models 2.1 and 3.1 had a generation time of 29 years and 30 years respectively. Our favoured model is Model 2.1.

The model results are given in Figures S47-49. The posterior results for each model are all virtually identical, which shows they are not sensitive to the variations in the input parameters applied. The overall agreement indices of each model were acceptably high, averaging 80%. Only OxA-35748 and BRAMS-4316 had slightly lower agreement indices in

each model. Some of the key parameters in the model are 'boundaries'. These represent a probability distribution for the beginning and end of each `phase`. Despite being undated, they provide information regarding the start and end of archaeological activity in the models. The results show that, with the constraints applied, the start `boundary` for Phase 1 for all models (1877 collection) ranges between 1675—1870 CE (at 95.4%). The start `boundary` for Phase 2 (1935 collection) ranges between 1600—1896 across the models. The end boundaries for the 1877 `Phase` and the 1935 `Phase` approximate closely the constraint applied for the collection of the skeletal remains (1877 CE for the first `Phase` models and 1935 CE for the second (at 68.3% prob.).

In Figure S50 we show the admixture estimates for each model. We observe that each generational model, whether 25, 29 or 30 year models, is statistically indistinguishable. We used the `Difference` command to determine whether there were any differences, and each distribution overlapped with zero showing no significance. For our preferred model; 2.1, the introgression date range estimated was 1336—1402 CE (68.3% prob.) and 1246—1425 CE (95.4%).

We compared these introgression estimates with the date determined for the first peopling of Rapa Nui outlined by [104] of 1150-1280 cal CE (Figure S51). We observe that there is no significant difference between the peopling estimate and the introgression `Date` at 95.4% probability for models 2.1 and 3.1 when analysed using `Difference`. This suggests that people with introgressed South American ancestry arrived in Rapa Nui around or shortly after the time the island was first peopled (Figure S52). Model 1.1, however, was significantly different. Under this model, introgression occurs 48—306 years after colonisation at 95.4% prob. This shows that the introgression estimate is sensitive to the generation time applied (25 years).

Model Codes

We include all CQL codes for the models below.

Model 1.1

```
// Delta_R values updated for Marine20
 Plot()
 {
  Curve("Marine20","Marine20.14c");
  Delta_R("LocalMarine",-214,36);
  Curve("SHCal20","shcal20.14c");
  Phase()
  {
   Sequence("1877 collection")
   {
    Boundary("Start 1");
    Phase("1")
    {
     Mix_Curve("Mixed1","SHCal20","LocalMarine",25.4,10);
     R_Date("BRAMS-4320",222,24);
     Mix_Curve("Mixed3","SHCal20","LocalMarine",28.48,10);
     R_Date("OxA-35518",196,25);
```

```
   Mix_Curve("Mixed4","SHCal20","LocalMarine",44.02,10);
   R_Date("OxA-35748",160,23);
   Mix_Curve("Mixed7","SHCal20","LocalMarine",35,10);
   R_Date("BRAMS-4316",355,24);
   Mix_Curve("Mixed8","SHCal20","LocalMarine",35,10);
   R_Date("BRAMS-4317",243,24);
   Mix_Curve("Mixed9","SHCal20","LocalMarine",35,10);
   R_Date("BRAMS-4318",250,24);
   Mix_Curve("Mixed10","SHCal20","LocalMarine",35,10);
   R_Date("BRAMS-4319",314,24);
  };
  Boundary("End 1");
  Date("1877",N(AD(1877)));
 };
 Sequence("1935 collection")
 {
  Boundary("Start 2");
  Phase("2")
  {
   Mix_Curve("Mixed2","SHCal20","LocalMarine",36.7,10);
   R_Date("BRAMS-4321",285,24);
   Mix_Curve("Mixed11","SHCal20","LocalMarine",35,10);
   R_Date("BRAMS-4322",317,24);
   Mix_Curve("Mixed5","SHCal20","LocalMarine",43.04,10);
   R_Date("OxA-35857",218,29);
   Mix_Curve("Mixed6","SHCal20","LocalMarine",33.59,10);
   R_Date("OxA-35858",236,29);
  };
  Boundary("End 2");
  Date("1935",N(AD(1935)));
 };
 };
 Date("Introgression",U(1000,1900));
 Difference("BRAMS-4316Offset","BRAMS-4316","Introgression",N(425,55));
 Difference("BRAMS-4317Offset","BRAMS-4317","Introgression",N(400,56));
 Difference("BRAMS-4318Offset","BRAMS-4318","Introgression",N(350,48));
 Difference("BRAMS-4319Offset","BRAMS-4319","Introgression",N(400,50));
 Difference("BRAMS-4320Offset","BRAMS-4320","Introgression",N(375,47));
 Difference("BRAMS-4321Offset","BRAMS-4321","Introgression",N(425,55));
 Difference("BRAMS-4322Offset","BRAMS-4322","Introgression",N(400,53));
 Difference("OxA-35518Offset","OxA-35518","Introgression",N(400,56));
 Difference("OxA-35748Offset","OxA-35748","Introgression",N(400,56));
 Difference("OxA-35857Offset","OxA-35857","Introgression",N(450,61));
 Difference("OxA-35858Offset","OxA-35858","Introgression",N(375,50));
};
```

Model 2.1

```
// Delta_R values updated for Marine20
 Plot()
 {
  Curve("Marine20","Marine20.14c");
  Delta_R("LocalMarine",-214,36);
  Curve("SHCal20","shcal20.14c");
  Phase()
  {
   Sequence("1877 collection")
   {
    Boundary("Start 1");
    Phase("1")
```

```
  {
   Mix_Curve("Mixed1","SHCal20","LocalMarine",25.4,10);
   R_Date("BRAMS-4320",222,24);
   Mix_Curve("Mixed3","SHCal20","LocalMarine",28.48,10);
   R_Date("OxA-35518",196,25);
   Mix_Curve("Mixed4","SHCal20","LocalMarine",44.02,10);
   R_Date("OxA-35748",160,23);
   Mix_Curve("Mixed7","SHCal20","LocalMarine",35,10);
   R_Date("BRAMS-4316",355,24);
   Mix_Curve("Mixed8","SHCal20","LocalMarine",35,10);
   R_Date("BRAMS-4317",243,24);
   Mix_Curve("Mixed9","SHCal20","LocalMarine",35,10);
   R_Date("BRAMS-4318",250,24);
   Mix_Curve("Mixed10","SHCal20","LocalMarine",35,10);
   R_Date("BRAMS-4319",314,24);
  };
  Boundary("End 1");
  Date("1877",N(AD(1877)));
 };
 Sequence("1935 collection")
 {
  Boundary("Start 2");
  Phase("2")
  {
   Mix_Curve("Mixed2","SHCal20","LocalMarine",36.7,10);
   R_Date("BRAMS-4321",285,24);
   Mix_Curve("Mixed11","SHCal20","LocalMarine",35,10);
   R_Date("BRAMS-4322",317,24);
   Mix_Curve("Mixed5","SHCal20","LocalMarine",43.04,10);
   R_Date("OxA-35857",218,29);
   Mix_Curve("Mixed6","SHCal20","LocalMarine",33.59,10);
   R_Date("OxA-35858",236,29);
  };
  Boundary("End 2");
  Date("1935",N(AD(1935)));
 };
};
Date("Introgression",U(800,1900));
Difference("BRAMS-4316Offset","BRAMS-4316","Introgression",N(493,57));
Difference("BRAMS-4317Offset","BRAMS-4317","Introgression",N(464,59));
Difference("BRAMS-4318Offset","BRAMS-4318","Introgression",N(406,50));
Difference("BRAMS-4319Offset","BRAMS-4319","Introgression",N(464,51));
Difference("BRAMS-4320Offset","BRAMS-4320","Introgression",N(435,49));
Difference("BRAMS-4321Offset","BRAMS-4321","Introgression",N(493,57));
Difference("BRAMS-4322Offset","BRAMS-4322","Introgression",N(464,55));
Difference("OxA-35518Offset","OxA-35518","Introgression",N(464,59));
Difference("OxA-35748Offset","OxA-35748","Introgression",N(464,59));
Difference("OxA-35857Offset","OxA-35857","Introgression",N(522,64));
Difference("OxA-35858Offset","OxA-35858","Introgression",N(435,52));
};
```

Model 3.1

```
// Delta_R values updated for Marine20
 Plot()
 {
  Curve("Marine20","Marine20.14c");
  Delta_R("LocalMarine",-214,36);
  Curve("SHCal20","shcal20.14c");
```

```
Phase()
{
 Sequence("1877 collection")
 {
  Boundary("Start 1");
  Phase("1")
  {
   Mix_Curve("Mixed1","SHCal20","LocalMarine",25.4,10);
   R_Date("BRAMS-4320",222,24);
   Mix_Curve("Mixed3","SHCal20","LocalMarine",28.48,10);
   R_Date("OxA-35518",196,25);
   Mix_Curve("Mixed4","SHCal20","LocalMarine",44.02,10);
   R_Date("OxA-35748",160,23);
   Mix_Curve("Mixed7","SHCal20","LocalMarine",35,10);
   R_Date("BRAMS-4316",355,24);
   Mix_Curve("Mixed8","SHCal20","LocalMarine",35,10);
   R_Date("BRAMS-4317",243,24);
   Mix_Curve("Mixed9","SHCal20","LocalMarine",35,10);
   R_Date("BRAMS-4318",250,24);
   Mix_Curve("Mixed10","SHCal20","LocalMarine",35,10);
   R_Date("BRAMS-4319",314,24);
  };
  Boundary("End 1");
  Date("1877",N(AD(1877)));
 };
 Sequence("1935 collection")
 {
  Boundary("Start 2");
  Phase("2")
  {
   Mix_Curve("Mixed2","SHCal20","LocalMarine",36.7,10);
   R_Date("BRAMS-4321",285,24);
   Mix_Curve("Mixed11","SHCal20","LocalMarine",35,10);
   R_Date("BRAMS-4322",317,24);
   Mix_Curve("Mixed5","SHCal20","LocalMarine",43.04,10);
   R_Date("OxA-35857",218,29);
   Mix_Curve("Mixed6","SHCal20","LocalMarine",33.59,10);
   R_Date("OxA-35858",236,29);
  };
  Boundary("End 2");
  Date("1935",N(AD(1935)));
 };
};
Date("Introgression",U(800,1900));
Difference("BRAMS-4316Offset","BRAMS-4316","Introgression",N(510,58));
Difference("BRAMS-4317Offset","BRAMS-4317","Introgression",N(480,60));
Difference("BRAMS-4318Offset","BRAMS-4318","Introgression",N(420,50));
Difference("BRAMS-4319Offset","BRAMS-4319","Introgression",N(480,52));
Difference("BRAMS-4320Offset","BRAMS-4320","Introgression",N(450,49));
Difference("BRAMS-4321Offset","BRAMS-4321","Introgression",N(510,58));
Difference("BRAMS-4322Offset","BRAMS-4322","Introgression",N(480,55));
Difference("OxA-35518Offset","OxA-35518","Introgression",N(480,60));
Difference("OxA-35748Offset","OxA-35748","Introgression",N(480,60));
Difference("OxA-35857Offset","OxA-35857","Introgression",N(540,64));
Difference("OxA-35858Offset","OxA-35858","Introgression",N(450,53));
};
```

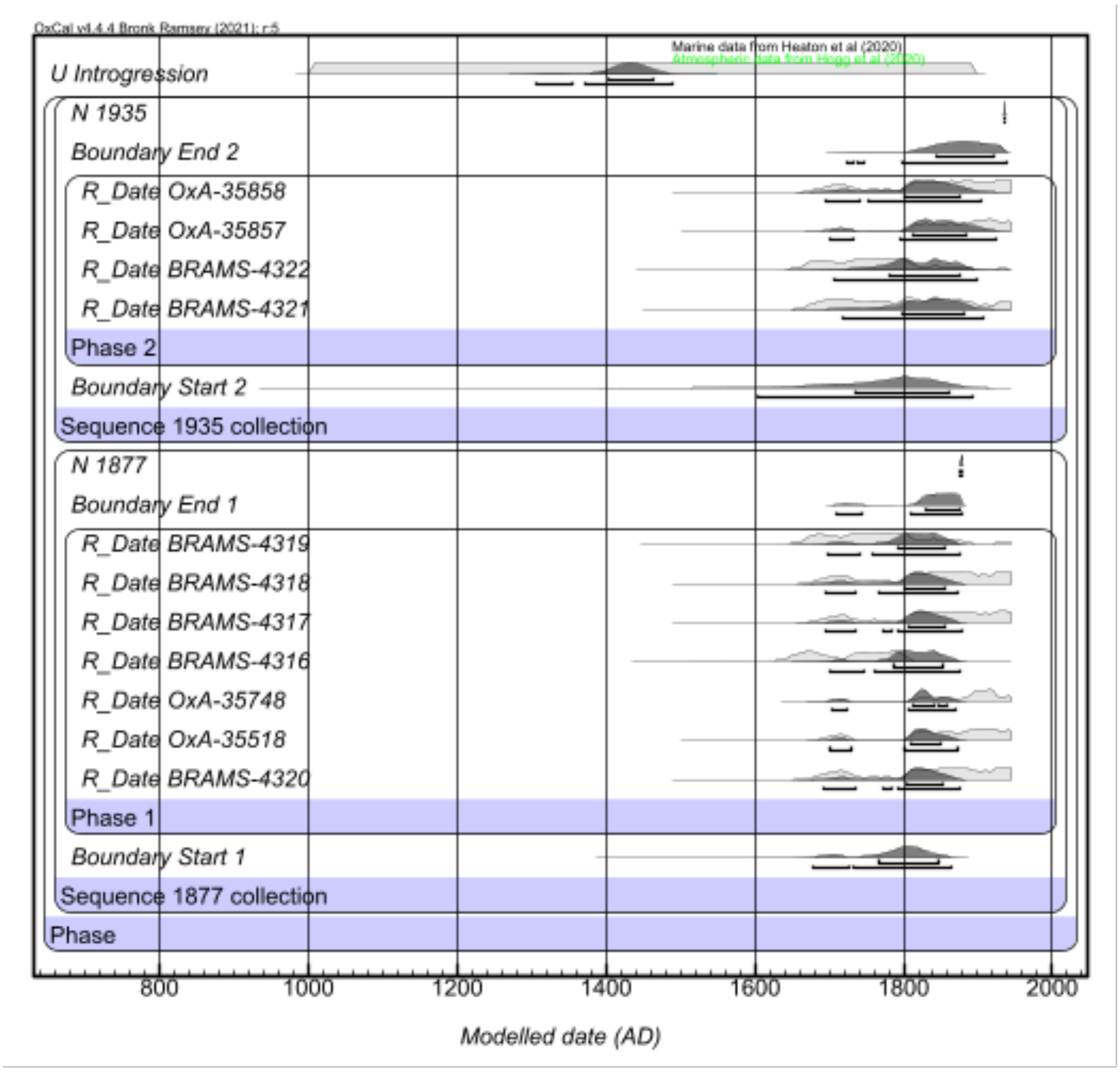

Figure S47. Bayesian model 1.1, with generation time estimated at 25 years.

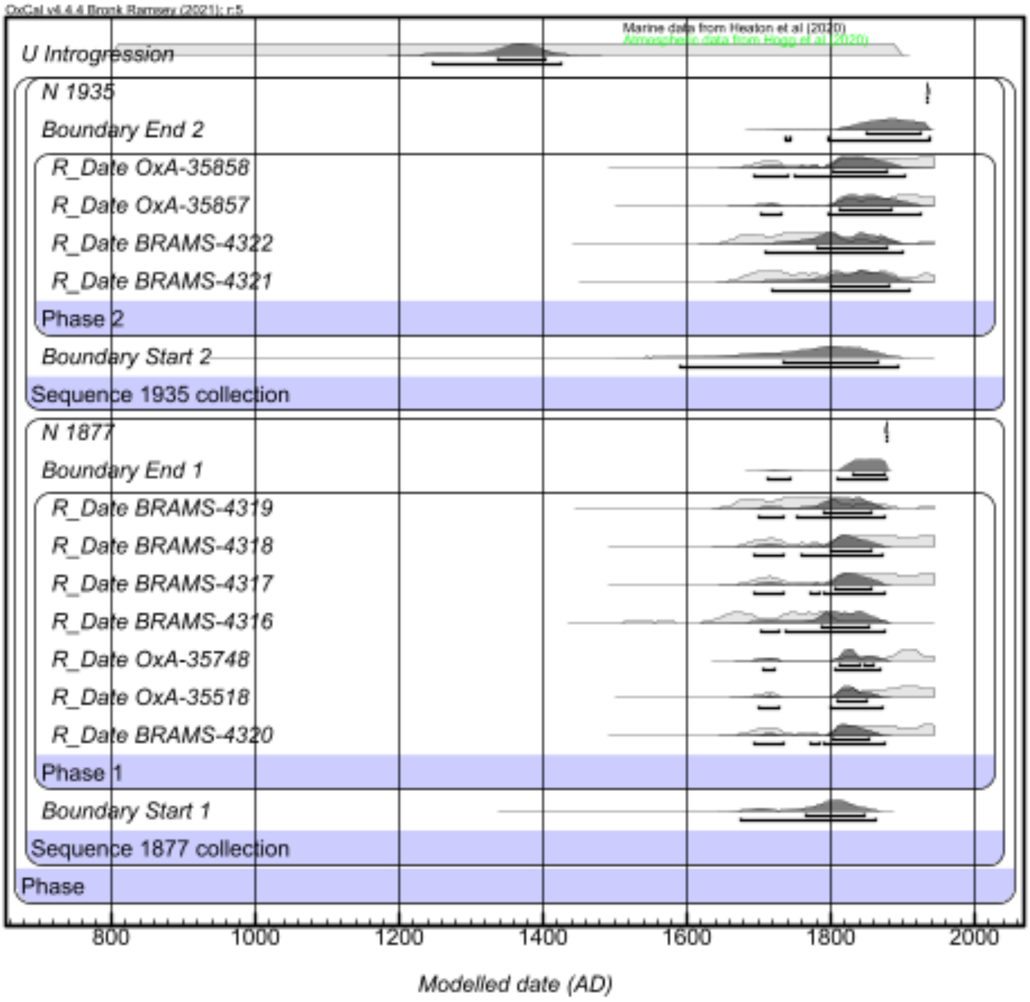

Figure S48. Bayesian model 2.1, with generation time estimated at 29 years.

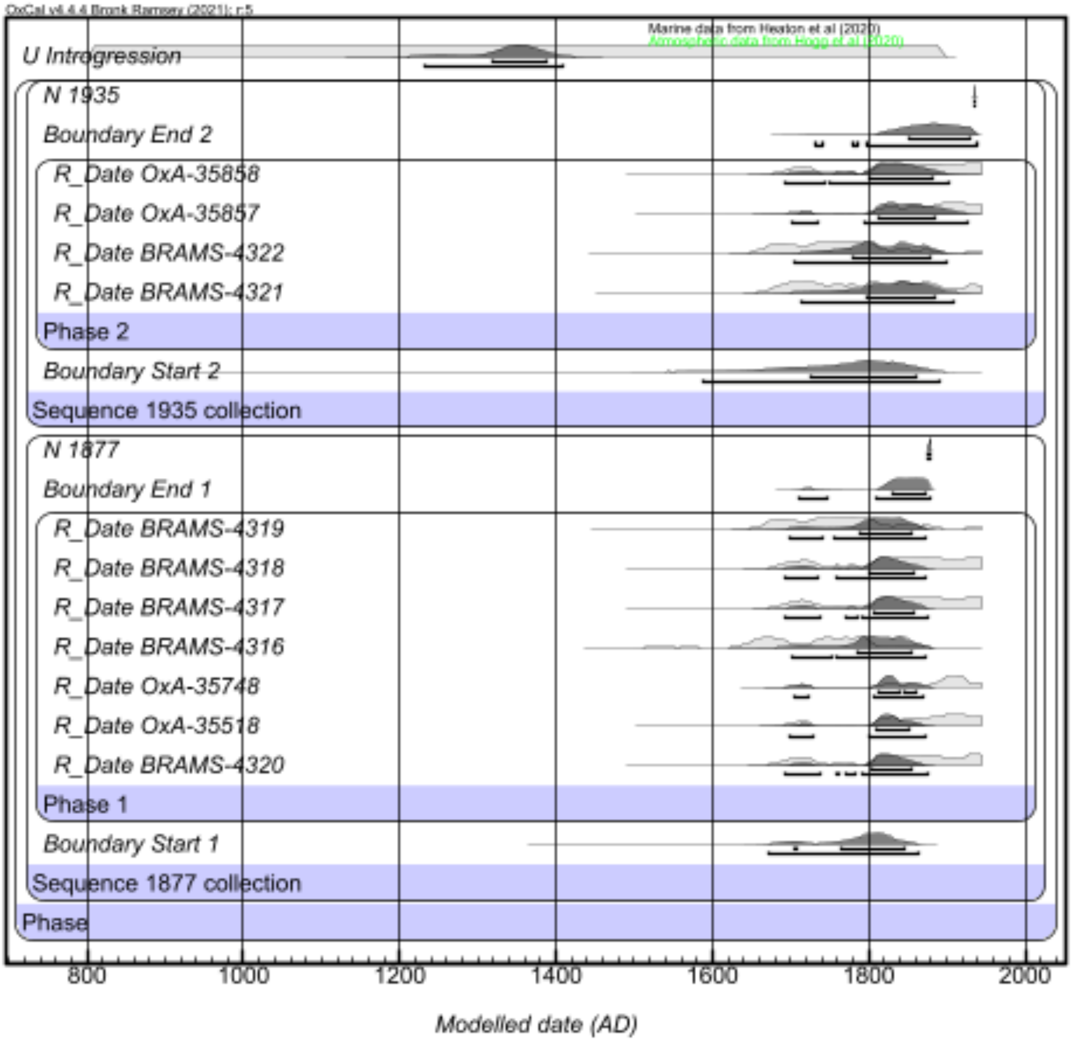

Figure S49. Bayesian model 3.1, with generation time estimated at 30 years.

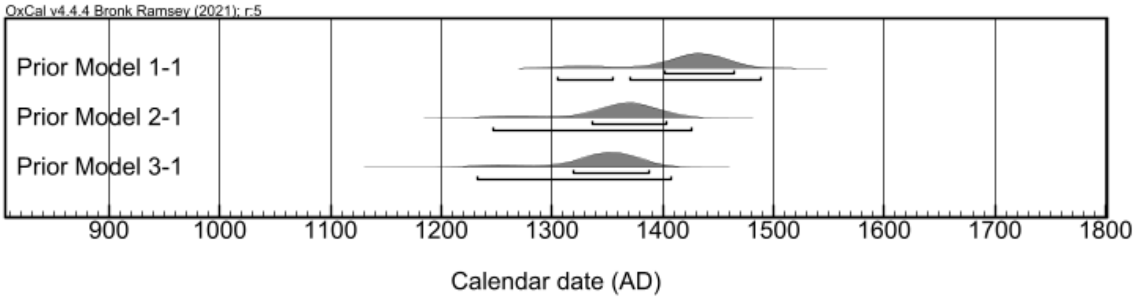

Figure S50. Comparison of the posteriors for the Introgression date estimate for each model. The results are robust to variation in the input estimates for generation times because they overlap statistically with one another.

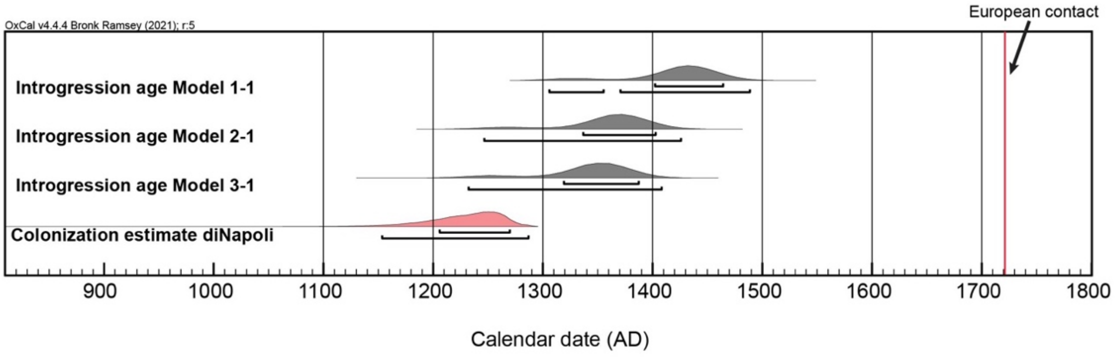

Figure S51. Comparison of the admixture estimates for each model with the Rapa Nui peopling date of [104] and the date for first European contact. The introgression estimates for models 2.1 and, 3.1 are indistinguishable from the colonization date estimate, while model 1.1 is significantly younger.

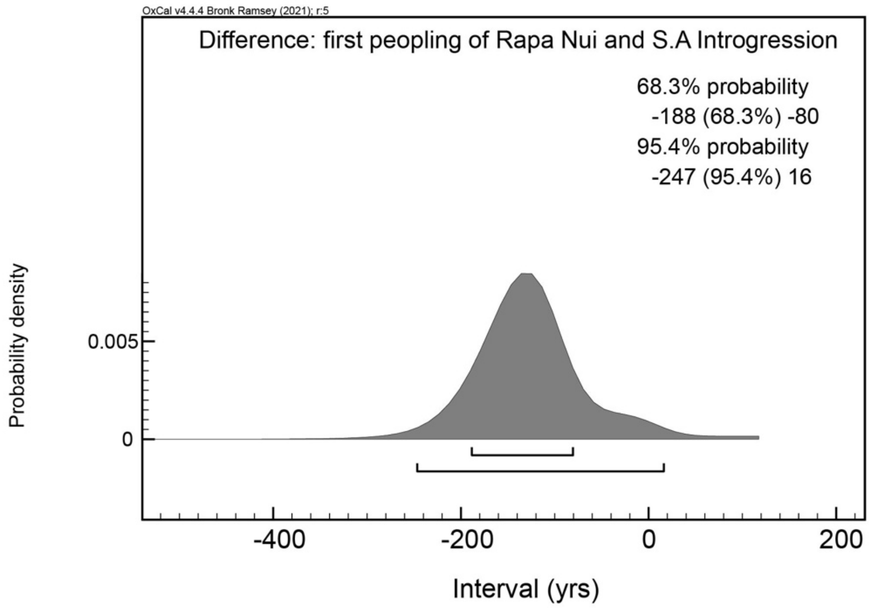

Figure S52. Probability density estimate for the `Difference` between the date for Rapa Nui peopling [104] and the Model 2.1 estimated introgression dates shown in Figure S44. At 95.4% prob. the range overlaps with zero, suggesting the two events are effectively the same age and the Model 2.1 estimated introgression dates shown in Figure S44. At 95.4% prob. the range overlaps with zero, suggesting the two events are effectively the same age.

References

1. Allentoft, M. E. *et al.* Population genomics of Bronze Age Eurasia. *Nature* **522**, 167–172 (2015).
2. Damgaard, P. B. *et al.* Improving access to endogenous DNA in ancient bones and teeth. *Scientific Reports* **5**, 11184 (2015).
3. Rohland, N. & Hofreiter, M. Ancient DNA extraction from bones and teeth. *Nature Protocols* **2**, 1756–1762 (2007).
4. Rohland, N., Harney, E., Mallick, S., Nordenfelt, S. & Reich, D. Partial uracil-DNA-glycosylase treatment for screening of ancient DNA. *Philosophical Transactions of the Royal Society B: Biological Sciences* **370**, 20130624–20130624 (2014).
5. Carøe, C. *et al.* Single-tube library preparation for degraded DNA. *Methods in Ecology and Evolution* **9**, 410–419 (2018).
6. Rohland, N. & Reich, D. Cost-effective, high-throughput DNA sequencing libraries for multiplexed target capture. *Genome Res.* **22**, 939–946 (2012).
7. Kapp, J. D., Green, R. E. & Shapiro, B. A Fast and Efficient Single-stranded Genomic Library Preparation Method Optimized for Ancient DNA. *Journal of Heredity* **112**, 241–249 (2021).
8. Kircher, M., Sawyer, S. & Meyer, M. Double indexing overcomes inaccuracies in multiplex sequencing on the Illumina platform. *Nucleic Acids Research* **40**, e3–e3 (2012).
9. Brock, F., Higham, T., Ditchfield, P. & Ramsey, C. B. Current Pretreatment Methods for AMS Radiocarbon Dating at the Oxford Radiocarbon Accelerator Unit (Orau). *Radiocarbon* **52**, 103–112 (2010).
10. Knowles, T. D. J., Monaghan, P. S. & Evershed, R. P. Radiocarbon Sample Preparation Procedures and the First Status Report from the Bristol Radiocarbon AMS (BRAMS) Facility. *Radiocarbon* **61**, 1541–1550 (2019).
11. Bronk Ramsey, C. Bayesian Analysis of Radiocarbon Dates. *Radiocarbon* **51**, 337–360 (2009).

12. Burr, G. S. *et al.* Modern and Pleistocene Reservoir Ages Inferred from South Pacific Corals. *Radiocarbon* **51**, 319–335 (2009).

13. Beck, J. W., Hewitt, L., Burr, G. S., Loret, J. & Hochstetter, F. T. Mata Ki Te Rangi: Eyes Towards the Heavens. in *Easter Island* (eds. Loret, J. & Tanacredi, J. T.) 93–111 (Springer US, Boston, MA, 2003). doi:10.1007/978-1-4615-0183-1_7.

14. Arneborg, J. *et al.* Change of Diet of the Greenland Vikings Determined from Stable Carbon Isotope Analysis and ^{14} C Dating of Their Bones. *Radiocarbon* **41**, 157–168 (1999).

15. Hogg, A. G. *et al.* SHCal20 Southern Hemisphere Calibration, 0–55,000 Years cal BP. *Radiocarbon* **62**, 759–778 (2020).

16. Jarman, C. L. *et al.* Diet of the prehistoric population of Rapa Nui (Easter Island, Chile) shows environmental adaptation and resilience. *Am J Phys Anthropol* **164**, 343–361 (2017).

17. Lindgreen, S. AdapterRemoval: Easy Cleaning of Next Generation Sequencing Reads. *BMC Research Notes* **5**, 337 (2012).

18. Moreno-Mayar, J. V. *et al.* Terminal Pleistocene Alaskan genome reveals first founding population of Native Americans. *Nature* **553**, 203–207 (2018).

19. Meyer, M. *et al.* A High-Coverage Genome Sequence from an Archaic Denisovan Individual. *Science* **338**, 222–226 (2012).

20. Li, H. & Durbin, R. Fast and accurate short read alignment with Burrows-Wheeler transform. *Bioinformatics* **25**, 1754–1760 (2009).

21. Schubert, M. *et al.* Improving ancient DNA read mapping against modern reference genomes. *BMC Genomics* **13**, 178 (2012).

22. DePristo, M. A. *et al.* A framework for variation discovery and genotyping using next-generation DNA sequencing data. *Nature Genetics* **43**, 491–498 (2011).

23. Li, H. *et al.* The Sequence Alignment/Map format and SAMtools. *Bioinformatics* **25**, 2078–2079 (2009).

24. Briggs, A. W. *et al.* Patterns of damage in genomic DNA sequences from a Neandertal. *Proceedings of the National Academy of Sciences* **104**, 14616–14621 (2007).

25. Mittnik, A., Wang, C.-C., Svoboda, J. & Krause, J. A Molecular Approach to the Sexing of the Triple Burial at the Upper Paleolithic Site of Dolní Věstonice. *PLoS ONE* **11**, e0163019 (2016).

26. Malaspinas, A.-S. *et al.* bammds: a tool for assessing the ancestry of low-depth whole-genome data using multidimensional scaling (MDS). *Bioinformatics* **30**, 2962–2964 (2014).

27. Troll, C. J. *et al.* A ligation-based single-stranded library preparation method to analyze cell-free DNA and synthetic oligos. *BMC Genomics* **20**, 1023 (2019).

28. Malaspinas, A.-S. *et al.* A genomic history of Aboriginal Australia. *Nature* (2016) doi:10.1038/nature18299.

29. Korneliussen, T. S., Albrechtsen, A. & Nielsen, R. ANGSD: Analysis of Next Generation Sequencing Data. *BMC Bioinformatics* **15**, (2014).

30. Mallick, S. *et al.* The Simons Genome Diversity Project: 300 genomes from 142 diverse populations. *Nature* (2016) doi:10.1038/nature18964.

31. Orlando, L. *et al.* Recalibrating Equus evolution using the genome sequence of an early Middle Pleistocene horse. *Nature* **499**, 74–78 (2013).

32. Fu, Q. *et al.* A Revised Timescale for Human Evolution Based on Ancient Mitochondrial Genomes. *Current Biology* **23**, 553–559 (2013).

33. Weissensteiner, H. *et al.* HaploGrep 2: mitochondrial haplogroup classification in the era of high-throughput sequencing. *Nucleic Acids Research* **44**, W58–W63 (2016).

34. Friedlaender, J. S. *et al.* The Genetic Structure of Pacific Islanders. *PLoS Genet* **4**, e19 (2008).

35. Duggan, A. T. *et al.* Maternal History of Oceania from Complete mtDNA Genomes: Contrasting Ancient Diversity with Recent Homogenization Due to the Austronesian Expansion. *The American Journal of Human Genetics* **94**, 721–733 (2014).

36. Kayser, M. *et al.* Melanesian and Asian Origins of Polynesians: mtDNA and Y Chromosome Gradients Across the Pacific. *Molecular Biology and Evolution* **23**, 2234–2244 (2006).

37. Fehren-Schmitz, L. *et al.* Genetic Ancestry of Rapanui before and after European Contact. *Current Biology* **27**, 3209-3215.e6 (2017).

38. Hagelberg, E., Quevedo, S., Turbon, D. & Clegg, J. B. DNA from ancient Easter Islanders. *Nature* **369**, 25–26 (1994).

39. Moreno-Mayar, J. V. *et al.* A likelihood method for estimating present-day human contamination in ancient male samples using low-depth X-chromosome data. *Bioinformatics* btz660 (2019) doi:10.1093/bioinformatics/btz660.

40. The International HapMap 3 Consortium. Integrating common and rare genetic variation in diverse human populations. *Nature* **467**, 52–58 (2010).

41. Martiniano, R., De Sanctis, B., Hallast, P. & Durbin, R. Placing Ancient DNA Sequences into Reference Phylogenies. *Molecular Biology and Evolution* **39**, msac017 (2022).

42. Underhill, P. A. & Kivisild, T. Use of Y Chromosome and Mitochondrial DNA Population Structure in Tracing Human Migrations. *Annual Review of Genetics* **41**, 539–564 (2007).

43. Cox, M. P. *et al.* A Polynesian Motif on the Y Chromosome: Population Structure in Remote Oceania. *Human Biology* **79**, 525–535 (2007).

44. Wollstein, A. *et al.* Demographic History of Oceania Inferred from Genome-wide Data. *Current Biology* **20**, 1983–1992 (2010).

45. Xing, J. *et al.* Toward a more uniform sampling of human genetic diversity: A survey of worldwide populations by high-density genotyping. *Genomics* **96**, 199–210 (2010).

46. Moreno-Mayar, J. V. *et al.* Genome-wide Ancestry Patterns in Rapanui Suggest Pre-European Admixture with Native Americans. *Current Biology* **24**, 2518–2525 (2014).

47. Malaspinas, A.-S. *et al.* Two ancient human genomes reveal Polynesian ancestry among the indigenous Botocudos of Brazil. *Current Biology* **24**, R1035–R1037 (2014).

48. Mathieson, I. *et al.* Genome-wide patterns of selection in 230 ancient Eurasians. *Nature* **528**, 499–503 (2015).

49. Moreno-Mayar, J. V. *et al.* Early human dispersals within the Americas. *Science* **362**, eaav2621 (2018).

50. Raghavan, M. *et al.* Upper Palaeolithic Siberian genome reveals dual ancestry of Native Americans. *Nature* **505**, 87–91 (2013).

51. Raghavan, M. *et al.* Genomic evidence for the Pleistocene and recent population history of Native Americans. *Science* **349**, aab3884–aab3884 (2015).

52. Rasmussen, M. *et al.* The ancestry and affiliations of Kennewick Man. *Nature* (2015) doi:10.1038/nature14625.

53. Rasmussen, M. *et al.* The genome of a Late Pleistocene human from a Clovis burial site in western Montana. *Nature* **506**, 225–229 (2014).

54. Skoglund, P. *et al.* Genetic evidence for two founding populations of the Americas. *Nature* **525**, 104–108 (2015).

55. Prüfer, K. *et al.* The complete genome sequence of a Neanderthal from the Altai Mountains. *Nature* **505**, 43–49 (2013).

56. de la Fuente, C. *et al.* Genomic insights into the origin and diversification of late maritime hunter-gatherers from the Chilean Patagonia. *Proceedings of the National Academy of Sciences* **115**, E4006–E4012 (2018).

57. Posth, C. *et al.* Reconstructing the Deep Population History of Central and South America. *Cell* (2018) doi:10.1016/j.cell.2018.10.027.

58. Nakatsuka, N. *et al.* A Paleogenomic Reconstruction of the Deep Population History of the Andes. *Cell* **181**, 1131-1145.e21 (2020).

59. Nakatsuka, N. *et al.* Ancient genomes in South Patagonia reveal population movements associated with technological shifts and geography. *Nat Commun* **11**, 3868 (2020).

60. Scheib, C. L. *et al.* Ancient human parallel lineages within North America contributed to a coastal expansion. *Science* **360**, 1024–1027 (2018).

61. Sousa Da Mota, B. *et al.* Imputation of ancient human genomes. *Nat Commun* **14**, 3660 (2023).

62. Auton, A. *et al.* A global reference for human genetic variation. *Nature* **526**, 68–74 (2015).

63. Taliun, D. *et al.* Sequencing of 53,831 diverse genomes from the NHLBI TOPMed Program. *Nature* **590**, 290–299 (2021).

64. Karolchik, D. The UCSC Table Browser data retrieval tool. *Nucleic Acids Research* **32**, 493D – 496 (2004).

65. Rubinacci, S., Ribeiro, D. M., Hofmeister, R. J. & Delaneau, O. Efficient phasing and imputation of low-coverage sequencing data using large reference panels. *Nat Genet* **53**, 120–126 (2021).

66. Bergström, A. *et al.* Insights into human genetic variation and population history from 929 diverse genomes. *Science* **367**, eaay5012 (2020).

67. Purcell, S. *et al.* PLINK: A Tool Set for Whole-Genome Association and Population-Based Linkage Analyses. *The American Journal of Human Genetics* **81**, 559–575 (2007).

68. Ioannidis, A. G. *et al.* Native American gene flow into Polynesia predating Easter Island settlement. *Nature* **583**, 572–577 (2020).

69. Patterson, N. *et al.* Ancient Admixture in Human History. *Genetics* **192**, 1065–1093 (2012).

70. Moreno-Mayar, J. V. FrAnTK: A Frequency-based Analysis ToolKit for efficient exploration of allele sharing patterns in present-day and ancient genomic datasets. *G3 Genes|Genomes|Genetics* jkab357 (2021) doi:10.1093/g3journal/jkab357.

71. Busing, F. M., Meijer, E. & Van Der Leeden, R. Delete-m jackknife for unequal m. *Statistics and Computing* **9**, 3–8 (1999).

72. Durand, E. Y., Patterson, N., Reich, D. & Slatkin, M. Testing for Ancient Admixture between Closely Related Populations. *Molecular Biology and Evolution* **28**, 2239–2252 (2011).

73. Ioannidis, A. G. *et al.* Paths and timings of the peopling of Polynesia inferred from genomic networks. *Nature* **597**, 522–526 (2021).

74. Hunt, T. L. & Lipo, C. P. *The Statues That Walked: Unraveling the Mystery of Easter Island.* (Free Press, New York, 2011).

75. Maude, H. E. *Slavers in Paradise: The Peruvian Slave Trade in Polynesia, 1862-1864.* (Stanford University Press, Stanford, Calif, 1981).

76. Sikora, M. *et al.* The population history of northeastern Siberia since the Pleistocene. *Nature* (2019) doi:10.1038/s41586-019-1279-z.

77. Reich, D. *et al.* Reconstructing Native American population history. *Nature* **488**, 370–374 (2012).

78. Barbieri, C. *et al.* The Current Genomic Landscape of Western South America: Andes, Amazonia, and Pacific Coast. *Molecular Biology and Evolution* **36**, 2698–2713 (2019).

79. Browning, B. L. & Browning, S. R. Detecting Identity by Descent and Estimating Genotype Error Rates in Sequence Data. *The American Journal of Human Genetics* **93**, 840–851 (2013).

80. Ringbauer, H. *et al.* Accurate detection of identity-by-descent segments in human ancient DNA. *Nat Genet* **56**, 143–151 (2024).

81. Monroy Kuhn, J. M., Jakobsson, M. & Günther, T. Estimating genetic kin relationships in prehistoric populations. *PLoS ONE* **13**, e0195491 (2018).

82. Hanghøj, K., Moltke, I., Andersen, P. A., Manica, A. & Korneliussen, T. S. Fast and accurate relatedness estimation from high-throughput sequencing data in the presence of inbreeding. *GigaScience* **8**, (2019).

83. Waples, R. K., Albrechtsen, A. & Moltke, I. Allele frequency-free inference of close familial relationships from genotypes or low-depth sequencing data. *Mol Ecol* **28**, 35–48 (2019).

84. Weir, B. S., Anderson, A. D. & Hepler, A. B. Genetic relatedness analysis: modern data and new challenges. *Nat Rev Genet* **7**, 771–780 (2006).

85. Ringbauer, H., Novembre, J. & Steinrücken, M. Parental relatedness through time revealed by runs of homozygosity in ancient DNA. *Nat Commun* **12**, 5425 (2021).

86. Cassidy, L. M. *et al.* Neolithic and Bronze Age migration to Ireland and establishment of the insular Atlantic genome. *Proc. Natl. Acad. Sci. U.S.A.* **113**, 368–373 (2016).

87. Sikora, M. *et al.* Ancient genomes show social and reproductive behavior of early Upper Paleolithic foragers. *Science* **358**, 659–662 (2017).

88. Felzke, L. F. & Moore, D. Terminologias de parentesco dos grupos da família linguística Mondé. *Bol. Mus. Para. Emílio Goeldi. Ciênc. hum.* **14**, 15–32 (2019).

89. Thorsby, E. The Polynesian gene pool: an early contribution by Amerindians to Easter Island. *Philosophical Transactions of the Royal Society B: Biological Sciences* **367**, 812–819 (2012).

90. González-Martín, A., García-Moro, C., Hernández, M. & Moral, P. Inbreeding and surnames: A projection into Easter Island's past. *Am. J. Phys. Anthropol.* **129**, 435–445 (2006).

91. Diamond, J. M. *Collapse: How Societies Choose to Fail or Succeed.* (Penguin Books, Harmondsworth, 2006).

92. Bahn, P. G. & Flenley, J. *Easter Island, Earth Island.* (Thames and Hudson, New York, N.Y, 1992).

93. Fournier, R., Reich, D. & Palamara, P. F. *Haplotype-Based Inference of Recent Effective Population Size in Modern and Ancient DNA Samples.* (2022) doi:10.1101/2022.08.03.501074.

94. Hunt, T. L. & Lipo, C. P. Late Colonization of Easter Island. *Science* **311**, 1603–1606 (2006).

95. Kelleher, J., Etheridge, A. M. & McVean, G. Efficient coalescent simulation and genealogical analysis for large sample sizes. *PLoS Comput Biol* **12**, e1004842 (2016).

96. Kong, A. *et al.* Rate of de novo mutations and the importance of father's age to disease risk. *Nature* **488**, 471–475 (2012).

97. Jónsson, H. *et al.* Parental influence on human germline de novo mutations in 1,548 trios from Iceland. *Nature* **549**, 519–522 (2017).

98. Tremblay, M. & Vézina, H. New estimates of intergenerational time intervals for the calculation of age and origins of mutations. *Am. J. Hum. Genet.* **66**, 651–658 (2000).

99. Sun, J. X. *et al.* A direct characterization of human mutation based on microsatellites. *Nat Genet* **44**, 1161–1165 (2012).

100. Yang, M. A. *et al.* 40,000-Year-Old Individual from Asia Provides Insight into Early Population Structure in Eurasia. *Current Biology* **27**, 3202-3208.e9 (2017).

101. Yang, M. A. *et al.* Ancient DNA indicates human population shifts and admixture in northern and southern China. *Science* eaba0909 (2020) doi:10.1126/science.aba0909.

102. Lipson, M. *et al.* Ancient genomes document multiple waves of migration in Southeast Asian prehistory. *Science* **361**, 92–95 (2018).

103. Posth, C. *et al.* Language continuity despite population replacement in Remote Oceania. *Nature Ecology & Evolution* **2**, 731–740 (2018).

104. DiNapoli, R. J., Rieth, T. M., Lipo, C. P. & Hunt, T. L. A model-based approach to the tempo of "collapse": The case of Rapa Nui (Easter Island). *Journal of Archaeological Science* **116**, 105094 (2020).

105. Boersema, J. J. *The Survival of Easter Island: Dwindling Resources and Cultural Resilience*. (Cambridge University Press, 2015). doi:10.1017/CBO9781139226639.

106. Hurles, M. E., Matisoo-Smith, E., Gray, R. D. & Penny, D. Untangling Oceanic settlement: the edge of the knowable. *Trends in Ecology & Evolution* **18**, 531–540 (2003).

107. Kirch, P. V. Peopling of the Pacific: A Holistic Anthropological Perspective. *Annual Review of Anthropology* **39**, 131–148 (2010).

108. Alexander, D. H., Novembre, J. & Lange, K. Fast model-based estimation of ancestry in unrelated individuals. *Genome Research* **19**, 1655–1664 (2009).

109. Lawson, D. J., Van Dorp, L. & Falush, D. A tutorial on how not to over-interpret STRUCTURE and ADMIXTURE bar plots. *Nat Commun* **9**, 3258 (2018).

110. Delaneau, O., Zagury, J.-F. & Marchini, J. Improved whole-chromosome phasing for disease and population genetic studies. *Nature Methods* **10**, 5–6 (2012).

111. Maples, B. K., Gravel, S., Kenny, E. E. & Bustamante, C. D. RFMix: A Discriminative Modeling Approach for Rapid and Robust Local-Ancestry Inference. *The American Journal of Human Genetics* **93**, 278–288 (2013).

112. Loh, P.-R. *et al.* Inferring Admixture Histories of Human Populations Using Linkage Disequilibrium. *Genetics* **193**, 1233–1254 (2013).

113. Chintalapati, M., Patterson, N. & Moorjani, P. The spatiotemporal patterns of major human admixture events during the European Holocene. *eLife* **11**, e77625 (2022).

114. Fu, Q. *et al.* Genome sequence of a 45,000-year-old modern human from western Siberia. *Nature* **514**, 445–449 (2014).

115. Gravel, S. Population Genetics Models of Local Ancestry. *Genetics* **191**, 607–619 (2012).
