# Supplementary material for: Ancient Rapanui genomes reveal resilience and pre-European contact with the Americas
Source: Nature. 2024 Sep 11;633(8029):389–97. doi: 10.1038/s41586-024-07881-4 (PMC11390480; doi:10.1038/s41586-024-07881-4)
Supplement: Supplementary file 2 — Reporting Summary [file 41586_2024_7881_MOESM2_ESM.pdf]

nature portfolio

Corresponding author(s): J. Víctor Moreno-Mayar, Anna-Sapfo Malaspinas

Last updated by author(s): Apr 30, 2024

# Reporting Summary

Nature Portfolio wishes to improve the reproducibility of the work that we publish. This form provides structure for consistency and transparency in reporting. For further information on Nature Portfolio policies, see our Editorial Policies and the Editorial Policy Checklist.

## Statistics

For all statistical analyses, confirm that the following items are present in the figure legend, table legend, main text, or Methods section.

| n/a | Confirmed | |
|---|---|---|
| ☐ | ☒ | The exact sample size (*n*) for each experimental group/condition, given as a discrete number and unit of measurement |
| ☐ | ☒ | A statement on whether measurements were taken from distinct samples or whether the same sample was measured repeatedly |
| ☐ | ☒ | The statistical test(s) used AND whether they are one- or two-sided<br>*Only common tests should be described solely by name; describe more complex techniques in the Methods section.* |
| ☐ | ☒ | A description of all covariates tested |
| ☐ | ☒ | A description of any assumptions or corrections, such as tests of normality and adjustment for multiple comparisons |
| ☐ | ☒ | A full description of the statistical parameters including central tendency (e.g. means) or other basic estimates (e.g. regression coefficient) AND variation (e.g. standard deviation) or associated estimates of uncertainty (e.g. confidence intervals) |
| ☐ | ☒ | For null hypothesis testing, the test statistic (e.g. *F*, *t*, *r*) with confidence intervals, effect sizes, degrees of freedom and *P* value noted<br>*Give P values as exact values whenever suitable.* |
| ☐ | ☒ | For Bayesian analysis, information on the choice of priors and Markov chain Monte Carlo settings |
| ☐ | ☒ | For hierarchical and complex designs, identification of the appropriate level for tests and full reporting of outcomes |
| ☐ | ☒ | Estimates of effect sizes (e.g. Cohen's *d*, Pearson's *r*), indicating how they were calculated |

*Our web collection on statistics for biologists contains articles on many of the points above.*

## Software and code

Policy information about availability of computer code

Data collection

DNA libraries were sequenced in Illumina HiSeq 4000 and NovaSeq instruments. Radiocarbon measurements were undertaken at the ORAU (Oxford Radiocarbon Accelerator Unit) and BRAMS (Bristol Radiocarbon AMS) facilities.

Data analysis

Here we list the software that was used in this work. References for each software are included in the main text and supplementary information.

- CASAVA v1.8.2. (Illumina) was used to produce base calls.
- Illumina adapter sequences were trimmed using AdapterRemoval v1.5.3.
- Filtered reads were mapped using bwa aln v0.6.2-r126 and post-processed using picard-tools v2.7.1-SNAPSHOT, GATK v3.8-1-0-gf15c1c3ef and samtools v1.12.
- Deamination patterns were explored using bamdamage (distributed together with bammds).
- Type-specific error rates were estimated using ANGSD v0.930. mtDNA contamination estimates were obtained using contamMix v1.0-5 and X-chromosome contamination estimates were obtained using contaminationX commit 60e2b58.
- mtDNA haplogroups were called using HaploGrep2.
- Y-chromosome haplogroups were called using pathPhynder v1.a.
- Diploid genotypes were directly called using bcftools v1.12.
- Imputation was conducted using GLIMPSE v1.1.1.
- The imputation reference panel was lifted over with Picard liftoverVCF v1.18.11.
- SNP data was processed using plink v1.9.20200712.
- MDS transformations were carried out using R v4.2.2, following bammds.
- f-statistics were computed using FrAnTK commit 6d61ab8.

- All maps were plotted using the ggplot2 v3.3.2 R package.
- IBD segements were called using IBDseq v04Sep15.e78 and ancIBDv0.5.
- Relatedness between individuals was estimated using READ commit f541d55 and ngsRelate v2 commitc327f744d76a17e1d17ecac88fd92b3ac82a0b07.
- Runs of homozygosity were called using plink v1.9.20200712 and hapROH v0.51a0.
- Effective population sizes were estimated using HapNe-LD v1.20220802.
- Coalescent simulations were conducted using msprime v1.2.0.
- Model-based clustering was run using ADMIXTURE v1.3.0.
- Local ancestry inference was performed using RFmix on genotypes phased with shapeit2 v2.904.3.10.0-693.11.6.el7.x86_64.
- Admixture dating was conducted using ALDER v1.03, DATES v753 and tracts v1.
- Radiocarbon date calibration and admixture dating bayesian modelling were performed using OxCal 4.4.
- Figures were plotted using R v4.2.2 (packages ggplot2 v3.3.2, ggridges v0.5.3, ggrepel v0.8.0 and RColorBrewer v1.1-3) and python v3 (packages matplotlib v3.5.3 and pandas v1.0.1).

For manuscripts utilizing custom algorithms or software that are central to the research but not yet described in published literature, software must be made available to editors and reviewers. We strongly encourage code deposition in a community repository (e.g. GitHub). See the Nature Portfolio guidelines for submitting code & software for further information.

# Data

Policy information about availability of data

All manuscripts must include a data availability statement. This statement should provide the following information, where applicable:

- Accession codes, unique identifiers, or web links for publicly available datasets
- A description of any restrictions on data availability
- For clinical datasets or third party data, please ensure that the statement adheres to our policy

Following consultation with the Comisión Asesora de Monumentos Nacionales (CAMN), the 'Ancient Rapanui' sequencing data will be made available upon request to the corresponding authors. Access requests will be managed jointly with CAMN representatives. 'Ancient Rapanui' sequencing data is not available for public posting, medical research or commercial purposes.

Publicly available data was obtained from the following sources:

- Access granted by authors: (Xing et al. 2010; Wollstein et al. 2010; Raghavan et al. 2014; Malaspinas et al. 2014; Moreno-Mayar et al. 2014; Raghavan et al. 2015; Malaspinas et al. 2016; Ioannidis et al. 2020; Ioannidis et al. 2021)
- Publicly available data in the European Nucleotide Archive: PRJEB22217(Fehren-Schmitz et al. 2017), PRJEB20398(Moreno-Mayar, Potter, et al. 2018), PRJEB29074(Moreno-Mayar, Vinner, et al. 2018), PRJEB24629(de la Fuente et al. 2018), PRJEB28961(Posth et al. 2018), PRJEB37446(Nakatsuka, Lazaridis, et al. 2020), PRJEB39010(Nakatsuka, Luisi, et al. 2020), PRJEB31736/ERP114329(Auton et al. 2015; Sousa Da Mota et al. 2023),
- Publicly available data in the Short Read Archive: SRA047577(Meyer et al. 2012), SRX381032(Rasmussen et al. 2014), SRS937952(Rasmussen et al. 2015), PRJEB25445(Scheib et al. 2018)
- Publicly available data in custom repositories: (http://cdna.eva.mpg.de/neandertal/altai/ModernHumans/bam/)(Prüfer et al. 2013), (https://sharehost.hms.harvard.edu/genetics/reich_lab/sgdp/variant_set/)(Mallick et al. 2016).

# Research involving human participants, their data, or biological material

Policy information about studies with human participants or human data. See also policy information about sex, gender (identity/presentation), and sexual orientation and race, ethnicity and racism.

| | |
|---|---|
| Reporting on sex and gender | We determined the chromosomal sex of the ancient individuals by comparing the depth of coverage in the autosomes and the X-chromosome. We highlight that our findings on chromosomal sex do not provide any information on the gender identity of the individuals we sequenced in this study. We do not carry any analyses where we stratify the data by chromosomal sex. |
| Reporting on race, ethnicity, or other socially relevant groupings | We refer to the 15 ancient individuals that were sequenced in this study as 'Ancient Rapanui'. This is based on the museum records indicating these individuals were sampled in Rapa Nui and our results showing the ancient individuals are most closely related to present-day Rapanui. For the publicly available reference data, we use labels that describe their broad continental genomic ancestry as established in their original publications. |
| Population characteristics | NA |
| Recruitment | Following strict museum guidelines, we sampled petrous bone and teeth material from 15 individuals—labelled as Rapanui—from the Musée National d'Histoire Naturelle, France, Pinart (1877) and Métraux (1935) collections using a minimally-invasive method. This included the retrieval of loose teeth whenever the remains were sufficiently well preserved and enough teeth were present (four individuals) and up to 120mg (60-120mg) of petrous bone powder in other cases (11 individuals). |
| Ethics oversight | Throughout the course of the study, we met with representatives of the Rapanui community on the island, the Comisión de Desarrollo Rapa Nui (CODEIPA) and the Comisión Asesora de Monumentos Nacionales (CAMN), where we presented our research goals and ongoing results. Both commissions voted in favour of us continuing with the research and the results of our study have been communicated to the community prior to the first submission of our manuscript. Furthermore, we presented the research project in public talks, a short video and radio interviews on the island giving us the opportunity to inquire about the questions that are most relevant to the Rapanui community. These discussions have informed the research |

topics we investigated and ultimately the results presented in this work. See a detailed description in the Ethics and Inclusion Section.

Note that full information on the approval of the study protocol must also be provided in the manuscript.

# Field-specific reporting

Please select the one below that is the best fit for your research. If you are not sure, read the appropriate sections before making your selection.

☐ Life sciences ☐ Behavioural & social sciences ☒ Ecological, evolutionary & environmental sciences

For a reference copy of the document with all sections, see nature.com/documents/nr-reporting-summary-flat.pdf

# Ecological, evolutionary & environmental sciences study design

All studies must disclose on these points even when the disclosure is negative.

| | |
|---|---|
| Study description | We radiocarbon dated and sequenced the whole-genomes from 15 ancient individuals from the Polynesian island of Rapa Nui. We use the genomic data to study the historical social structure of the Rapanui, their genetic affinities to other worldwide populations and their demographic history including their historical effective population size and admixture history. |
| Research sample | We sampled 15 individuals—labelled as Rapanui—from the Musée National d'Histoire Naturelle, France, Pinart (1877) and Métraux (1935) collections using a minimally-invasive method. |
| Sampling strategy | Sample sizes were not pre-determined. For the 15 individuals, we retrieved loose teeth whenever the remains were sufficiently well preserved and enough teeth were present (four individuals) and up to 120mg (60-120mg) of petrous bone powder in other cases (11 individuals). |
| Data collection | A detailed description of the radiocarbon and genome data collection for the 15 'Ancient Rapanui' individuals is included in Supplementary Sections 1, 2 and 3. Data collection for reference datasets, their provenance and the analyses they were included in is detailed in Supplementary Section 5. |
| Timing and spatial scale | We sampled ancestral remains that were included in the Musée National d'Histoire Naturelle, France, Pinart and Métraux collections in 1877 and 1935, respectively. |
| Data exclusions | We did not exclude any of the 15 'Ancient Rapanui' individuals from any analysis. |
| Reproducibility | For each individual, we generated high-quality whole-genome sequencing data to improve statistical power. Where applicable, we conducted population genetics analyses using different call types (pseudohaploid, called diploid and imputed diploid genotypes) and filtering loci where post-mortem damage has a stronger effect (transition polymorphisms). Furthermore, we replicated different analyses using different reference datasets. We find qualitatively concordant results across the different datasets. |
| Randomization | We did not carry out any randomisation procedure. Our study is focused on understanding the genomic diversity of the Rapanui through time. Thus, we carry out analyses per-individual or by pooling all individuals into a single population. |
| Blinding | Our study did not require any blinding procedure. Our study do not measure different group responses to, e.g., different treatments. We analyse genomic data from each individual separately or by pooling all individuals into a single 'population'. |

Did the study involve field work? ☐ Yes ☒ No

# Reporting for specific materials, systems and methods

We require information from authors about some types of materials, experimental systems and methods used in many studies. Here, indicate whether each material, system or method listed is relevant to your study. If you are not sure if a list item applies to your research, read the appropriate section before selecting a response.

## Materials & experimental systems

| n/a | Involved in the study | |
|---|---|---|
| ☒ | ☐ | Antibodies |
| ☒ | ☐ | Eukaryotic cell lines |
| ☐ | ☒ | Palaeontology and archaeology |
| ☒ | ☐ | Animals and other organisms |
| ☒ | ☐ | Clinical data |
| ☒ | ☐ | Dual use research of concern |
| ☒ | ☐ | Plants |

## Methods

| n/a | Involved in the study | |
|---|---|---|
| ☒ | ☐ | ChIP-seq |
| ☒ | ☐ | Flow cytometry |
| ☒ | ☐ | MRI-based neuroimaging |

# Palaeontology and Archaeology

Specimen provenance

The 15 'Ancient Rapanui' individuals were sampled following strict museum guidelines at the Musée National d'Histoire Naturelle, France. We met with representatives of the Rapanui community on the island, the Comisión de Desarrollo Rapa Nui (CODEIPA) and the Comisión Asesora de Monumentos Nacionales (CAMN), where we presented our research goals and ongoing results. Both commissions voted in favour of us continuing with the research and the results of our study have been communicated to the community prior to the first submission of our manuscript.

Specimen deposition

The 15 'Ancient Rapanui' individuals we sequence in this study are being kept at the Musée National d'Histoire Naturelle, Musée de l'Homme in Paris. The confirmation of the origin of these individuals through genomic analyses will inform repatriation efforts led by the Rapanui repatriation program (Ka Haka Hoki Mai Te Mana Tupuna).

Dating methods

We obtained direct radiocarbon dates from 11 individuals at the ORAU (Oxford Radiocarbon Accelerator Unit) and BRAMS (Bristol Radiocarbon AMS). All lab codes and dates are reported in Table S2. Calibration methods are detailed in Supplementary Sections 3 and 16.

☒ Tick this box to confirm that the raw and calibrated dates are available in the paper or in Supplementary Information.

Ethics oversight

Throughout the course of the study, we met with representatives of the Rapanui community on the island, the Comisión de Desarrollo Rapa Nui (CODEIPA) and the Comisión Asesora de Monumentos Nacionales (CAMN), where we presented our research goals and ongoing results. Both commissions voted in favour of us continuing with the research and the results of our study have been communicated to the community prior to the first submission of our manuscript. Furthermore, we presented the research project in public talks, a short video and radio interviews on the island giving us the opportunity to inquire about the questions that are most relevant to the Rapanui community. These discussions have informed the research topics we investigated and ultimately the results presented in this work. See a detailed description in Supplementary Section 1.

Note that full information on the approval of the study protocol must also be provided in the manuscript.
